# Airway Epithelial Cell Immunity Is Delayed During Rhinovirus Infection in Asthma and COPD

**DOI:** 10.3389/fimmu.2020.00974

**Published:** 2020-05-15

**Authors:** Punnam Chander Veerati, Niamh M. Troy, Andrew T. Reid, Ngan Fung Li, Kristy S. Nichol, Parwinder Kaur, Steven Maltby, Peter A. B. Wark, Darryl A. Knight, Anthony Bosco, Chris L. Grainge, Nathan W. Bartlett

**Affiliations:** ^1^School of Medicine and Public Health, University of Newcastle, Callaghan, NSW, Australia; ^2^Priority Research Centre for Healthy Lungs, Hunter Medical Research Institute, University of Newcastle, New Lambton Heights, NSW, Australia; ^3^Systems Immunology, Telethon Kids Institute, University of Western Australia, Perth, WA, Australia; ^4^School of Biomedical Sciences and Pharmacy, University of Newcastle, Callaghan, NSW, Australia; ^5^UWA School of Agriculture and Environment, Faculty of Science, The University of Western Australia, Perth, WA, Australia; ^6^Department of Respiratory and Sleep Medicine, John Hunter Hospital, New Lambton Heights, NSW, Australia; ^7^Department of Anesthesiology, Pharmacology and Therapeutics, University of British Columbia, Vancouver, BC, Canada; ^8^Research and Academic Affairs, Providence Health Care Research Institute, Vancouver, BC, Canada

**Keywords:** rhinovirus, interferon response, innate immunity, asthma, chronic obstructive pulmonary disease (COPD), air-liquid interface (ALI) culture, RNA sequencing

## Abstract

Respiratory viral infections, particularly those caused by rhinovirus, exacerbate chronic respiratory inflammatory diseases, such as asthma and chronic obstructive pulmonary disease (COPD). Airway epithelial cells are the primary site of rhinovirus replication and responsible of initiating the host immune response to infection. Numerous studies have reported that the anti-viral innate immune response (including type I and type III interferon) in asthma is less effective or deficient leading to the conclusion that epithelial innate immunity is a key determinant of disease severity during a rhinovirus induced exacerbation. However, deficient rhinovirus-induced epithelial interferon production in asthma has not always been observed. We hypothesized that disparate *in vitro* airway epithelial infection models using high multiplicity of infection (MOI) and lacking genome-wide, time course analyses have obscured the role of epithelial innate anti-viral immunity in asthma and COPD. To address this, we developed a low MOI rhinovirus model of differentiated primary epithelial cells obtained from healthy, asthma and COPD donors. Using genome-wide gene expression following infection, we demonstrated that gene expression patterns are similar across patient groups, but that the kinetics of induction are delayed in cells obtained from asthma and COPD donors. Rhinovirus-induced innate immune responses were defined by interferons (type-I, II, and III), interferon response factors (IRF1, IRF3, and IRF7), TLR signaling and NF-κB and STAT1 activation. Induced gene expression was evident at 24 h and peaked at 48 h post-infection in cells from healthy subjects. In contrast, in cells from donors with asthma or COPD induction was maximal at or beyond 72–96 h post-infection. Thus, we propose that propensity for viral exacerbations of asthma and COPD relate to delayed (rather than deficient) expression of epithelial cell innate anti-viral immune genes which in turns leads to a delayed and ultimately more inflammatory host immune response.

## Introduction

Asthma and chronic obstructive pulmonary disease (COPD) are chronic inflammatory airway diseases with symptoms including chronic cough, wheezing, and shortness of breath (1). Globally asthma and COPD affect nearly 339 and 328 million people with morbidity rates of ~1,000 and 877 per day, respectively (2, 3). Rhinovirus (RV) infections are the most common viral cause of disease exacerbations (4), inducing 70–80% and 22–64% of asthma and COPD exacerbations, respectively (5–10). Disease exacerbations lead to increased morbidity and mortality and significant associated healthcare costs worldwide (11, 12).

Bronchial epithelial cells (BECs) are the primary site of RV infection in the lungs (13). RV entry into BECs leads to robust host innate immune pathway activation via pattern recognition receptor (PRR) binding to viral RNA. Relevant PRRs expressed by BECs include toll-like receptor 3 (TLR3) and RNA helicases, including retinoic acid—inducible gene I (RIG-I) and melanoma differentiation—associated gene 5 (MDA5) (14). Signaling via PRRs induces IRF7-driven expression of type I and type III interferons (IFNs) (15), as well as NF-κB regulated inflammatory genes, which are key drivers of the host response to RV (16).

IFN signaling induces expression of IFN-stimulatory genes (ISGs), including *viperin, protein kinase RNA (PKR)*, and *2*′*-5*′*-oligoadenylate synthetase 1 (OAS1)*, which limit virus replication in healthy donors (17, 18). However, impaired innate immune responses following RV infection of BECs have been reported in cells isolated from donors with respiratory disease (19–25). In contrast, some studies have reported that innate immune responses following RV infection are unaltered in similar patient populations (26–28) [refer Ritchie et al. review for summary tables on epithelial IFN discrepencies (29)]. Thus, clarity is required to understand how disease status affects epithelial cell anti-viral immunity in patients with chronic respiratory disease.

Most studies assessing RV infection using BECs from asthma and COPD patients employed submerged monolayer cultures and a high multiplicity of infection (MOI ≥1) (19, 21–24, 27, 28, 30, 31). Monolayer-based culture systems consist entirely of basal cells and therefore do not recapitulate differentiated epithelial cells that are infected *in vivo*. Air-liquid interface (ALI) cultures overcome this limitation, containing differentiated ciliated and goblet epithelial cells as well as basal cells in a pseudostratified structure (32). Previous studies reported that *in vitro* differentiated airway epithelial cells were resistant to RV infection, necessitating the use of high MOI (33). As with submerged cultures, previous studies reported RV infection of ALI-differentiated epithelial cells using MOI ≥1 (31, 34). Exposure of epithelial cells to MOIs ≥1 would lead to synchronous infection of all cells in culture, which does not accurately model *in vivo* epithelial infection and associated host immune responses.

We hypothesized that the varied methods used, including epithelial monolayer cultures and high titer virus infection, and lack of time course-based kinetic analyses may underlie discrepancies in reported findings related to the role of epithelial cell innate immunity to RV. This led us to develop a very low MOI (0.001 TCID_50_ per cell) rhinovirus infection model in well-differentiated primary BECs isolated from patients with asthma and COPD, as well as healthy donors, in ALI culture. We assessed the kinetics of innate anti-viral gene induction (e.g., interferons), as well as performed detailed genome-wide expression analysis using RNA-seq to generate a unique time course transcriptomic data set. Our data showed that the epithelial cell innate anti-viral response to RV in terms of differentially expressed genes and molecular drivers was consistent across healthy, asthma and COPD donors. Innate immune deficiency in asthma and COPD was defined by delayed expression of these genes.

## Materials and Methods

### Ethics Statement and Collection of Primary BECs

All experiments were conducted in accordance with the Hunter New England Area Health Service Ethics Committee and the University of Newcastle Safety Committee (05/08/10/3.09). Primary BECs were obtained via bronchoscopy from healthy, asthma and COPD subjects (*n* = 5 donors for each group) following written informed consent. Asthma donors had moderate to severe-persistent disease, as defined by the Global Initiative for Asthma (GINA) guidelines (35) and three donors were atopic based on skin prick test positivity. COPD donors were stage III or IV as defined by Global Initiative for Obstructive Lung Disease (GOLD) guidelines (36). Clinical characteristics are in Table 1. After primary BEC collection, cells were maintained in bronchial epithelial cell growth medium (BEGM; Lonza, Switzerland) along with growth factor supplements and stored in aliquots until used for experiments in liquid nitrogen.

**Table 1:** Clinical characteristics of subjects.

	**Healthy**	**Asthmatic**	**COPD**
Number, *n*	5	5	5
Age, years (SD)	61 (8.9)	57.2 (10.5)	66.4 (4.5)
Male, *n* (%)	0 (0)	1 (20)	2 (40)
Female, *n* (%)	5 (100)	4 (80)	3 (60)
FEV1, % predicted (SD)	90 (11.2)	62.4 (22.5)	35.4 (9.1)^*^
FVC, % predicted (SD)	97.6 (12.9)	87.4 (12.6)	59 (16)^*^
FEV1/FVC (SD)	0.7 (0.1)	0.6 (0.2)	0.5 (0.2)
Daily ICS dose, beclomethasone equivalent, μg (SD)	NA	460 (49)	352 (209.5)
Atopy (SPT positive)	NA	3 (5)	NA
Severity/GOLD stage (*n*)	NA	Severe (4)	Stage III (4)
		Moderate (1)	Stage IV (1)

*ICS, Inhaled corticosteroid; FVC, Forced vital capacity; FEV1, Forced expiratory volume in 1 s; SPT, Skin prick test*.

* *Significantly different compared with healthy donors*.

### Air Liquid Interface Cultures of Primary BECs

Primary BECs were revived and expanded in T75 flasks from liquid nitrogen vials using BEGM media (Lonza, Switzerland). Following cell expansion, BECs were trypsinised and seeded in transwell inserts (Corning, United States; 2 × 10^5^ cells per insert) in ALI initial media comprised of bronchial epithelial base medium and Dulbecco's modified eagle medium (BEBM:DMEM; 50:50 ratio) containing hydrocortisone (0.1%), bovine insulin (0.1%), epinephrine (0.1%), transferrin (0.1%), bovine pituitary extract (0.4%) and ethanolamine (80 μM), MgCl_2_ (0.3 mM), MgSO_4_ (0.4 mM), bovine serum albumin (0.5 mg/mL), amphotericin B (250 μg/mL), all-trans retinoic acid (30 ng/ml), penicillin/streptomycin (2%), and recombinant human epithelial growth factor (rhEGF) (10 ng/ml) for 3–5 days until confluent. Once confluent, apical media was removed (day 0 of ALI), as previously described (37). Basal media was changed on alternative days with ALI final media, containing lower rhEGF concentrations (0.5 ng/mL).

### Rhinovirus-A1 (RV-A1) Propagation and Quantification

RV-A1 viral stock was propagated in RD-ICAM-1 cells from an in-house stock isolated from clinical samples and sequenced to confirm identity. RV-A1 was titrated by infecting RD-ICAM-1 cells with serially diluted RV-A1, followed by observations of cytopathic effects (CPE) to assess the 50% tissue culture infective doses (TCID_50_) per milliliter (24).

### Rhinovirus-A1 Infection and Sampling

ALI cultures were infected apically with RV-A1. Initially, RV-A1 stock was diluted to obtain a MOI of 0.001 and added to the apical surface of cultures for 6 h in 250 μL BEBM with supplements, 1% Insulin-Transferrin-Selenium (ITS) and 0.5% Linoleic Acid (LA). Infection media was then replaced with 500 μL fresh BEBM (with supplements) for the remainder of the experiment. Apical and basal media samples were collected at 0-, 24-, 48-, 72-, and 96-h post-infection and stored at −80°C for protein expression analysis using ELISA and cytometric bead array (CBA). Half of the transwell membrane was carefully cut from the insert and collected into RLT buffer (Qiagen, Netherlands) containing 1% 2-mercaptoethanol for downstream molecular analyses by RT-qPCR and gene expression profiling using RNA-seq. The remaining portion of the transwell membrane was fixed in 10% neutral-buffered formalin for histological analysis.

### RNA Extraction, cDNA Synthesis, and Gene Expression Analysis by Quantitative PCR

Total RNA was extracted from cells lysed with RLT buffer using the RNeasy mini kit (Qiagen, Netherlands) and quantified using Nanodrop spectrophotometer (ThermoFisher Scientific, United States). For gene expression analysis, 200 ng of total RNA was reverse transcribed into cDNA using high capacity cDNA reverse transcription kit (ThermoFisher Scientific, United States). Quantitative PCR was carried out with customized specific primers and probes (Supplementary Table 1), and normalized to 18S rRNA on an ABI 7500 Fast Real-time PCR system (Applied Biosystems, United States).

### Transcriptomic Profiling—RNA-Seq

RNA integrity was confirmed using the Bioanalyzer (Agilent, United States) for all the samples. Sequencing libraries were constructed using 500 ng of total RNA using a Tru Seq Stranded mRNA Sample Prep Kit (Illumina Inc., United States) following manufacturer's instructions. Amplified libraries were pooled in equimolar amounts and assessed on a Bioanalyzer 1000 DNA chip according to manufacturer's instructions. Library quantification was performed using the KAPA library quantification kit (KAPA Biosystems, Switzerland). Libraries were sent to the Australian Genome Research Facility for sequencing (50 bp single-end reads, Illumina HiSeq 2000). RNA seq data are available from the NCBI Gene Expression Omnibus repository (GSE146532).

Sequencing reads were aligned to the reference human genome (hg19) using HISAT2 and summarized as gene-level counts with summarize Overlaps (38). Pre- and post-alignment quality control was performed with FASTQC and SAM Stat, respectively. Genes without an official HUGO Gene Nomenclature Committee (HGCN) symbol were filtered out of the analysis. The EDA Seq package was employed to check the data for outliers using a series of quality control plots [boxplots, relative log-expression (RLE), principal component analysis (PCA)].

### Differentially Expressed Genes (DEGs)

DEGs were identified with EdgeR, which employs a negative binomial distribution to model the count data (39). Genes with very low counts (<0.5 counts per million) were filtered out of the analysis. Paired comparisons were performed with negative binomial generalized linear models to identify differentially expressed genes over the time course. The non-differentially expressed genes were used as negative control genes to estimate factors of unwanted variation in the data using RUVSeq (40), and the statistical models were adjusted for these factors. The Benjamini-Hochberg False Discovery Rate (FDR) method was employed to control for multiple testing.

### Upstream Regulator Analysis

Upstream regulator analysis was performed to infer the molecular drivers of differentially expressed genes (41). Two statistical metrics were calculated; the overlap *p*-value measures the enrichment of the differential expression signature for known targets of a given regulator. The *p*-values were adjusted for multiple testing with the multitest R package employing the FDR method. The activation Z-score measures the agreement between the direction of the observed gene expression changes (up/down regulation) and the predicted pattern based on prior experimental evidence. Regulators with an absolute activation Z-score > 2.0 and an FDR < 0.001 were deemed significant. The 20 most significant drivers are presented for each timepoint.

### Weighted Gene Co-expression Network Analysis (WGCNA)

Gene counts were transformed using the variance stabilizing transformation algorithm from the DESeq2 package (42). Unwanted variation was estimated in the data using negative control genes employing RUVseq (40) and removed using removebatcheffect algorithm in the LIMMA package (43). Non-variable genes were filtered out of the analysis employing the varianceBasedfilter function from the DCGL package. The WGCNA algorithm was used to construct a signed co-expression network (44). Separate co-expression networks were constructed for each clinical group. WGCNA analysis was performed with the following parameters (network type = signed, softpower = 10, Pearson correlation, minimum module size = 50, deepsplit = 0, merge cut height = 0.1). The overall expression of each module was summarized using the module eigengene (ME)/first principle component. Innate DB was used to identify biological pathways enriched in the modules (45). Hierarchical cluster analysis was performed using Pearson correlation and ward linkage.

### Extracellular Protein Quantification

Primary BEC ALI culture apical supernatants were assessed for IFN-β (PBL assay science, United States), IFN-λ1/3 and IL-8 (R&D systems, United States) protein levels using ELISA and MUC5AC protein by semi-quantitative ELISA as per the manufacturer's instructions. Chemokines (IP-10, CCL5) and cytokine (IL-6) were measured using cytokine bead array (BD Biosciences, United States) according to manufacturer's instructions.

### Mucin Quantification by Alcian Blue (AB)/Periodic Acid Schiff (PAS) Staining

AB/PAS staining was performed on ALI membranes to measure total mucin content in differentiated BECs. A minimum of 5 images were captured at random for each sample using Axio Imager M2 automated microscope (Carl Zeiss AG, Oberkochen, Germany). The images were assessed for mucin content using ImageJ software (ImageJ/NIH, MD, USA) by applying an identical color deconvolution and threshold for AB/PAS overlap. To obtain distinct areas with positive stain, the images were recorded and calculated to the percent of total membrane epithelial area.

### MUC5AC Quantification by Immunohistochemistry (IHC)

MUC5AC staining was performed on the section of ALI membranes using mouse monoclonal MUC5AC antibody (Abcam; #ab3649) in 1:200 dilution as primary antibody and rabbit polyclonal anti-mouse IgG H&L (Abcam; #ab6728) in 1:1,000 dilution as secondary antibody. A minimum of five images were captured at random using Axio Imager M2 automated microscope (Carl Zeiss AG, Oberkochen, Germany) and processed using imageJ software, as described above.

### Viral Capsid Protein VP2 Staining by Immunofluorescence

Immunofluorescence of viral capsid protein VP2 was determined on formalin-fixed, paraffin-embedded sections using viral capsid VP2 antibody (QED Bioscience Inc) which binds to capsid protein VP2 of HRV16, HRV1A and HRV39. RV-VP2 antibody was raised in mouse and used in 1:5,000 dilution and labeled with goat anti-mouse IgG antibody conjugated to Alexa Fluor 488 (Cell signaling Technology; #4408) in 1:1,000 dilution. Following staining, a minimum of 5 images were captured using Axio Imager M2 fluorescent microscope (Carl Zeiss AG, Oberkochen, Germany) and a representative image from each group was reported.

### Statistical Analysis

Comparisons between patient groups at a single timepoint were analyzed using the Kruskal-Wallis test and within patient groups using one-tailed Wilcoxon signed-rank test. Statistics were performed using Graph Pad Prism 8.00 software (La Jolla, California, USA). Differences were considered significant when *P* < 0.05.

## Results

### Low MOI RV-A1 Infection of ALI-Differentiated BECs

**Figure 1:**
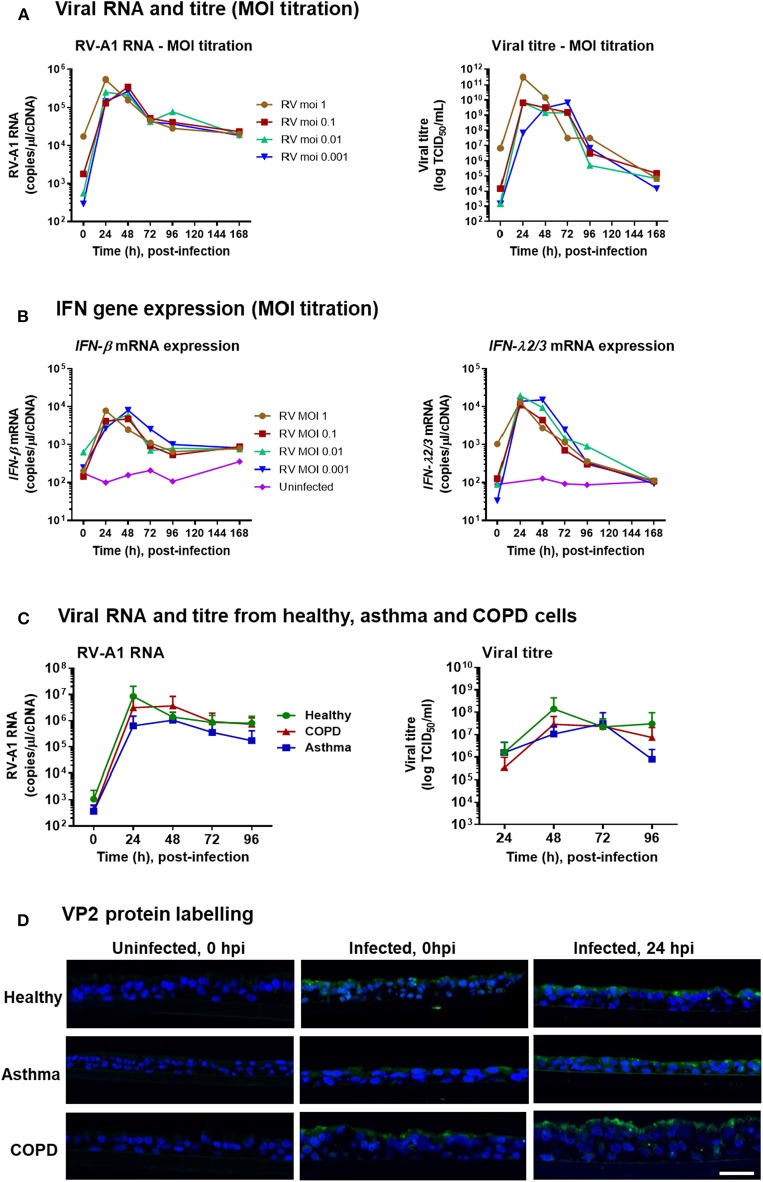
RV-A1 dose titration in differentiated BECs obtained from healthy donor and low MOI infection of differentiated BECs obtained from healthy donors, and donors with asthma or COPD. ALI cultures were infected with RV-A1 at the indicated doses, ranging from 0.001 to 1. Time course analysis was performed for viral RNA by RT-PCR and extracellular infectious virus release by TCID_50_ assay **(A)**, IFN-β gene expression and IFN-λ gene expression by RT-PCR **(B)**. Subsequent cultures were infected with RV-A1 at a MOI of 0.001 (*n* = 5 per group). Time course analysis for virus quantification based on RNA assessed by qPCR and extracellular virus by TCID_50_ assay **(C)**. Data represented as mean ± SD. Representative immunofluorescence images of RV VP2 protein staining in paraffin-embedded sections **(D)**. Scale bar: 25 μm.

To assess whether low MOI RV exposure could infect ALI-differentiated BECs, we initially performed a titration of RV-A1 doses (MOI from 1 to 0.001 TCID_50_/cell) on differentiated BECs obtained from a healthy donor. For all MOIs assessed, peak viral RNA level was detected at 24–48 h post-infection (hpi) (Figure 1A), and peak viral titer was observed at 24–72 hpi (Figure 1A). Viral RNA and titres decreased but remained detectable through 168 hpi for all MOIs (Figure 1A). In response to infection, *IFN-*β and *IFN-*λ mRNA expression were induced at all MOIs, with peak induction observed at 24–48 hpi, declining to baseline (for *IFN-*λ) by 168 hpi (Figure 1B).

### IFN Responses Are Delayed Following RV-A1 Infection in BECs Obtained From Asthma and COPD Donors

Having demonstrated that low MOI RV exposure was sufficient to establish infection and induce IFN responses in ALI-differentiated BECs from a healthy donor, we next assessed infection in differentiated cultures from donors with asthma or COPD (*n* = 5 per group) vs. healthy controls (*n* = 5; clinical characteristics in Table 1). Following RV-A1 infection (MOI 0.001), there was no significant difference in the levels of viral RNA between groups (Figure 1C). Viral titer quantification by TCID_50_ assay demonstrated production of live virus in all cultures, again with no significant difference observed between groups (Figure 1C). We also assessed localization of RV capsid protein VP2 by immunofluorescence imaging. VP2 was detected on the BEC cell surface upon exposure (0 hpi) and within cells at 24 hpi (Figure 1D).

**Figure 2:**
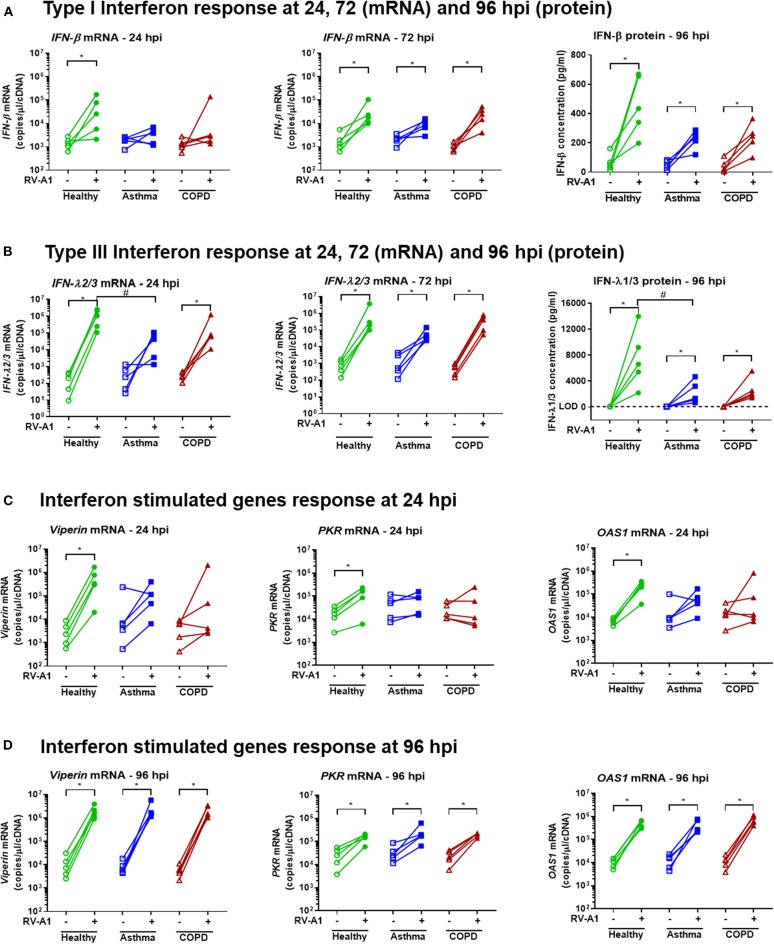
Delayed IFN responses following RV-A1 infection in differentiated BEC cultures generated from asthma or COPD donors, compared to healthy donors. Total RNA and apical culture supernatants were isolated from infected cultures at the timepoints indicated (*n* = 5 per group). Type I IFN (IFN-β) gene expression at 24 and 72 hpi, and protein levels at 96 hpi **(A)**, type III IFN (IFN-λ) gene expression at 24 and 72 hpi, and protein levels at 96 hpi **(B)**, ISGs (viperin, PKR and OAS1) expression at 24 hpi **(C)** and 96 hpi **(D)**. Data represented as individual points and analyzed using one-tailed Wilcoxon signed-rank test within the group, *p < 0.05 and Kruskal-Wallis test between groups, ^#^*p* < 0.05.

We next quantified levels of anti-viral innate immune gene expression. At 24 hpi, *IFN-*β and *IFN-*λ expression were increased in RV-infected cultures from healthy donors (Figures 2A,B). In contrast, infected cultures from asthmatic donors did not significantly up-regulate *IFN-*β or *IFN-*λ gene expression at the same timepoint (Figures 2A,B). Further, expression of *IFN-*λ mRNA was significantly lower in cultures from asthma donors, compared to healthy donors at 24 hpi (Figure 2B). In BEC cultures obtained from COPD donors, *IFN-*β gene expression was not induced following RV-A1 infection at 24 hpi. However, unlike asthma, *IFN-*λ gene expression was increased (Figures 2A,B). At 72 hpi, *IFN-*β and *IFN-*λ gene expression were increased in all disease groups following RV-A1 infection, and not different to uninfected controls (Figures 2A,B). There was a trend toward reduced IFN-β and IFN-λ protein levels at 96 hpi in RV-infected BEC cultures from asthma and COPD donors, compared to healthy donors, although this was only statistically significant for IFN-λ in COPD donor cultures (Figures 2A,B). We also quantified ISG expression, including *viperin, PKR*, and *OAS1*. ISG expression was increased in cultures generated from healthy donors following RV-A1 infection at 24 hpi (Figure 2C). In contrast, cultures from asthma and COPD donors did not exhibit ISG induction at this timepoint (Figure 2C). At 96 hpi, ISG expression was induced in all groups, with no significant differences between groups (Figure 2D).

**Figure 3:**
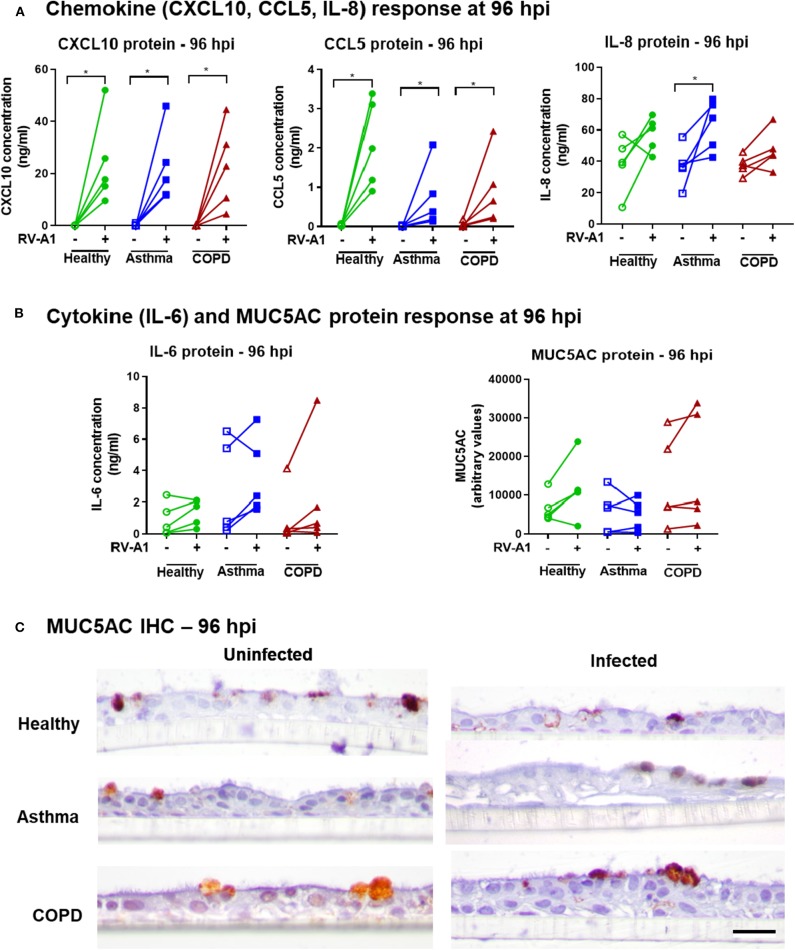
Chemokine, cytokine and MUC5AC responses in differentiated BECs following RV-A1 infection. Quantification of chemokines (CXCL10, CCL5, IL-8) **(A)**, cytokine (IL-6) and MUC5AC **(B)** protein levels in apical culture supernatants from BECs at 96 hpi. CXCL10, CCL5, and IL-6 were quantified by cytometric bead array and IL-8 and MUC5AC by ELISA. Cellular MUC5AC protein visualized by staining with anti-MUC5AC antibody **(C)**, Scale bar: 25 μm. Biological replicates are represented as individual points (*n* = 5) and analyzed using Wilcoxon signed-rank test within the group (*p < 0.05) and between groups using Kruskal-Wallis test.

We also quantified production of further key innate chemokines and cytokines in our system. RV-A1 infection increased CXCL10 and CCL5 protein levels at 96 hpi, with no difference between groups (Figure 3A). IL-8 was significantly induced following RV-A1 infection of cultures isolated from asthma donors, but no significant increase was observed in healthy or COPD groups (Figure 3A). Interleukin 6 (IL-6) protein release was not significantly increased following RV-A1 infection, and no difference was observed between disease groups (Figure 3B). No change in MUC5AC protein levels were observed following RV-A1 infection in any group, assessed in culture supernatants (Figure 3B) and by immunohistochemistry staining (Figure 3C, Supplementary Figure 1A). Further, cellular mucin protein expression was not altered by RV-A1 infection, as assessed by Alcian Blue (AB) staining on earlier timepoints (0 and 48 hpi; Supplementary Figures 1B–E).

### Delayed Genomic Responses to RV Infection in Epithelial Cells From Asthma and COPD Donors

**Figure 4:**
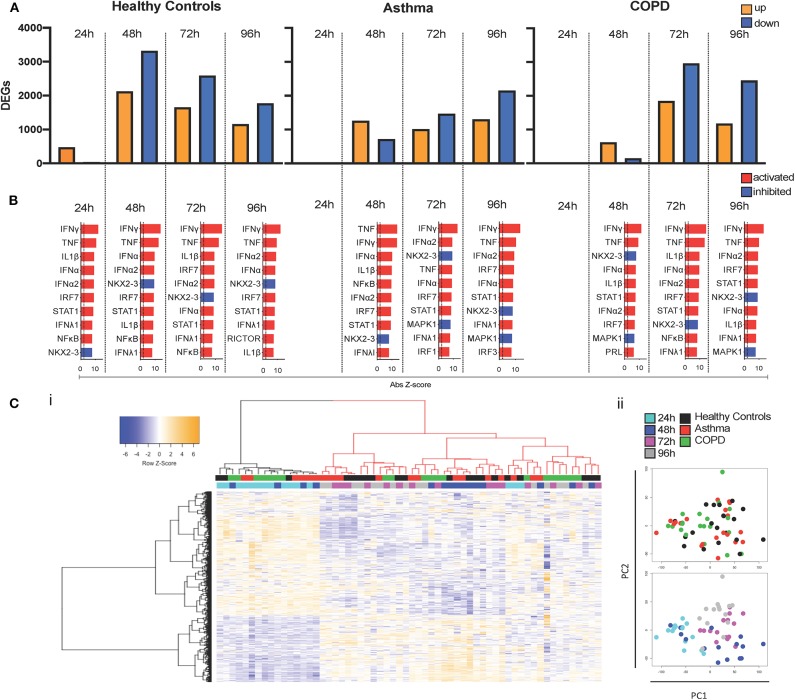
Delayed induction of DEGs and molecular drivers. DEGs (FDR < 0.05; Fold change > 1.5) were identified at each timepoint following RV-A1 infection of healthy donor cultures and cultures from asthma or COPD donors **(A)**. Data analysis was performed using EdgeR. Molecular drivers of gene expression changes were inferred using Upstream Regulator Analysis in healthy donor cultures **(B)**. The top ten drivers presented are ranked by activation Z score. The dashed vertical line indicates FDR < 0.05. Red bars indicate pathway activation (activation Z-score > 2.0) and blue bars indicate pathway inhibition (activation Z-score < −2.0). **(C)** Heatmap (i) and PCA (ii) of DEGs across the time course of infection. Virus infected samples were normalized to their respective mock infected controls at each timepoint.

We next sought to broadly characterize global changes in gene expression using RNA-seq. In cultures from healthy donors, RV-A1 infection altered 535 differentially expressed genes (DEGs; 474 up-regulated, 61 down-regulated) at 24 hpi, which peaked at 48 hpi with 5,447 DEGs (Figure 4A). Numbers of DEGs then decreased at 72 hpi (4,247 DEGs) and 96 hpi (2,932 DEGs). In cultures from asthma and COPD donors, the early response was absent at 24 hpi, with no DEGs detected in COPD and three upregulated genes identified in asthma. In infected cultures from asthma donors, total numbers of DEGs increased to 1,960 at 48 hpi, 2,477 at 72 hpi, and 3,455 at 96 hpi. In cultures from COPD donors, 779 DEGs were detected at 48 hpi, 4,810 at 72 hpi, and 3,636 at 96 hpi (Figure 4A). A plot of the total DEG numbers up-regulated (Supplementary Figure 2A) and down-regulated (Supplementary Figure 2B) at each timepoint clearly outlines a delay in gene induction following RV-A1 infection in cultures from patients with pre-existing lung disease and a more rapid resolution of induced innate immune response in cultures from healthy control donors.

To gain insight into the molecular pathways driving gene expression changes following RV-A1 infection, upstream regulator analysis (URA) was performed (46). URA leverages existing experimental evidence to identify candidate molecular pathways predicted to drive differential gene expression responses. Pathways were ranked by activation Z-score (Figure 4B) and overlap *p*-value (Supplementary Figure 3). Responses in healthy BECs at 24 hpi were primarily driven by induction of type I, II and III interferons (IFN-α/β, IFN-γ, IFN-λ) and pro-inflammatory cytokines (TNF and IL1β), which persisted through later timepoints (Figure 4B; Supplementary Figure 3A). No pathways were identified at 24 hpi in either asthma or COPD donor cultures, as gene expression was not induced at this timepoint (Figure 4B; Supplementary Figures 3B,C). Putative molecular drivers at 48 hpi and later timepoints in the asthma and COPD groups were similar to healthy (namely induction of interferons and pro-inflammatory cytokines; Figure 4B; Supplementary Figures 3B,C). Thus, the primary alteration of innate immunity in asthma and COPD is a delay in activation.

We employed hierarchical cluster analysis to group all DEGs (and samples) based on their expression patterns over time (virus infected samples were normalized to their respective mock infected controls at each timepoint) and visualized the data as a heatmap. The samples were partitioned into two groups (Figure 4Ci). Notably, samples that clustered on the left-hand side of the dendrogram (Figure 4Ci black colored dendrogram) were characterized by a relatively low response intensity, and this cluster included 24 h samples for all three groups, and 48 h samples from subjects with asthma or COPD. This was consistent with the limited number of DEGs identified in controls and 24 h, and the delayed response observed in subjects with asthma and COPD. Principal component analysis demonstrates that the samples are clustering largely on timepoint post-infection, and a number of 48 h samples from both disease groups are clustering with 24 h samples Figure 4Cii.

### Network Analysis Identifies Delayed Induction of Innate/Anti-viral Co-expression Modules in Asthma and COPD

**Figure 5:**
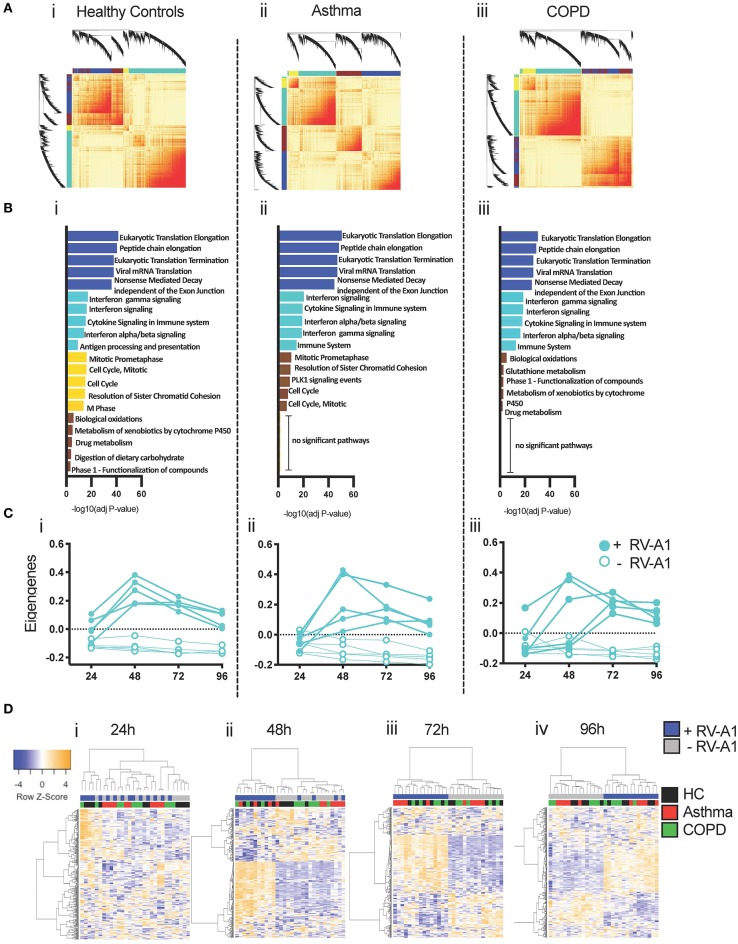
Co-expression networks underlying RV-A1-driven BEC responses. **(A)** Heatmap illustrating the gene co-expression networks detected following RV-A1 infection of healthy donor cultures. Each gene is represented by a branch on the dendrogram. Increasing red intensity indicates increasing strength of correlation between genes and modules correspond to blocks of highly correlated genes. **(B)** Top five biological pathways enriched in each WGCNA module following RV-A1 infection. Data analyzed using InnateDB. **(C)** Dynamics of the innate turquoise module eigengene in mock (open circles) and RV-A1 infected cells (closed circles) at the indicated timepoints. **(D)** Heatmap of the 300 most variable genes within the turquoise module in healthy controls, asthma and COPD. Each row corresponds to a gene, and each column to a sample.

Genes and proteins do not function in isolation, but rather are organized into functional modules (47). To characterize the modular organization of the RV-A1-induced immune response, we performed weighted gene co-expression network analysis (WGCNA) (44, 48). Separate co-expression networks were constructed for each clinical group. Resulting networks were organized into four modules (Figure 5A). Functional enrichment analysis indicated that individual modules in each group were functionally distinct (Figure 5B). In all groups, an innate anti-viral module was identified (turquoise module), which was enriched with genes that mediate interferon-induced antiviral responses and interleukin signaling (Figure 5B). The turquoise module contained 3,033 genes in healthy controls, 2,000 genes in asthma and 2,515 genes in COPD. A core set of 1,513 overlapping genes were common between all three clinical groups (Supplementary Table 2).

To examine expression patterns, we summarized the overall expression of the turquoise module using principal components analysis, and plotted the module eigengene (ME, i.e., first principal component) for each sample (Supplementary Figure 4). The turquoise module was upregulated at 24 hpi in cultures from healthy donors. Upregulated genes included archetypal interferon-induced anti-viral pathways (e.g., IFNλ, IFNβ, IRF7, IRF9, ISG15, Mx1, STAT1). In contrast, the turquoise module was not activated at 24 hpi in either asthma or COPD cultures (Supplementary Figure 4). In both disease groups, activation of the turquoise module over the time-course of infection was more variable between individual patients, compared to cultures from healthy donors (Figure 5C). To visualize subject-to-subject variations in gene expression patterns of the core genes in the turquoise module across the three patient groups, we employed hierarchical cluster analysis and plotted the data at each timepoint as a heatmap (Figure 5D). At 24 h, there is a distinct cluster of samples with activated gene expression patterns. This cluster contains 4 out of 5 virus-infected healthy controls samples and one COPD sample (Figure 5Di). At 48 h, there are two clusters; the first contains all healthy control viral samples and three viral samples from each disease groups; and the second containing all mock infected samples across all groups in addition to two viral samples from each disease group (Figure 5Dii). At 72 and 96 h samples are clustering by virus infection (Figures 5Diii,iv).

We next compared gene network patterns of the antiviral response module. Expression and connectivity ranks were calculated for each gene and correlated in cases vs. controls. Gene expression network patterns were highly correlated in asthma and COPD (Supplementary Figures 5Ai,Bi), but gene connectivity patterns were more variable (Supplementary Figures 5Aii,Bii). Thus, overall gene expression patterns were conserved between healthy controls and subjects with airways disease, but conservation of the connectivity patterns was weaker.

## Discussion

Our study shows that *in vitro* ALI-differentiated BECs obtained from healthy donors and donors with respiratory Disease can be infected with RV at low MOI (orders of magnitude lower than MOI 1, which is typically used in studies). The utility of a low MOI RV infection model in differentiated human primary BECs to investigate viral replication and anti-viral immunity was recently reported (49). We have extended this to apply the model with genome-wide analyses to BECs from healthy subjects as well as donors with chronic respiratory disease. We observed that ALI BEC cultures generated from donors with asthma or COPD had delayed innate anti-viral immune responses compared to BEC cultures derived from health donors. However, once responses initiate, cultures from donors with asthma or COPD elicit robust anti-viral response networks, which do not differ from healthy controls. This is consistent with some previous findings using submerged BEC culture to assess responses to RV infection (19, 24). We provide a detailed kinetic assessment showing delayed IFN activation and ISG expression following RV infection of BECs from asthma and COPD donors, supported by systems-level analyses of RNA-Seq profiles.

We noted no significant differences in viral levels between disease groups, indicating that viral infection, replication and release from epithelial cells was not significantly altered by the disease status and associated delayed BEC immune response. This contrasts with previous data in monolayer cultures, which reported increased viral replication in cells isolated from donors with respiratory disease following high MOI RV infection (19, 23, 24, 30). For example, infection of monolayer cultures from asthma donors with RV-A16 (MOI = 2) had increased viral replication, compared with healthy donors, with an associated decrease in IFN responses (24). Increased viral replication has also been associated with deficient IFN-λ induction in cultures from asthma donors (19). In contrast, our findings indicate that despite equivalent levels of viral replication, BECs from asthma and COPD respond slower than cells from healthy donors.

RV-A1 infection of healthy donor epithelial cell cultures resulted in a rapid and robust induction of IFN responses. In cultures from asthma and COPD donors, *IFN-*β and *IFN-*λ gene expression were not induced at early timepoints, compared to healthy donors. However, at 72–96 hpi, expression levels were restored to similar levels across all disease groups (other than the reduced total IFN-λ protein levels observed in asthma cultures at 96 hpi). Similarly, ISG expression were not induced at 24 hpi in disease groups but were restored to healthy levels at later timepoints. These findings reveal an early delay in interferon activation, rather than an overall deficiency in cells isolated from asthma and COPD donors. These observations may explain discrepancies in the literature, where some studies have reported IFN deficiency following RV infection in cells from asthma or COPD donors and others have failed to identify a difference (26–28). Additional factors that may contribute to differences include specific disease characteristics (e.g., disease severity, treatment status, inflammatory phenotype) and culture conditions (e.g., ALI vs. monolayer, virus MOI, timepoints assessed). To date, there is limited data on IFN responses in ALI cultures following RV infection. Lopez-Souza et al. reported no difference in viral replication in fully differentiated cells from asthma or healthy donors, but identified an increased *IFN-*β*1* gene expression in asthma donors compared to healthy (26). It is interesting to note that despite our observation of delayed interferon activation in asthma and COPD cultures, there was no difference in viral loads. We speculate that *in vivo* delayed innate activation in airway epithelial cells may have broader downstream impacts, including delayed innate immune cell recruitment and activation. *In vivo*, this may contribute to increased viral replication and/or dissemination and worsened pathology (50–52).

Our RNA-Seq data provide the first detailed kinetic assessment of RV-A1 infection effects over a 4-days time course in healthy, asthma and COPD cultures. One previous study reported a RNA sequencing approach in ALI culture samples from healthy and asthma donors, although only at a single timepoint after RV-A16 infection (i.e., 24 hpi), where expression of 1,485 genes was altered following RV infection (pooled asthma and healthy). Gene pathway analysis identified modulation of IFN responses (Type I, Type II), innate immune responses, type-2 immune gene signatures, apoptosis, regulation of viral reproduction, viral RNA synthesis, adherence, cilia movement and cilia morphogenesis (the last 4 being downregulated). Cultures from asthma donors had higher CXCL10 and CCL5 gene expression (along with a further 44 genes) after RV infection, compared to healthy donors (53).

In contrast, we demonstrate a delayed IFN response in asthmatic and COPD cells following low MOI RV-A1 infection. Most of the upregulated genes were responsible for IFN signaling and innate immune responses at early timepoints in cultures from healthy donors. In contrast cells from asthma and COPD donors did not induce gene expression in response to RV infection at 24 hpi. Rather, gene expression was not altered until 48 hpi in disease groups, at which point all groups had similar DEG profiles in response to infection. At this timepoint a lower number of DEGs were identified in cultures from asthma and COPD donors, compared to healthy donors. In healthy cells, gene expression declined rapidly after the peak indicating controlled infection with robust early IFN responses. In contrast, in cultures from asthma and COPD donors, the number of DEGs increased over time and peaked later (96 and 72 hpi, respectively), indicating delayed innate immunity. This suggests susceptibility to RV infection in subjects with chronic airways disease may stem from the delayed timing and prolonged duration of the host immune response (heatmap and gene network pattern, Figure 5). In this context, it is noteworthy that upper airway responses in children who present to hospital with RV-induced wheezing have been characterized into IRF7hi and IRF7lo molecular phenotypes, based on differing timing from first symptoms to hospital presentation (54).

Our time course analysis provides new insight into epithelial innate immune deficiencies that have been reported, particularly in asthma. By assessing multiple timepoints, we can conclude that the deficiency stems from a delayed early response, which contributes downstream effects on of innate immune response kinetics. The specific mechanism underlying this delayed response remains unclear. Delayed IFN production could result from reduced antigen recognition, as has been reported in samples from children with severe asthma where reduced TLR3, RIG, and MDA5 expression were observed (30). The delayed responses may result from ongoing lung inflammation present in asthma or COPD. Innate immune responses at mucosal surfaces are tightly regulated to suppress inappropriate inflammation, with the production of suppressive cytokines required to maintain tissue homeostasis (55). An “innate immune rheostat” model has been proposed, recognizing that innate immunity is adaptable and responsive to the local environment, which in turn modulates responses to infection (56). Thus, ongoing inflammation may lead to desensitization and result in delayed innate immune responses. This model has largely been explored in the context of innate immune cells (e.g., macrophages) or interactions between immune cells and epithelial cells. Similarly, studies that identified impaired IFN responses in asthma or COPD have largely focussed on production by immune cells (e.g., peripheral blood mononuclear cells) (57). Here we demonstrate that delayed IFN induction occurs in the absence of associated immune cells, in an epithelial/structural cell-intrinsic manner. However, the downstream effects of this delay on resident or infiltrating immune cells remains to be explored. The mechanism may also be modulated by disease phenotype, as the presence of type 2 cytokines in allergic disease inhibit IFN production [potentially via increased suppressor of cytokine signaling-1 (SOCS1) expression] (58). Regardless of the mechanism, our data indicate that approaches to accelerate epithelial innate immune responses following RV infection may be beneficial for people with existing respiratory disease. The timing of such interventions will be critical with prophylactic innate immune priming likely more feasible than post-infection intervention.

We acknowledge several limitations of the current study. Our sample size was small (5 donors per group), this was somewhat necessary to enable a comprehensive time course analysis which allowed us to define the timing-related primary outcome of this work. While ALI cultures more accurately represent airway epithelium than monolayer cultures, they do not contain associated tissues types, including immune cells. Further, our assessment was limited to a single RV subtype (RV-A1). Further studies are necessary to extend findings to other viruses and provide further understanding of disease-relevant differences.

In the current study, we describe a novel low MOI infection model, using differentiated ALI epithelial cultures derived from healthy, asthma and COPD donors. Our findings demonstrate that low MOI exposure to RV-A1 is sufficient to support viral replication. Infection led to a rapid activation of innate immune responses in healthy control samples. A detailed assessment of innate immune kinetics revealed delayed innate immune activation in differentiated epithelial cultures from asthma and COPD donors. We propose that this delay may contribute to impaired anti-viral defense in people with respiratory disease and contribute to the severe symptoms observed during viral-induced exacerbations. Approaches that can correct this delay may provide new therapeutic options to treat both RV infection and disease exacerbations.

## Data Availability Statement

The RNA sequencing data has been uploaded to the GEO-GSE146532. Other raw data supporting the conclusions of this article will be made available by the authors, without undue reservation, to any qualified researcher.

## Ethics Statement

All experiments were conducted in accordance with the Hunter New England Area Health Service Ethics Committee and the University of Newcastle Safety Committee (05/08/10/3.09). Primary BECs were obtained via bronchoscopy from healthy, asthma and COPD subjects following written informed consent.

## Author Contributions

PV, DK, CG, and NB: conceptualization and methodology. CG, PW, and KN: resources. PV: experimentation and writing—initial draft. PV, AR, and NL: investigations. NT, PK, and AB: RNA seq analysis. DK, CG, and NB: supervision. CG and NB: project administration. PV, NT, AR, SM, PW, DK, AB, CG, and NB: writing—review and editing.

## Conflict of Interest

NB has received researcher-initiated funding and consulting fees from Ena Therapeutics who are developing TLR agonists for respiratory virus infections. The remaining authors declare that the research was conducted in the absence of any commercial or financial relationships that could be construed as a potential conflict of interest.
