# Supplementary material for: Airway Epithelial Cell Immunity Is Delayed During Rhinovirus Infection in Asthma and COPD
Source: Front Immunol. 2020 May 15;11:974. doi: 10.3389/fimmu.2020.00974 (PMC7243842; doi:10.3389/fimmu.2020.00974)
Supplement: Supplementary file 1 [file Table_1.DOCX]

**Airway epithelial cell immunity is delayed during rhinovirus infection in asthma and COPD**

**Punnam Chander Veerati^1,2†^, Niamh M. Troy^3†^, Andrew T. Reid^1,2^, Ngan Fung Li^2,4^, Kristy S. Nichol^1,2^, Parwinder Kaur^5^, Steven Maltby^2,4^, Peter A.B. Wark^1,2,6^, Darryl A. Knight^2,4,7,8^, Anthony Bosco^3†^, Chris L. Grainge^1,2,6†^, Nathan W. Bartlett^2,4†^***

**^†^ *These authors contributed equally***

**Supplementary Materials:**

- Supplementary Figures 1 – 5
- Supplementary Tables 1 & 2

**Supplementary Figures**


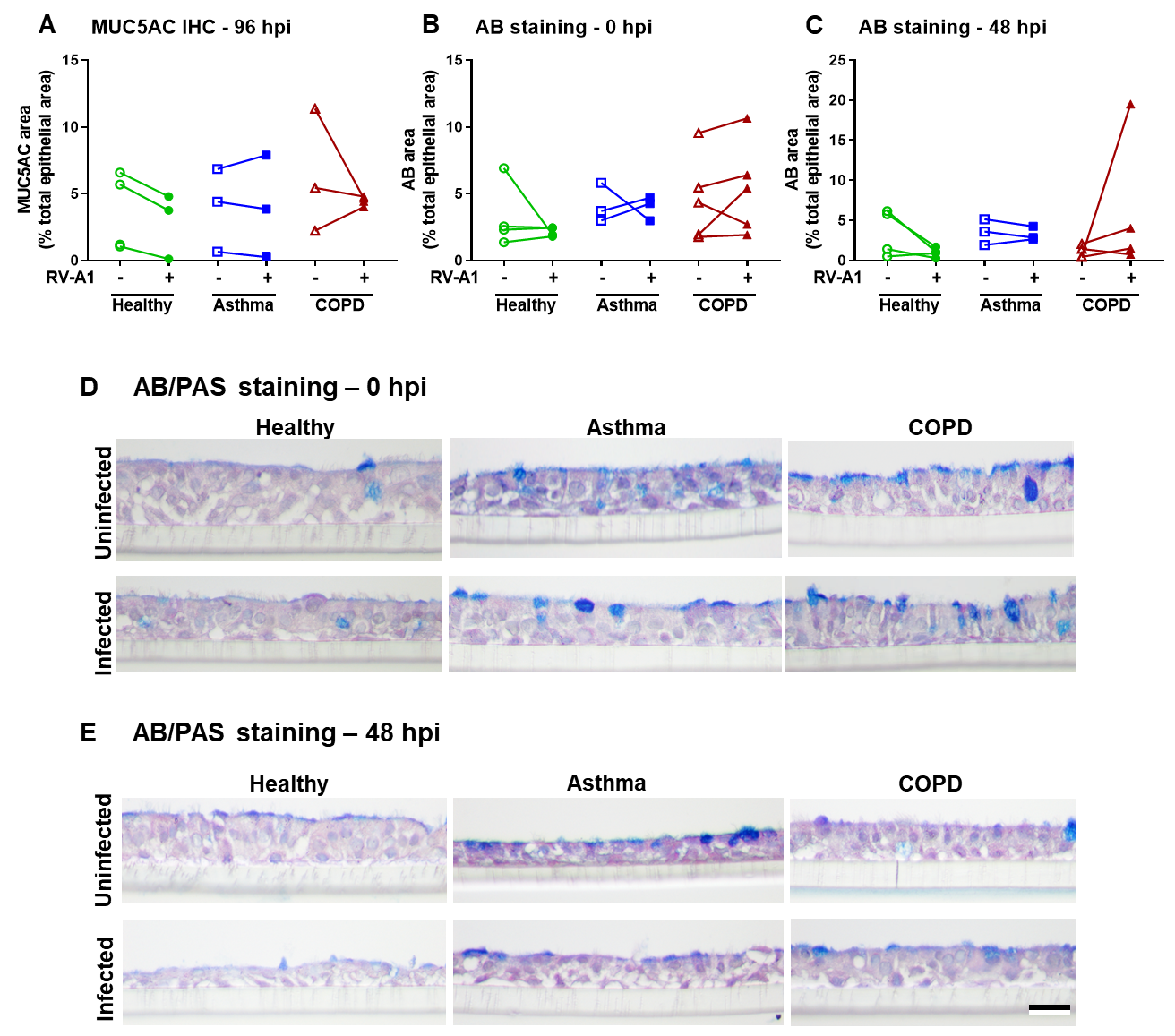


**Supplementary Figure 1: No change in BEC MUC5AC and mucin expression following RV-A1 infection.** ALI culture membranes at 0, 48 and 96 hpi were paraffin-embedded and subjected to AB/PAS staining and 96 hpi were stained with anti-MUC5AC antibody for immunohistochemistry assessment. Percentage of anti-MUC5AC staining per total BEC area was platted for 96 hpi (A) and AB staining per total BEC area were plotted for 0 hpi (B) and 48 hpi (C). Representative images of AB/PAS staining at 0 hpi (D) and 48 hpi (E) for all groups. Biological replicates represented as individual points and analysed using Wilcoxon signed-rank test within the group and across the infected groups by Kruskal-Wallis test (n=3-5). Scale bar: 25µm.

**Supplementary Figure 2: Time course of DEG expression following RV-A1 infection.** Upregulated (A) and downregulated (B) genes with Fold change (FC) > 1.5 and False Discovery Rate (FDR) < 0.05 at 24, 48, 72 and 96 hpi in BEC cultures from healthy, asthma and COPD donors. Data analysis was performed using EdgeR.

**
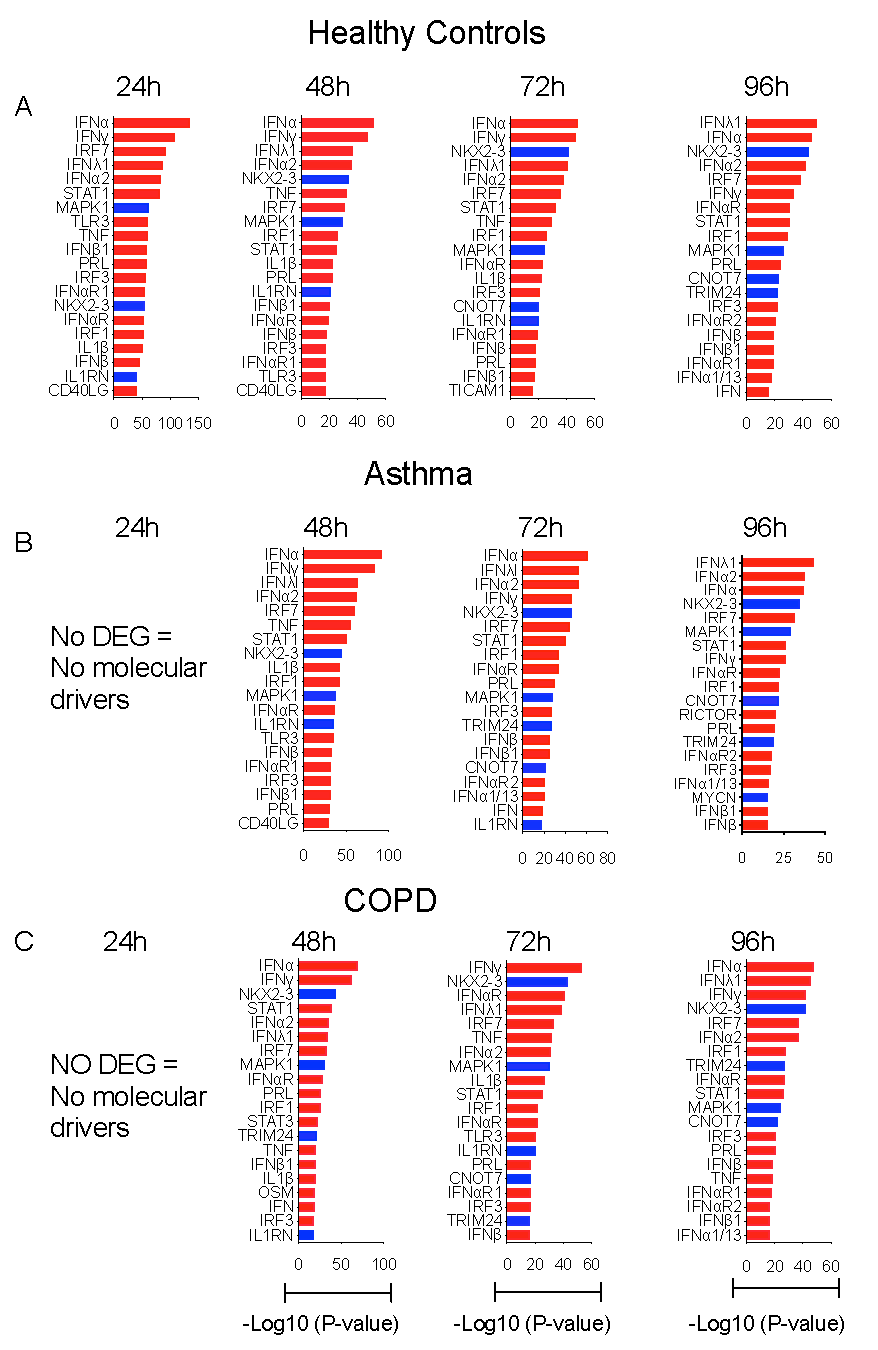
**

**Supplementary Figure 3: Top 20 significant molecular drivers of gene expression changes at each timepoint.** Molecular drivers of gene expression changes referred using Upstream Regulator Analysis in healthy (**A** i-iv), asthma (**B** i-iv) and COPD (**C** i-iv) donor bronchial epithelial cells, ranked by P-value.

**
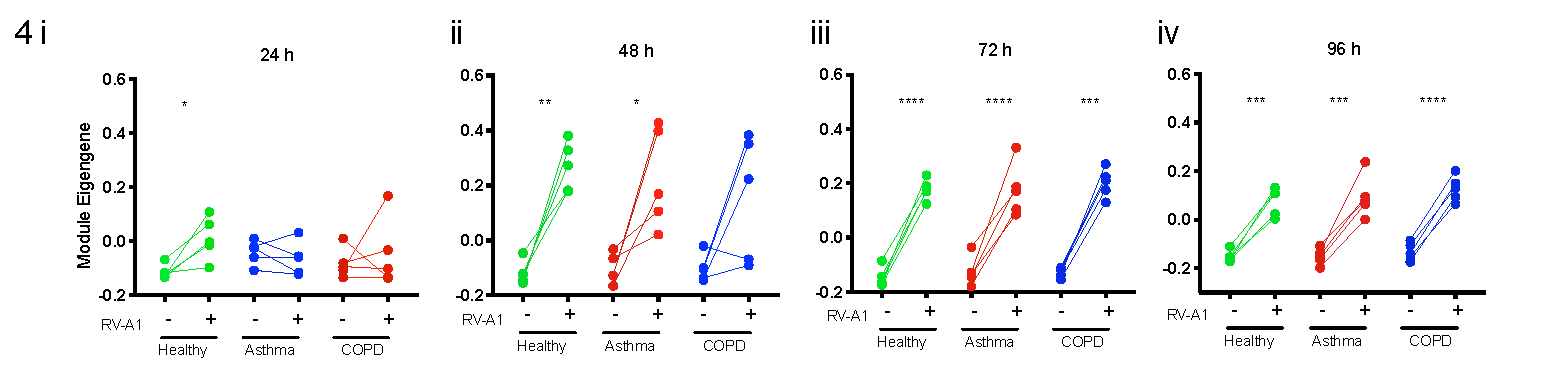
**

**Supplementary Figure 4: Delayed induction of turquoise innate module eigengene.** Module expression/eigengene in mock and RV-A1 infected cells at 24 (i), 48 (ii) 72 (iii) and 96 hours (iv). *FDR<0.05, ** FDR<0.005, ***FDR<0.0005.

**Supplementary Figure 5: Correlation of healthy Control “turquoise” module genes and module genes identified in asthma (A)/COPD (B).** Gene expression correlations A/B(i) and gene connectivity correlations A/B(ii). Cor value and P-value are calculated using WGCNA package.

**Supplementary Tables**

**Supplementary Table 1: qPCR primer and probe sequences**

| **Gene** | **Sequence (5'-3')** |
| --- | --- |
| Rhinovirus | Forward : 5'-GTGAAGAGCCsCrTGTGCT-3'  Reverse: 5'-GCTsCAGGGTTAAGGTTAGCC-3'  Probe: 5'-FAM-TGAGTCCTCCGGCCCCTGAATG-TAMRA-3' |
| 18S | Forward : 5'-CGCCGCTAGAGGTGAAATTCT-3'  Reverse: 5'-CATTCTTGGCAAATGCTTTCG-3'  Probe: 5'-FAM-ACCGGCGCAAGACGGACCAGA-TAMRA-3' |
| IFN-β | Forward : 5'-CGCCGCATTGACCATCTA-3'  Reverse: 5'-TTAGCCAGGAGGTTCTCAACAATAGTCTCA-3'  Probe: 5'-FAM-TCAGACAAGATTCATCTAGCACTGGCTGGA-TAMRA-3' |
| IFN-λ 2/3 | Forward : 5'-CTGCCACATAGCCCAGTTCA-3'  Reverse: 5'-AGAAGCGACTCTTCTAAGGCATCTT-3'  Probe: 5'-FAM-TCTCCACAGGAGCTGCAGGCCTTTA-TAMRA-3' |
| OAS1 | Forward : 5'-CTGACFCTGACCTGGTTGTCT-3'  Reverse: 5'-CCCCGGCGATTTAACTGAT-3'  Probe: 5'-FAM-CCTCAGTCCTCTCACCACTTTTCA-TAMRA-3' |
| PKR | Forward : 5'-AAGGGAACTTTGCGATACATGAG-3'  Reverse: 5'-GCGTAGAGGTCCACTTCCTTTC-3'  Probe: 5'-FAM-CCAGAACAGATTTCTTCGCAAGACTAT-TAMRA-3' |
| Viperin | Forward : 5'-CACAAAGAAGTGTCCTGCTTGGT-3'  Reverse: 5'-AAGCGCATATATTTCATCCAGAATAAG-3'  Probe: 5'-FAM-CCTGAATCTAACCAGAAGATGAAAGACTCC-TAMRA-3' |

**Supplementary Table 2: Core turquoise module genes present in healthy controls, asthma and COPD**

| **Gene Name** | **EnsemblID** | **Description** |
| --- | --- | --- |
| CFH | ENSG00000000971 | complement factor H [Source:HGNC Symbol;Acc:4883] |
| ANKIB1 | ENSG00000001629 | ankyrin repeat and IBR domain containing 1 [Source:HGNC Symbol;Acc:22215] |
| LAP3 | ENSG00000002549 | leucine aminopeptidase 3 [Source:HGNC Symbol;Acc:18449] |
| HS3ST1 | ENSG00000002587 | heparan sulfate (glucosamine) 3-O-sulfotransferase 1 [Source:HGNC Symbol;Acc:5194] |
| CASP10 | ENSG00000003400 | caspase 10, apoptosis-related cysteine peptidase [Source:HGNC Symbol;Acc:1500] |
| CFLAR | ENSG00000003402 | CASP8 and FADD-like apoptosis regulator [Source:HGNC Symbol;Acc:1876] |
| CD38 | ENSG00000004468 | CD38 molecule [Source:HGNC Symbol;Acc:1667] |
| CCDC109B | ENSG00000005059 | coiled-coil domain containing 109B [Source:HGNC Symbol;Acc:26076] |
| PRKAR2B | ENSG00000005249 | protein kinase, cAMP-dependent, regulatory, type II, beta [Source:HGNC Symbol;Acc:9392] |
| MYCBP2 | ENSG00000005810 | MYC binding protein 2, E3 ubiquitin protein ligase [Source:HGNC Symbol;Acc:23386] |
| CX3CL1 | ENSG00000006210 | chemokine (C-X3-C motif) ligand 1 [Source:HGNC Symbol;Acc:10647] |
| SCIN | ENSG00000006747 | scinderin [Source:HGNC Symbol;Acc:21695] |
| PRSS21 | ENSG00000007038 | protease, serine, 21 (testisin) [Source:HGNC Symbol;Acc:9485] |
| NOS2 | ENSG00000007171 | nitric oxide synthase 2, inducible [Source:HGNC Symbol;Acc:7873] |
| NADK | ENSG00000008130 | NAD kinase [Source:HGNC Symbol;Acc:29831] |
| IL32 | ENSG00000008517 | interleukin 32 [Source:HGNC Symbol;Acc:16830] |
| MAPK8IP2 | ENSG00000008735 | mitogen-activated protein kinase 8 interacting protein 2 [Source:HGNC Symbol;Acc:6883] |
| TRAF3IP3 | ENSG00000009790 | TRAF3 interacting protein 3 [Source:HGNC Symbol;Acc:30766] |
| ETV7 | ENSG00000010030 | ets variant 7 [Source:HGNC Symbol;Acc:18160] |
| PLAUR | ENSG00000011422 | plasminogen activator, urokinase receptor [Source:HGNC Symbol;Acc:9053] |
| SEMA3B | ENSG00000012171 | sema domain, immunoglobulin domain (Ig), short basic domain, secreted, (semaphorin) 3B [Source:HGNC Symbol;Acc:10724] |
| MAN2B2 | ENSG00000013288 | mannosidase, alpha, class 2B, member 2 [Source:HGNC Symbol;Acc:29623] |
| NUB1 | ENSG00000013374 | negative regulator of ubiquitin-like proteins 1 [Source:HGNC Symbol;Acc:17623] |
| POLA2 | ENSG00000014138 | polymerase (DNA directed), alpha 2, accessory subunit [Source:HGNC Symbol;Acc:30073] |
| ACPP | ENSG00000014257 | acid phosphatase, prostate [Source:HGNC Symbol;Acc:125] |
| MTMR11 | ENSG00000014914 | myotubularin related protein 11 [Source:HGNC Symbol;Acc:24307] |
| CLCA4 | ENSG00000016602 | chloride channel accessory 4 [Source:HGNC Symbol;Acc:2018] |
| CD74 | ENSG00000019582 | CD74 molecule, major histocompatibility complex, class II invariant chain [Source:HGNC Symbol;Acc:1697] |
| SERPINB1 | ENSG00000021355 | serpin peptidase inhibitor, clade B (ovalbumin), member 1 [Source:HGNC Symbol;Acc:3311] |
| GRAMD1B | ENSG00000023171 | GRAM domain containing 1B [Source:HGNC Symbol;Acc:29214] |
| BIRC3 | ENSG00000023445 | baculoviral IAP repeat containing 3 [Source:HGNC Symbol;Acc:591] |
| TYMP | ENSG00000025708 | thymidine phosphorylase [Source:HGNC Symbol;Acc:3148] |
| FAS | ENSG00000026103 | Fas cell surface death receptor [Source:HGNC Symbol;Acc:11920] |
| SLAMF7 | ENSG00000026751 | SLAM family member 7 [Source:HGNC Symbol;Acc:21394] |
| BTN3A1 | ENSG00000026950 | butyrophilin, subfamily 3, member A1 [Source:HGNC Symbol;Acc:1138] |
| IFNGR1 | ENSG00000027697 | interferon gamma receptor 1 [Source:HGNC Symbol;Acc:5439] |
| TNFRSF1B | ENSG00000028137 | tumor necrosis factor receptor superfamily, member 1B [Source:HGNC Symbol;Acc:11917] |
| ARNTL2 | ENSG00000029153 | aryl hydrocarbon receptor nuclear translocator-like 2 [Source:HGNC Symbol;Acc:18984] |
| BAK1 | ENSG00000030110 | BCL2-antagonist/killer 1 [Source:HGNC Symbol;Acc:949] |
| GRN | ENSG00000030582 | granulin [Source:HGNC Symbol;Acc:4601] |
| ARHGAP31 | ENSG00000031081 | Rho GTPase activating protein 31 [Source:HGNC Symbol;Acc:29216] |
| TIMP2 | ENSG00000035862 | TIMP metallopeptidase inhibitor 2 [Source:HGNC Symbol;Acc:11821] |
| MYOM2 | ENSG00000036448 | myomesin 2 [Source:HGNC Symbol;Acc:7614] |
| AGA | ENSG00000038002 | aspartylglucosaminidase [Source:HGNC Symbol;Acc:318] |
| PI4K2B | ENSG00000038210 | phosphatidylinositol 4-kinase type 2 beta [Source:HGNC Symbol;Acc:18215] |
| PHLPP2 | ENSG00000040199 | PH domain and leucine rich repeat protein phosphatase 2 [Source:HGNC Symbol;Acc:29149] |
| RAB27B | ENSG00000041353 | RAB27B, member RAS oncogene family [Source:HGNC Symbol;Acc:9767] |
| PSMA4 | ENSG00000041357 | proteasome (prosome, macropain) subunit, alpha type, 4 [Source:HGNC Symbol;Acc:9533] |
| PARP3 | ENSG00000041880 | poly (ADP-ribose) polymerase family, member 3 [Source:HGNC Symbol;Acc:273] |
| TNC | ENSG00000041982 | tenascin C [Source:HGNC Symbol;Acc:5318] |
| FAM65C | ENSG00000042062 | family with sequence similarity 65, member C [Source:HGNC Symbol;Acc:16168] |
| CAPG | ENSG00000042493 | capping protein (actin filament), gelsolin-like [Source:HGNC Symbol;Acc:1474] |
| ADAM28 | ENSG00000042980 | ADAM metallopeptidase domain 28 [Source:HGNC Symbol;Acc:206] |
| JADE2 | ENSG00000043143 | jade family PHD finger 2 [Source:HGNC Symbol;Acc:22984] |
| ARAP2 | ENSG00000047365 | ArfGAP with RhoGAP domain, ankyrin repeat and PH domain 2 [Source:HGNC Symbol;Acc:16924] |
| CP | ENSG00000047457 | ceruloplasmin (ferroxidase) [Source:HGNC Symbol;Acc:2295] |
| USP28 | ENSG00000048028 | ubiquitin specific peptidase 28 [Source:HGNC Symbol;Acc:12625] |
| HDAC9 | ENSG00000048052 | histone deacetylase 9 [Source:HGNC Symbol;Acc:14065] |
| COL9A2 | ENSG00000049089 | collagen, type IX, alpha 2 [Source:HGNC Symbol;Acc:2218] |
| NFE2L3 | ENSG00000050344 | nuclear factor, erythroid 2-like 3 [Source:HGNC Symbol;Acc:7783] |
| TNIP3 | ENSG00000050730 | TNFAIP3 interacting protein 3 [Source:HGNC Symbol;Acc:19315] |
| POLQ | ENSG00000051341 | polymerase (DNA directed), theta [Source:HGNC Symbol;Acc:9186] |
| CYBA | ENSG00000051523 | cytochrome b-245, alpha polypeptide [Source:HGNC Symbol;Acc:2577] |
| MSMO1 | ENSG00000052802 | methylsterol monooxygenase 1 [Source:HGNC Symbol;Acc:10545] |
| NRIP2 | ENSG00000053702 | nuclear receptor interacting protein 2 [Source:HGNC Symbol;Acc:23078] |
| LAMA3 | ENSG00000053747 | laminin, alpha 3 [Source:HGNC Symbol;Acc:6483] |
| PLEKHH1 | ENSG00000054690 | pleckstrin homology domain containing, family H (with MyTH4 domain) member 1 [Source:HGNC Symbol;Acc:17733] |
| EIF2AK2 | ENSG00000055332 | eukaryotic translation initiation factor 2-alpha kinase 2 [Source:HGNC Symbol;Acc:9437] |
| SERPINB3 | ENSG00000057149 | serpin peptidase inhibitor, clade B (ovalbumin), member 3 [Source:HGNC Symbol;Acc:10569] |
| LAMC2 | ENSG00000058085 | laminin, gamma 2 [Source:HGNC Symbol;Acc:6493] |
| PARP12 | ENSG00000059378 | poly (ADP-ribose) polymerase family, member 12 [Source:HGNC Symbol;Acc:21919] |
| MXD1 | ENSG00000059728 | MAX dimerization protein 1 [Source:HGNC Symbol;Acc:6761] |
| STYK1 | ENSG00000060140 | serine/threonine/tyrosine kinase 1 [Source:HGNC Symbol;Acc:18889] |
| OGFR | ENSG00000060491 | opioid growth factor receptor [Source:HGNC Symbol;Acc:15768] |
| GUCY1B3 | ENSG00000061918 | guanylate cyclase 1, soluble, beta 3 [Source:HGNC Symbol;Acc:4687] |
| CDH3 | ENSG00000062038 | cadherin 3, type 1, P-cadherin (placental) [Source:HGNC Symbol;Acc:1762] |
| ELMO2 | ENSG00000062598 | engulfment and cell motility 2 [Source:HGNC Symbol;Acc:17233] |
| NGFR | ENSG00000064300 | nerve growth factor receptor [Source:HGNC Symbol;Acc:7809] |
| HIPK2 | ENSG00000064393 | homeodomain interacting protein kinase 2 [Source:HGNC Symbol;Acc:14402] |
| CHI3L2 | ENSG00000064886 | chitinase 3-like 2 [Source:HGNC Symbol;Acc:1933] |
| SBNO2 | ENSG00000064932 | strawberry notch homolog 2 (Drosophila) [Source:HGNC Symbol;Acc:29158] |
| CALCRL | ENSG00000064989 | calcitonin receptor-like [Source:HGNC Symbol;Acc:16709] |
| UHRF1BP1 | ENSG00000065060 | UHRF1 binding protein 1 [Source:HGNC Symbol;Acc:21216] |
| COL17A1 | ENSG00000065618 | collagen, type XVII, alpha 1 [Source:HGNC Symbol;Acc:2194] |
| IDI1 | ENSG00000067064 | isopentenyl-diphosphate delta isomerase 1 [Source:HGNC Symbol;Acc:5387] |
| SP100 | ENSG00000067066 | SP100 nuclear antigen [Source:HGNC Symbol;Acc:11206] |
| STOML1 | ENSG00000067221 | stomatin (EPB72)-like 1 [Source:HGNC Symbol;Acc:14560] |
| IFI35 | ENSG00000068079 | interferon-induced protein 35 [Source:HGNC Symbol;Acc:5399] |
| BCL3 | ENSG00000069399 | B-cell CLL/lymphoma 3 [Source:HGNC Symbol;Acc:998] |
| DAPP1 | ENSG00000070190 | dual adaptor of phosphotyrosine and 3-phosphoinositides [Source:HGNC Symbol;Acc:16500] |
| TRIB2 | ENSG00000071575 | tribbles pseudokinase 2 [Source:HGNC Symbol;Acc:30809] |
| ZFYVE26 | ENSG00000072121 | zinc finger, FYVE domain containing 26 [Source:HGNC Symbol;Acc:20761] |
| LNX1 | ENSG00000072201 | ligand of numb-protein X 1, E3 ubiquitin protein ligase [Source:HGNC Symbol;Acc:6657] |
| ASAH2C | ENSG00000072444 | N-acylsphingosine amidohydrolase (non-lysosomal ceramidase) 2C [Source:HGNC Symbol;Acc:23457] |
| SIDT1 | ENSG00000072858 | SID1 transmembrane family, member 1 [Source:HGNC Symbol;Acc:25967] |
| PANX2 | ENSG00000073150 | pannexin 2 [Source:HGNC Symbol;Acc:8600] |
| ALPK1 | ENSG00000073331 | alpha-kinase 1 [Source:HGNC Symbol;Acc:20917] |
| GSDMB | ENSG00000073605 | gasdermin B [Source:HGNC Symbol;Acc:23690] |
| ABCB11 | ENSG00000073734 | ATP-binding cassette, sub-family B (MDR/TAP), member 11 [Source:HGNC Symbol;Acc:42] |
| DHRS9 | ENSG00000073737 | dehydrogenase/reductase (SDR family) member 9 [Source:HGNC Symbol;Acc:16888] |
| PTGS2 | ENSG00000073756 | prostaglandin-endoperoxide synthase 2 (prostaglandin G/H synthase and cyclooxygenase) [Source:HGNC Symbol;Acc:9605] |
| MAP3K13 | ENSG00000073803 | mitogen-activated protein kinase kinase kinase 13 [Source:HGNC Symbol;Acc:6852] |
| TEAD2 | ENSG00000074219 | TEA domain family member 2 [Source:HGNC Symbol;Acc:11715] |
| ATP2A3 | ENSG00000074370 | ATPase, Ca++ transporting, ubiquitous [Source:HGNC Symbol;Acc:813] |
| CA12 | ENSG00000074410 | carbonic anhydrase XII [Source:HGNC Symbol;Acc:1371] |
| SEMA3A | ENSG00000075213 | sema domain, immunoglobulin domain (Ig), short basic domain, secreted, (semaphorin) 3A [Source:HGNC Symbol;Acc:10723] |
| TTC38 | ENSG00000075234 | tetratricopeptide repeat domain 38 [Source:HGNC Symbol;Acc:26082] |
| ATP12A | ENSG00000075673 | ATPase, H+/K+ transporting, nongastric, alpha polypeptide [Source:HGNC Symbol;Acc:13816] |
| BCAP29 | ENSG00000075790 | B-cell receptor-associated protein 29 [Source:HGNC Symbol;Acc:24131] |
| RBMS2 | ENSG00000076067 | RNA binding motif, single stranded interacting protein 2 [Source:HGNC Symbol;Acc:9909] |
| PLXNA2 | ENSG00000076356 | plexin A2 [Source:HGNC Symbol;Acc:9100] |
| GPC4 | ENSG00000076716 | glypican 4 [Source:HGNC Symbol;Acc:4452] |
| LRCH4 | ENSG00000077454 | leucine-rich repeats and calponin homology (CH) domain containing 4 [Source:HGNC Symbol;Acc:6691] |
| FBLN1 | ENSG00000077942 | fibulin 1 [Source:HGNC Symbol;Acc:3600] |
| MAP2 | ENSG00000078018 | microtubule-associated protein 2 [Source:HGNC Symbol;Acc:6839] |
| LAMP3 | ENSG00000078081 | lysosomal-associated membrane protein 3 [Source:HGNC Symbol;Acc:14582] |
| ADCY2 | ENSG00000078295 | adenylate cyclase 2 (brain) [Source:HGNC Symbol;Acc:233] |
| SLC1A3 | ENSG00000079215 | solute carrier family 1 (glial high affinity glutamate transporter), member 3 [Source:HGNC Symbol;Acc:10941] |
| SP140 | ENSG00000079263 | SP140 nuclear body protein [Source:HGNC Symbol;Acc:17133] |
| MKNK1 | ENSG00000079277 | MAP kinase interacting serine/threonine kinase 1 [Source:HGNC Symbol;Acc:7110] |
| TNS1 | ENSG00000079308 | tensin 1 [Source:HGNC Symbol;Acc:11973] |
| RAPGEF3 | ENSG00000079337 | Rap guanine nucleotide exchange factor (GEF) 3 [Source:HGNC Symbol;Acc:16629] |
| CEACAM1 | ENSG00000079385 | carcinoembryonic antigen-related cell adhesion molecule 1 (biliary glycoprotein) [Source:HGNC Symbol;Acc:1814] |
| RIMS1 | ENSG00000079841 | regulating synaptic membrane exocytosis 1 [Source:HGNC Symbol;Acc:17282] |
| PTPRH | ENSG00000080031 | protein tyrosine phosphatase, receptor type, H [Source:HGNC Symbol;Acc:9672] |
| CXCL2 | ENSG00000081041 | chemokine (C-X-C motif) ligand 2 [Source:HGNC Symbol;Acc:4603] |
| MEF2C | ENSG00000081189 | myocyte enhancer factor 2C [Source:HGNC Symbol;Acc:6996] |
| LRP2 | ENSG00000081479 | low density lipoprotein receptor-related protein 2 [Source:HGNC Symbol;Acc:6694] |
| PCDHB4 | ENSG00000081818 | protocadherin beta 4 [Source:HGNC Symbol;Acc:8689] |
| PCDHGA2 | ENSG00000081853 | protocadherin gamma subfamily A, 2 [Source:HGNC Symbol;Acc:8700] |
| FYB | ENSG00000082074 | FYN binding protein [Source:HGNC Symbol;Acc:4036] |
| FAM135A | ENSG00000082269 | family with sequence similarity 135, member A [Source:HGNC Symbol;Acc:21084] |
| SERTAD4 | ENSG00000082497 | SERTA domain containing 4 [Source:HGNC Symbol;Acc:25236] |
| CYLD | ENSG00000083799 | cylindromatosis (turban tumor syndrome) [Source:HGNC Symbol;Acc:2584] |
| COL16A1 | ENSG00000084636 | collagen, type XVI, alpha 1 [Source:HGNC Symbol;Acc:2193] |
| WDFY1 | ENSG00000085449 | WD repeat and FYVE domain containing 1 [Source:HGNC Symbol;Acc:20451] |
| DNAJA1 | ENSG00000086061 | DnaJ (Hsp40) homolog, subfamily A, member 1 [Source:HGNC Symbol;Acc:5229] |
| FOLH1 | ENSG00000086205 | folate hydrolase (prostate-specific membrane antigen) 1 [Source:HGNC Symbol;Acc:3788] |
| CEACAM6 | ENSG00000086548 | carcinoembryonic antigen-related cell adhesion molecule 6 (non-specific cross reacting antigen) [Source:HGNC Symbol;Acc:1818] |
| ACHE | ENSG00000087085 | acetylcholinesterase (Yt blood group) [Source:HGNC Symbol;Acc:108] |
| SLC6A14 | ENSG00000087916 | solute carrier family 6 (amino acid transporter), member 14 [Source:HGNC Symbol;Acc:11047] |
| EBF4 | ENSG00000088881 | early B-cell factor 4 [Source:HGNC Symbol;Acc:29278] |
| OAS1 | ENSG00000089127 | 2'-5'-oligoadenylate synthetase 1, 40/46kDa [Source:HGNC Symbol;Acc:8086] |
| FXYD3 | ENSG00000089356 | FXYD domain containing ion transport regulator 3 [Source:HGNC Symbol;Acc:4027] |
| LAG3 | ENSG00000089692 | lymphocyte-activation gene 3 [Source:HGNC Symbol;Acc:6476] |
| ICAM1 | ENSG00000090339 | intercellular adhesion molecule 1 [Source:HGNC Symbol;Acc:5344] |
| IRAK3 | ENSG00000090376 | interleukin-1 receptor-associated kinase 3 [Source:HGNC Symbol;Acc:17020] |
| FCGBP | ENSG00000090920 | Fc fragment of IgG binding protein [Source:HGNC Symbol;Acc:13572] |
| NRCAM | ENSG00000091129 | neuronal cell adhesion molecule [Source:HGNC Symbol;Acc:7994] |
| SLC26A4 | ENSG00000091137 | solute carrier family 26 (anion exchanger), member 4 [Source:HGNC Symbol;Acc:8818] |
| ZFHX4 | ENSG00000091656 | zinc finger homeobox 4 [Source:HGNC Symbol;Acc:30939] |
| CD200 | ENSG00000091972 | CD200 molecule [Source:HGNC Symbol;Acc:7203] |
| RNF31 | ENSG00000092098 | ring finger protein 31 [Source:HGNC Symbol;Acc:16031] |
| TGM1 | ENSG00000092295 | transglutaminase 1 [Source:HGNC Symbol;Acc:11777] |
| TINF2 | ENSG00000092330 | TERF1 (TRF1)-interacting nuclear factor 2 [Source:HGNC Symbol;Acc:11824] |
| SEMA6A | ENSG00000092421 | sema domain, transmembrane domain (TM), and cytoplasmic domain, (semaphorin) 6A [Source:HGNC Symbol;Acc:10738] |
| VNN3 | ENSG00000093134 | vanin 3 [Source:HGNC Symbol;Acc:16431] |
| TLL2 | ENSG00000095587 | tolloid-like 2 [Source:HGNC Symbol;Acc:11844] |
| FKBP5 | ENSG00000096060 | FK506 binding protein 5 [Source:HGNC Symbol;Acc:3721] |
| JAK2 | ENSG00000096968 | Janus kinase 2 [Source:HGNC Symbol;Acc:6192] |
| SCD | ENSG00000099194 | stearoyl-CoA desaturase (delta-9-desaturase) [Source:HGNC Symbol;Acc:10571] |
| NRP1 | ENSG00000099250 | neuropilin 1 [Source:HGNC Symbol;Acc:8004] |
| HSD3B7 | ENSG00000099377 | hydroxy-delta-5-steroid dehydrogenase, 3 beta- and steroid delta-isomerase 7 [Source:HGNC Symbol;Acc:18324] |
| BCL2L13 | ENSG00000099968 | BCL2-like 13 (apoptosis facilitator) [Source:HGNC Symbol;Acc:17164] |
| SUSD2 | ENSG00000099994 | sushi domain containing 2 [Source:HGNC Symbol;Acc:30667] |
| ADRBK2 | ENSG00000100077 | adrenergic, beta, receptor kinase 2 [Source:HGNC Symbol;Acc:290] |
| PLA2G3 | ENSG00000100078 | phospholipase A2, group III [Source:HGNC Symbol;Acc:17934] |
| HIRA | ENSG00000100084 | histone cell cycle regulator [Source:HGNC Symbol;Acc:4916] |
| TPTEP1 | ENSG00000100181 | transmembrane phosphatase with tensin homology pseudogene 1 [Source:HGNC Symbol;Acc:43648] |
| GTPBP1 | ENSG00000100226 | GTP binding protein 1 [Source:HGNC Symbol;Acc:4669] |
| RHBDD3 | ENSG00000100263 | rhomboid domain containing 3 [Source:HGNC Symbol;Acc:1308] |
| HMOX1 | ENSG00000100292 | heme oxygenase (decycling) 1 [Source:HGNC Symbol;Acc:5013] |
| ARSA | ENSG00000100299 | arylsulfatase A [Source:HGNC Symbol;Acc:713] |
| APOL4 | ENSG00000100336 | apolipoprotein L, 4 [Source:HGNC Symbol;Acc:14867] |
| APOL1 | ENSG00000100342 | apolipoprotein L, 1 [Source:HGNC Symbol;Acc:618] |
| CACNA1I | ENSG00000100346 | calcium channel, voltage-dependent, T type, alpha 1I subunit [Source:HGNC Symbol;Acc:1396] |
| RANGAP1 | ENSG00000100401 | Ran GTPase activating protein 1 [Source:HGNC Symbol;Acc:9854] |
| ZC3H7B | ENSG00000100403 | zinc finger CCCH-type containing 7B [Source:HGNC Symbol;Acc:30869] |
| COCH | ENSG00000100473 | cochlin [Source:HGNC Symbol;Acc:2180] |
| PLEK2 | ENSG00000100558 | pleckstrin 2 [Source:HGNC Symbol;Acc:19238] |
| LGMN | ENSG00000100600 | legumain [Source:HGNC Symbol;Acc:9472] |
| EFS | ENSG00000100842 | embryonal Fyn-associated substrate [Source:HGNC Symbol;Acc:16898] |
| PSMA6 | ENSG00000100902 | proteasome (prosome, macropain) subunit, alpha type, 6 [Source:HGNC Symbol;Acc:9535] |
| NFKBIA | ENSG00000100906 | nuclear factor of kappa light polypeptide gene enhancer in B-cells inhibitor, alpha [Source:HGNC Symbol;Acc:7797] |
| PSME2 | ENSG00000100911 | proteasome (prosome, macropain) activator subunit 2 (PA28 beta) [Source:HGNC Symbol;Acc:9569] |
| REC8 | ENSG00000100918 | REC8 meiotic recombination protein [Source:HGNC Symbol;Acc:16879] |
| MMP9 | ENSG00000100985 | matrix metallopeptidase 9 (gelatinase B, 92kDa gelatinase, 92kDa type IV collagenase) [Source:HGNC Symbol;Acc:7176] |
| PROCR | ENSG00000101000 | protein C receptor, endothelial [Source:HGNC Symbol;Acc:9452] |
| CD40 | ENSG00000101017 | CD40 molecule, TNF receptor superfamily member 5 [Source:HGNC Symbol;Acc:11919] |
| ZMYND8 | ENSG00000101040 | zinc finger, MYND-type containing 8 [Source:HGNC Symbol;Acc:9397] |
| SALL4 | ENSG00000101115 | spalt-like transcription factor 4 [Source:HGNC Symbol;Acc:15924] |
| SLCO4A1 | ENSG00000101187 | solute carrier organic anion transporter family, member 4A1 [Source:HGNC Symbol;Acc:10953] |
| CDC25B | ENSG00000101224 | cell division cycle 25B [Source:HGNC Symbol;Acc:1726] |
| RASSF2 | ENSG00000101265 | Ras association (RalGDS/AF-6) domain family member 2 [Source:HGNC Symbol;Acc:9883] |
| SNPH | ENSG00000101298 | syntaphilin [Source:HGNC Symbol;Acc:15931] |
| FERMT1 | ENSG00000101311 | fermitin family member 1 [Source:HGNC Symbol;Acc:15889] |
| TLDC2 | ENSG00000101342 | TBC/LysM-associated domain containing 2 [Source:HGNC Symbol;Acc:16112] |
| SAMHD1 | ENSG00000101347 | SAM domain and HD domain 1 [Source:HGNC Symbol;Acc:15925] |
| SNTA1 | ENSG00000101400 | syntrophin, alpha 1 [Source:HGNC Symbol;Acc:11167] |
| WFDC2 | ENSG00000101443 | WAP four-disulfide core domain 2 [Source:HGNC Symbol;Acc:15939] |
| PPP1R16B | ENSG00000101445 | protein phosphatase 1, regulatory subunit 16B [Source:HGNC Symbol;Acc:15850] |
| SMCHD1 | ENSG00000101596 | structural maintenance of chromosomes flexible hinge domain containing 1 [Source:HGNC Symbol;Acc:29090] |
| LIPG | ENSG00000101670 | lipase, endothelial [Source:HGNC Symbol;Acc:6623] |
| LAMA1 | ENSG00000101680 | laminin, alpha 1 [Source:HGNC Symbol;Acc:6481] |
| MXRA5 | ENSG00000101825 | matrix-remodelling associated 5 [Source:HGNC Symbol;Acc:7539] |
| STS | ENSG00000101846 | steroid sulfatase (microsomal), isozyme S [Source:HGNC Symbol;Acc:11425] |
| ABCD1 | ENSG00000101986 | ATP-binding cassette, sub-family D (ALD), member 1 [Source:HGNC Symbol;Acc:61] |
| BMX | ENSG00000102010 | BMX non-receptor tyrosine kinase [Source:HGNC Symbol;Acc:1079] |
| FMR1 | ENSG00000102081 | fragile X mental retardation 1 [Source:HGNC Symbol;Acc:3775] |
| RP2 | ENSG00000102218 | retinitis pigmentosa 2 (X-linked recessive) [Source:HGNC Symbol;Acc:10274] |
| GABRE | ENSG00000102287 | gamma-aminobutyric acid (GABA) A receptor, epsilon [Source:HGNC Symbol;Acc:4085] |
| SRPX2 | ENSG00000102359 | sushi-repeat containing protein, X-linked 2 [Source:HGNC Symbol;Acc:30668] |
| TNFSF13B | ENSG00000102524 | tumor necrosis factor (ligand) superfamily, member 13b [Source:HGNC Symbol;Acc:11929] |
| FNDC3A | ENSG00000102531 | fibronectin type III domain containing 3A [Source:HGNC Symbol;Acc:20296] |
| ACP5 | ENSG00000102575 | acid phosphatase 5, tartrate resistant [Source:HGNC Symbol;Acc:124] |
| PARP4 | ENSG00000102699 | poly (ADP-ribose) polymerase family, member 4 [Source:HGNC Symbol;Acc:271] |
| FLT1 | ENSG00000102755 | fms-related tyrosine kinase 1 [Source:HGNC Symbol;Acc:3763] |
| MSLN | ENSG00000102854 | mesothelin [Source:HGNC Symbol;Acc:7371] |
| N4BP1 | ENSG00000102921 | NEDD4 binding protein 1 [Source:HGNC Symbol;Acc:29850] |
| CCL22 | ENSG00000102962 | chemokine (C-C motif) ligand 22 [Source:HGNC Symbol;Acc:10621] |
| CCL17 | ENSG00000102970 | chemokine (C-C motif) ligand 17 [Source:HGNC Symbol;Acc:10615] |
| HAS3 | ENSG00000103044 | hyaluronan synthase 3 [Source:HGNC Symbol;Acc:4820] |
| TANGO6 | ENSG00000103047 | transport and golgi organization 6 homolog (Drosophila) [Source:HGNC Symbol;Acc:25749] |
| FA2H | ENSG00000103089 | fatty acid 2-hydroxylase [Source:HGNC Symbol;Acc:21197] |
| CLCN7 | ENSG00000103249 | chloride channel, voltage-sensitive 7 [Source:HGNC Symbol;Acc:2025] |
| CRYM | ENSG00000103316 | crystallin, mu [Source:HGNC Symbol;Acc:2418] |
| TOX3 | ENSG00000103460 | TOX high mobility group box family member 3 [Source:HGNC Symbol;Acc:11972] |
| EHD4 | ENSG00000103966 | EH-domain containing 4 [Source:HGNC Symbol;Acc:3245] |
| RHOV | ENSG00000104140 | ras homolog family member V [Source:HGNC Symbol;Acc:18313] |
| PDGFRL | ENSG00000104213 | platelet-derived growth factor receptor-like [Source:HGNC Symbol;Acc:8805] |
| RIPK2 | ENSG00000104312 | receptor-interacting serine-threonine kinase 2 [Source:HGNC Symbol;Acc:10020] |
| NBN | ENSG00000104320 | nibrin [Source:HGNC Symbol;Acc:7652] |
| CALB1 | ENSG00000104327 | calbindin 1, 28kDa [Source:HGNC Symbol;Acc:1434] |
| SFRP1 | ENSG00000104332 | secreted frizzled-related protein 1 [Source:HGNC Symbol;Acc:10776] |
| PLAT | ENSG00000104368 | plasminogen activator, tissue [Source:HGNC Symbol;Acc:9051] |
| WISP1 | ENSG00000104415 | WNT1 inducible signaling pathway protein 1 [Source:HGNC Symbol;Acc:12769] |
| IL7 | ENSG00000104432 | interleukin 7 [Source:HGNC Symbol;Acc:6023] |
| NCALD | ENSG00000104490 | neurocalcin delta [Source:HGNC Symbol;Acc:7655] |
| GSDMD | ENSG00000104518 | gasdermin D [Source:HGNC Symbol;Acc:25697] |
| RELB | ENSG00000104856 | v-rel avian reticuloendotheliosis viral oncogene homolog B [Source:HGNC Symbol;Acc:9956] |
| IL4I1 | ENSG00000104951 | interleukin 4 induced 1 [Source:HGNC Symbol;Acc:19094] |
| OLFM2 | ENSG00000105088 | olfactomedin 2 [Source:HGNC Symbol;Acc:17189] |
| EBI3 | ENSG00000105246 | Epstein-Barr virus induced 3 [Source:HGNC Symbol;Acc:3129] |
| PRKD2 | ENSG00000105287 | protein kinase D2 [Source:HGNC Symbol;Acc:17293] |
| BBC3 | ENSG00000105327 | BCL2 binding component 3 [Source:HGNC Symbol;Acc:17868] |
| DENND3 | ENSG00000105339 | DENN/MADD domain containing 3 [Source:HGNC Symbol;Acc:29134] |
| MYH14 | ENSG00000105357 | myosin, heavy chain 14, non-muscle [Source:HGNC Symbol;Acc:23212] |
| CEACAM5 | ENSG00000105388 | carcinoembryonic antigen-related cell adhesion molecule 5 [Source:HGNC Symbol;Acc:1817] |
| NAPA | ENSG00000105402 | N-ethylmaleimide-sensitive factor attachment protein, alpha [Source:HGNC Symbol;Acc:7641] |
| RABAC1 | ENSG00000105404 | Rab acceptor 1 (prenylated) [Source:HGNC Symbol;Acc:9794] |
| FAM83E | ENSG00000105523 | family with sequence similarity 83, member E [Source:HGNC Symbol;Acc:25972] |
| PLEKHA4 | ENSG00000105559 | pleckstrin homology domain containing, family A (phosphoinositide binding specific) member 4 [Source:HGNC Symbol;Acc:14339] |
| LSR | ENSG00000105699 | lipolysis stimulated lipoprotein receptor [Source:HGNC Symbol;Acc:29572] |
| TFPI2 | ENSG00000105825 | tissue factor pathway inhibitor 2 [Source:HGNC Symbol;Acc:11761] |
| PTN | ENSG00000105894 | pleiotrophin [Source:HGNC Symbol;Acc:9630] |
| ZC3HAV1 | ENSG00000105939 | zinc finger CCCH-type, antiviral 1 [Source:HGNC Symbol;Acc:23721] |
| ADAP1 | ENSG00000105963 | ArfGAP with dual PH domains 1 [Source:HGNC Symbol;Acc:16486] |
| NOD1 | ENSG00000106100 | nucleotide-binding oligomerization domain containing 1 [Source:HGNC Symbol;Acc:16390] |
| PTPRZ1 | ENSG00000106278 | protein tyrosine phosphatase, receptor-type, Z polypeptide 1 [Source:HGNC Symbol;Acc:9685] |
| C1GALT1 | ENSG00000106392 | core 1 synthase, glycoprotein-N-acetylgalactosamine 3-beta-galactosyltransferase, 1 [Source:HGNC Symbol;Acc:24337] |
| AGR2 | ENSG00000106541 | anterior gradient 2 [Source:HGNC Symbol;Acc:328] |
| GIMAP2 | ENSG00000106560 | GTPase, IMAP family member 2 [Source:HGNC Symbol;Acc:21789] |
| PSMA2 | ENSG00000106588 | proteasome (prosome, macropain) subunit, alpha type, 2 [Source:HGNC Symbol;Acc:9531] |
| TRIM14 | ENSG00000106785 | tripartite motif containing 14 [Source:HGNC Symbol;Acc:16283] |
| TLE4 | ENSG00000106829 | transducin-like enhancer of split 4 (E(sp1) homolog, Drosophila) [Source:HGNC Symbol;Acc:11840] |
| DDX58 | ENSG00000107201 | DEAD (Asp-Glu-Ala-Asp) box polypeptide 58 [Source:HGNC Symbol;Acc:19102] |
| GATA3 | ENSG00000107485 | GATA binding protein 3 [Source:HGNC Symbol;Acc:4172] |
| ATRNL1 | ENSG00000107518 | attractin-like 1 [Source:HGNC Symbol;Acc:29063] |
| CDH23 | ENSG00000107736 | cadherin-related 23 [Source:HGNC Symbol;Acc:13733] |
| C10orf54 | ENSG00000107738 | chromosome 10 open reading frame 54 [Source:HGNC Symbol;Acc:30085] |
| LIPA | ENSG00000107798 | lipase A, lysosomal acid, cholesterol esterase [Source:HGNC Symbol;Acc:6617] |
| MAP3K8 | ENSG00000107968 | mitogen-activated protein kinase kinase kinase 8 [Source:HGNC Symbol;Acc:6860] |
| CYP2C18 | ENSG00000108242 | cytochrome P450, family 2, subfamily C, polypeptide 18 [Source:HGNC Symbol;Acc:2620] |
| CSF3 | ENSG00000108342 | colony stimulating factor 3 (granulocyte) [Source:HGNC Symbol;Acc:2438] |
| RNF43 | ENSG00000108375 | ring finger protein 43 [Source:HGNC Symbol;Acc:18505] |
| 4-Sep | ENSG00000108387 | septin 4 [Source:HGNC Symbol;Acc:9165] |
| LGALS3BP | ENSG00000108679 | lectin, galactoside-binding, soluble, 3 binding protein [Source:HGNC Symbol;Acc:6564] |
| DHX58 | ENSG00000108771 | DEXH (Asp-Glu-X-His) box polypeptide 58 [Source:HGNC Symbol;Acc:29517] |
| COL1A1 | ENSG00000108821 | collagen, type I, alpha 1 [Source:HGNC Symbol;Acc:2197] |
| HLF | ENSG00000108924 | hepatic leukemia factor [Source:HGNC Symbol;Acc:4977] |
| SLC16A6 | ENSG00000108932 | solute carrier family 16, member 6 [Source:HGNC Symbol;Acc:10927] |
| MAP2K6 | ENSG00000108984 | mitogen-activated protein kinase kinase 6 [Source:HGNC Symbol;Acc:6846] |
| FOXN1 | ENSG00000109101 | forkhead box N1 [Source:HGNC Symbol;Acc:12765] |
| ANXA10 | ENSG00000109511 | annexin A10 [Source:HGNC Symbol;Acc:534] |
| SOD3 | ENSG00000109610 | superoxide dismutase 3, extracellular [Source:HGNC Symbol;Acc:11181] |
| CTSC | ENSG00000109861 | cathepsin C [Source:HGNC Symbol;Acc:2528] |
| EHD1 | ENSG00000110047 | EH-domain containing 1 [Source:HGNC Symbol;Acc:3242] |
| UNC93B1 | ENSG00000110057 | unc-93 homolog B1 (C. elegans) [Source:HGNC Symbol;Acc:13481] |
| CCND1 | ENSG00000110092 | cyclin D1 [Source:HGNC Symbol;Acc:1582] |
| FOLR1 | ENSG00000110195 | folate receptor 1 (adult) [Source:HGNC Symbol;Acc:3791] |
| PANX1 | ENSG00000110218 | pannexin 1 [Source:HGNC Symbol;Acc:8599] |
| SLC15A3 | ENSG00000110446 | solute carrier family 15 (oligopeptide transporter), member 3 [Source:HGNC Symbol;Acc:18068] |
| ACCS | ENSG00000110455 | 1-aminocyclopropane-1-carboxylate synthase homolog (Arabidopsis)(non-functional) [Source:HGNC Symbol;Acc:23989] |
| MDK | ENSG00000110492 | midkine (neurite growth-promoting factor 2) [Source:HGNC Symbol;Acc:6972] |
| CLEC2B | ENSG00000110852 | C-type lectin domain family 2, member B [Source:HGNC Symbol;Acc:2053] |
| SLC11A2 | ENSG00000110911 | solute carrier family 11 (proton-coupled divalent metal ion transporter), member 2 [Source:HGNC Symbol;Acc:10908] |
| SLC6A12 | ENSG00000111181 | solute carrier family 6 (neurotransmitter transporter), member 12 [Source:HGNC Symbol;Acc:11045] |
| AKAP3 | ENSG00000111254 | A kinase (PRKA) anchor protein 3 [Source:HGNC Symbol;Acc:373] |
| OAS3 | ENSG00000111331 | 2'-5'-oligoadenylate synthetase 3, 100kDa [Source:HGNC Symbol;Acc:8088] |
| OAS2 | ENSG00000111335 | 2'-5'-oligoadenylate synthetase 2, 69/71kDa [Source:HGNC Symbol;Acc:8087] |
| TIMELESS | ENSG00000111602 | timeless circadian clock [Source:HGNC Symbol;Acc:11813] |
| SLCO1B3 | ENSG00000111700 | solute carrier organic anion transporter family, member 1B3 [Source:HGNC Symbol;Acc:10961] |
| BTN3A3 | ENSG00000111801 | butyrophilin, subfamily 3, member A3 [Source:HGNC Symbol;Acc:1140] |
| MAN1A1 | ENSG00000111885 | mannosidase, alpha, class 1A, member 1 [Source:HGNC Symbol;Acc:6821] |
| NCOA7 | ENSG00000111912 | nuclear receptor coactivator 7 [Source:HGNC Symbol;Acc:21081] |
| FBXO5 | ENSG00000112029 | F-box protein 5 [Source:HGNC Symbol;Acc:13584] |
| SOD2 | ENSG00000112096 | superoxide dismutase 2, mitochondrial [Source:HGNC Symbol;Acc:11180] |
| MDGA1 | ENSG00000112139 | MAM domain containing glycosylphosphatidylinositol anchor 1 [Source:HGNC Symbol;Acc:19267] |
| CD83 | ENSG00000112149 | CD83 molecule [Source:HGNC Symbol;Acc:1703] |
| BVES | ENSG00000112276 | blood vessel epicardial substance [Source:HGNC Symbol;Acc:1152] |
| VNN1 | ENSG00000112299 | vanin 1 [Source:HGNC Symbol;Acc:12705] |
| VNN2 | ENSG00000112303 | vanin 2 [Source:HGNC Symbol;Acc:12706] |
| TRIM38 | ENSG00000112343 | tripartite motif containing 38 [Source:HGNC Symbol;Acc:10059] |
| PDE10A | ENSG00000112541 | phosphodiesterase 10A [Source:HGNC Symbol;Acc:8772] |
| CCND3 | ENSG00000112576 | cyclin D3 [Source:HGNC Symbol;Acc:1585] |
| DUSP22 | ENSG00000112679 | dual specificity phosphatase 22 [Source:HGNC Symbol;Acc:16077] |
| FAM46A | ENSG00000112773 | family with sequence similarity 46, member A [Source:HGNC Symbol;Acc:18345] |
| PCDHB2 | ENSG00000112852 | protocadherin beta 2 [Source:HGNC Symbol;Acc:8687] |
| HMGCS1 | ENSG00000112972 | 3-hydroxy-3-methylglutaryl-CoA synthase 1 (soluble) [Source:HGNC Symbol;Acc:5007] |
| LOX | ENSG00000113083 | lysyl oxidase [Source:HGNC Symbol;Acc:6664] |
| PCDHB6 | ENSG00000113211 | protocadherin beta 6 [Source:HGNC Symbol;Acc:8691] |
| PCDHB15 | ENSG00000113248 | protocadherin beta 15 [Source:HGNC Symbol;Acc:8686] |
| ARSB | ENSG00000113273 | arylsulfatase B [Source:HGNC Symbol;Acc:714] |
| IL12B | ENSG00000113302 | interleukin 12B (natural killer cell stimulatory factor 2, cytotoxic lymphocyte maturation factor 2, p40) [Source:HGNC Symbol;Acc:5970] |
| LMNB1 | ENSG00000113368 | lamin B1 [Source:HGNC Symbol;Acc:6637] |
| ARRDC3 | ENSG00000113369 | arrestin domain containing 3 [Source:HGNC Symbol;Acc:29263] |
| LNPEP | ENSG00000113441 | leucyl/cystinyl aminopeptidase [Source:HGNC Symbol;Acc:6656] |
| PDE4D | ENSG00000113448 | phosphodiesterase 4D, cAMP-specific [Source:HGNC Symbol;Acc:8783] |
| ST8SIA4 | ENSG00000113532 | ST8 alpha-N-acetyl-neuraminide alpha-2,8-sialyltransferase 4 [Source:HGNC Symbol;Acc:10871] |
| LIFR | ENSG00000113594 | leukemia inhibitory factor receptor alpha [Source:HGNC Symbol;Acc:6597] |
| HGD | ENSG00000113924 | homogentisate 1,2-dioxygenase [Source:HGNC Symbol;Acc:4892] |
| XRN1 | ENSG00000114127 | 5'-3' exoribonuclease 1 [Source:HGNC Symbol;Acc:30654] |
| KAT2B | ENSG00000114166 | K(lysine) acetyltransferase 2B [Source:HGNC Symbol;Acc:8638] |
| LRRC31 | ENSG00000114248 | leucine rich repeat containing 31 [Source:HGNC Symbol;Acc:26261] |
| HYAL1 | ENSG00000114378 | hyaluronoglucosaminidase 1 [Source:HGNC Symbol;Acc:5320] |
| GNB4 | ENSG00000114450 | guanine nucleotide binding protein (G protein), beta polypeptide 4 [Source:HGNC Symbol;Acc:20731] |
| UPK1B | ENSG00000114638 | uroplakin 1B [Source:HGNC Symbol;Acc:12578] |
| PLSCR4 | ENSG00000114698 | phospholipid scramblase 4 [Source:HGNC Symbol;Acc:16497] |
| PEX5L | ENSG00000114757 | peroxisomal biogenesis factor 5-like [Source:HGNC Symbol;Acc:30024] |
| PLCH1 | ENSG00000114805 | phospholipase C, eta 1 [Source:HGNC Symbol;Acc:29185] |
| CLCN2 | ENSG00000114859 | chloride channel, voltage-sensitive 2 [Source:HGNC Symbol;Acc:2020] |
| IL1A | ENSG00000115008 | interleukin 1, alpha [Source:HGNC Symbol;Acc:5991] |
| CCL20 | ENSG00000115009 | chemokine (C-C motif) ligand 20 [Source:HGNC Symbol;Acc:10619] |
| GPD2 | ENSG00000115159 | glycerol-3-phosphate dehydrogenase 2 (mitochondrial) [Source:HGNC Symbol;Acc:4456] |
| IFIH1 | ENSG00000115267 | interferon induced with helicase C domain 1 [Source:HGNC Symbol;Acc:18873] |
| GCA | ENSG00000115271 | grancalcin, EF-hand calcium binding protein [Source:HGNC Symbol;Acc:15990] |
| STAT1 | ENSG00000115415 | signal transducer and activator of transcription 1, 91kDa [Source:HGNC Symbol;Acc:11362] |
| IGFBP2 | ENSG00000115457 | insulin-like growth factor binding protein 2, 36kDa [Source:HGNC Symbol;Acc:5471] |
| IGFBP5 | ENSG00000115461 | insulin-like growth factor binding protein 5 [Source:HGNC Symbol;Acc:5474] |
| IL1R2 | ENSG00000115590 | interleukin 1 receptor, type II [Source:HGNC Symbol;Acc:5994] |
| QPCT | ENSG00000115828 | glutaminyl-peptide cyclotransferase [Source:HGNC Symbol;Acc:9753] |
| PLCL1 | ENSG00000115896 | phospholipase C-like 1 [Source:HGNC Symbol;Acc:9063] |
| KYNU | ENSG00000115919 | kynureninase [Source:HGNC Symbol;Acc:6469] |
| WIPF1 | ENSG00000115935 | WAS/WASL interacting protein family, member 1 [Source:HGNC Symbol;Acc:12736] |
| EPAS1 | ENSG00000116016 | endothelial PAS domain protein 1 [Source:HGNC Symbol;Acc:3374] |
| EPHA4 | ENSG00000116106 | EPH receptor A4 [Source:HGNC Symbol;Acc:3388] |
| ANGPTL1 | ENSG00000116194 | angiopoietin-like 1 [Source:HGNC Symbol;Acc:489] |
| KIAA1324 | ENSG00000116299 | KIAA1324 [Source:HGNC Symbol;Acc:29618] |
| RNF19B | ENSG00000116514 | ring finger protein 19B [Source:HGNC Symbol;Acc:26886] |
| FBXO6 | ENSG00000116663 | F-box protein 6 [Source:HGNC Symbol;Acc:13585] |
| CFHR3 | ENSG00000116785 | complement factor H-related 3 [Source:HGNC Symbol;Acc:16980] |
| TTF2 | ENSG00000116830 | transcription termination factor, RNA polymerase II [Source:HGNC Symbol;Acc:12398] |
| KCNQ4 | ENSG00000117013 | potassium voltage-gated channel, KQT-like subfamily, member 4 [Source:HGNC Symbol;Acc:6298] |
| AKT3 | ENSG00000117020 | v-akt murine thymoma viral oncogene homolog 3 [Source:HGNC Symbol;Acc:393] |
| LPHN2 | ENSG00000117114 | latrophilin 2 [Source:HGNC Symbol;Acc:18582] |
| GBP3 | ENSG00000117226 | guanylate binding protein 3 [Source:HGNC Symbol;Acc:4184] |
| GBP1 | ENSG00000117228 | guanylate binding protein 1, interferon-inducible [Source:HGNC Symbol;Acc:4182] |
| CDK18 | ENSG00000117266 | cyclin-dependent kinase 18 [Source:HGNC Symbol;Acc:8751] |
| TXNIP | ENSG00000117289 | thioredoxin interacting protein [Source:HGNC Symbol;Acc:16952] |
| ECE1 | ENSG00000117298 | endothelin converting enzyme 1 [Source:HGNC Symbol;Acc:3146] |
| BLZF1 | ENSG00000117475 | basic leucine zipper nuclear factor 1 [Source:HGNC Symbol;Acc:1065] |
| HSD11B1 | ENSG00000117594 | hydroxysteroid (11-beta) dehydrogenase 1 [Source:HGNC Symbol;Acc:5208] |
| IRF6 | ENSG00000117595 | interferon regulatory factor 6 [Source:HGNC Symbol;Acc:6121] |
| PROX1 | ENSG00000117707 | prospero homeobox 1 [Source:HGNC Symbol;Acc:9459] |
| MUC5B | ENSG00000117983 | mucin 5B, oligomeric mucus/gel-forming [Source:HGNC Symbol;Acc:7516] |
| NRP2 | ENSG00000118257 | neuropilin 2 [Source:HGNC Symbol;Acc:8005] |
| ATP10B | ENSG00000118322 | ATPase, class V, type 10B [Source:HGNC Symbol;Acc:13543] |
| PLAGL1 | ENSG00000118495 | pleiomorphic adenoma gene-like 1 [Source:HGNC Symbol;Acc:9046] |
| TNFAIP3 | ENSG00000118503 | tumor necrosis factor, alpha-induced protein 3 [Source:HGNC Symbol;Acc:11896] |
| AKAP7 | ENSG00000118507 | A kinase (PRKA) anchor protein 7 [Source:HGNC Symbol;Acc:377] |
| SGK1 | ENSG00000118515 | serum/glucocorticoid regulated kinase 1 [Source:HGNC Symbol;Acc:10810] |
| RARRES1 | ENSG00000118849 | retinoic acid receptor responder (tazarotene induced) 1 [Source:HGNC Symbol;Acc:9867] |
| PCDH17 | ENSG00000118946 | protocadherin 17 [Source:HGNC Symbol;Acc:14267] |
| GLRXP3 | ENSG00000118990 | glutaredoxin (thioltransferase) pseudogene 3 [Source:HGNC Symbol;Acc:34049] |
| GDA | ENSG00000119125 | guanine deaminase [Source:HGNC Symbol;Acc:4212] |
| GALNT12 | ENSG00000119514 | UDP-N-acetyl-alpha-D-galactosamine:polypeptide N-acetylgalactosaminyltransferase 12 (GalNAc-T12) [Source:HGNC Symbol;Acc:19877] |
| GPR68 | ENSG00000119714 | G protein-coupled receptor 68 [Source:HGNC Symbol;Acc:4519] |
| FKBP1B | ENSG00000119782 | FK506 binding protein 1B, 12.6 kDa [Source:HGNC Symbol;Acc:3712] |
| IFIT3 | ENSG00000119917 | interferon-induced protein with tetratricopeptide repeats 3 [Source:HGNC Symbol;Acc:5411] |
| IFIT2 | ENSG00000119922 | interferon-induced protein with tetratricopeptide repeats 2 [Source:HGNC Symbol;Acc:5409] |
| CD274 | ENSG00000120217 | CD274 molecule [Source:HGNC Symbol;Acc:17635] |
| NUDCD1 | ENSG00000120526 | NudC domain containing 1 [Source:HGNC Symbol;Acc:24306] |
| MASTL | ENSG00000120539 | microtubule associated serine/threonine kinase-like [Source:HGNC Symbol;Acc:19042] |
| KIAA1217 | ENSG00000120549 | KIAA1217 [Source:HGNC Symbol;Acc:25428] |
| DNAJC15 | ENSG00000120675 | DnaJ (Hsp40) homolog, subfamily C, member 15 [Source:HGNC Symbol;Acc:20325] |
| EGR1 | ENSG00000120738 | early growth response 1 [Source:HGNC Symbol;Acc:3238] |
| SOCS2 | ENSG00000120833 | suppressor of cytokine signaling 2 [Source:HGNC Symbol;Acc:19382] |
| PTK2B | ENSG00000120899 | protein tyrosine kinase 2 beta [Source:HGNC Symbol;Acc:9612] |
| TRIM25 | ENSG00000121060 | tripartite motif containing 25 [Source:HGNC Symbol;Acc:12932] |
| PLBD1 | ENSG00000121316 | phospholipase B domain containing 1 [Source:HGNC Symbol;Acc:26215] |
| BCL2L14 | ENSG00000121380 | BCL2-like 14 (apoptosis facilitator) [Source:HGNC Symbol;Acc:16657] |
| CSTA | ENSG00000121552 | cystatin A (stefin A) [Source:HGNC Symbol;Acc:2481] |
| PILRB | ENSG00000121716 | paired immunoglobin-like type 2 receptor beta [Source:HGNC Symbol;Acc:18297] |
| CCRL2 | ENSG00000121797 | chemokine (C-C motif) receptor-like 2 [Source:HGNC Symbol;Acc:1612] |
| TNFSF10 | ENSG00000121858 | tumor necrosis factor (ligand) superfamily, member 10 [Source:HGNC Symbol;Acc:11925] |
| TMEM54 | ENSG00000121900 | transmembrane protein 54 [Source:HGNC Symbol;Acc:24143] |
| FYTTD1 | ENSG00000122068 | forty-two-three domain containing 1 [Source:HGNC Symbol;Acc:25407] |
| NT5C3A | ENSG00000122643 | 5'-nucleotidase, cytosolic IIIA [Source:HGNC Symbol;Acc:17820] |
| GLIPR2 | ENSG00000122694 | GLI pathogenesis-related 2 [Source:HGNC Symbol;Acc:18007] |
| C7orf49 | ENSG00000122783 | chromosome 7 open reading frame 49 [Source:HGNC Symbol;Acc:22432] |
| SRGN | ENSG00000122862 | serglycin [Source:HGNC Symbol;Acc:9361] |
| EGR2 | ENSG00000122877 | early growth response 2 [Source:HGNC Symbol;Acc:3239] |
| SSPN | ENSG00000123096 | sarcospan [Source:HGNC Symbol;Acc:11322] |
| ITPR2 | ENSG00000123104 | inositol 1,4,5-trisphosphate receptor, type 2 [Source:HGNC Symbol;Acc:6181] |
| OPTN | ENSG00000123240 | optineurin [Source:HGNC Symbol;Acc:17142] |
| MMP19 | ENSG00000123342 | matrix metallopeptidase 19 [Source:HGNC Symbol;Acc:7165] |
| NFE2 | ENSG00000123405 | nuclear factor, erythroid 2 [Source:HGNC Symbol;Acc:7780] |
| NMI | ENSG00000123609 | N-myc (and STAT) interactor [Source:HGNC Symbol;Acc:7854] |
| TNFAIP6 | ENSG00000123610 | tumor necrosis factor, alpha-induced protein 6 [Source:HGNC Symbol;Acc:11898] |
| G0S2 | ENSG00000123689 | G0/G1switch 2 [Source:HGNC Symbol;Acc:30229] |
| KCNJ2 | ENSG00000123700 | potassium inwardly-rectifying channel, subfamily J, member 2 [Source:HGNC Symbol;Acc:6263] |
| PI3 | ENSG00000124102 | peptidase inhibitor 3, skin-derived [Source:HGNC Symbol;Acc:8947] |
| SLPI | ENSG00000124107 | secretory leukocyte peptidase inhibitor [Source:HGNC Symbol;Acc:11092] |
| ZNFX1 | ENSG00000124201 | zinc finger, NFX1-type containing 1 [Source:HGNC Symbol;Acc:29271] |
| CDH26 | ENSG00000124215 | cadherin 26 [Source:HGNC Symbol;Acc:15902] |
| RNF114 | ENSG00000124226 | ring finger protein 114 [Source:HGNC Symbol;Acc:13094] |
| KCNK15 | ENSG00000124249 | potassium channel, subfamily K, member 15 [Source:HGNC Symbol;Acc:13814] |
| ZBP1 | ENSG00000124256 | Z-DNA binding protein 1 [Source:HGNC Symbol;Acc:16176] |
| IL17C | ENSG00000124391 | interleukin 17C [Source:HGNC Symbol;Acc:5983] |
| POF1B | ENSG00000124429 | premature ovarian failure, 1B [Source:HGNC Symbol;Acc:13711] |
| HIF3A | ENSG00000124440 | hypoxia inducible factor 3, alpha subunit [Source:HGNC Symbol;Acc:15825] |
| LYPD3 | ENSG00000124466 | LY6/PLAUR domain containing 3 [Source:HGNC Symbol;Acc:24880] |
| BTN2A2 | ENSG00000124508 | butyrophilin, subfamily 2, member A2 [Source:HGNC Symbol;Acc:1137] |
| BTN2A3P | ENSG00000124549 | butyrophilin, subfamily 2, member A3, pseudogene [Source:HGNC Symbol;Acc:13229] |
| PRICKLE4 | ENSG00000124593 | prickle homolog 4 (Drosophila) [Source:HGNC Symbol;Acc:16805] |
| HIST1H2BJ | ENSG00000124635 | histone cluster 1, H2bj [Source:HGNC Symbol;Acc:4761] |
| SPDEF | ENSG00000124664 | SAM pointed domain containing ETS transcription factor [Source:HGNC Symbol;Acc:17257] |
| EREG | ENSG00000124882 | epiregulin [Source:HGNC Symbol;Acc:3443] |
| MYRF | ENSG00000124920 | myelin regulatory factor [Source:HGNC Symbol;Acc:1181] |
| MT2A | ENSG00000125148 | metallothionein 2A [Source:HGNC Symbol;Acc:7406] |
| IRF1 | ENSG00000125347 | interferon regulatory factor 1 [Source:HGNC Symbol;Acc:6116] |
| RBCK1 | ENSG00000125826 | RanBP-type and C3HC4-type zinc finger containing 1 [Source:HGNC Symbol;Acc:15864] |
| ID1 | ENSG00000125968 | inhibitor of DNA binding 1, dominant negative helix-loop-helix protein [Source:HGNC Symbol;Acc:5360] |
| FFAR2 | ENSG00000126262 | free fatty acid receptor 2 [Source:HGNC Symbol;Acc:4501] |
| IFI6 | ENSG00000126709 | interferon, alpha-inducible protein 6 [Source:HGNC Symbol;Acc:4054] |
| SIX1 | ENSG00000126778 | SIX homeobox 1 [Source:HGNC Symbol;Acc:10887] |
| ARMCX1 | ENSG00000126947 | armadillo repeat containing, X-linked 1 [Source:HGNC Symbol;Acc:18073] |
| FGD3 | ENSG00000127084 | FYVE, RhoGEF and PH domain containing 3 [Source:HGNC Symbol;Acc:16027] |
| ATP13A4 | ENSG00000127249 | ATPase type 13A4 [Source:HGNC Symbol;Acc:25422] |
| HELB | ENSG00000127311 | helicase (DNA) B [Source:HGNC Symbol;Acc:17196] |
| TMEM175 | ENSG00000127419 | transmembrane protein 175 [Source:HGNC Symbol;Acc:28709] |
| TICAM1 | ENSG00000127666 | toll-like receptor adaptor molecule 1 [Source:HGNC Symbol;Acc:18348] |
| ZFP36 | ENSG00000128016 | ZFP36 ring finger protein [Source:HGNC Symbol;Acc:12862] |
| KDR | ENSG00000128052 | kinase insert domain receptor (a type III receptor tyrosine kinase) [Source:HGNC Symbol;Acc:6307] |
| ADM2 | ENSG00000128165 | adrenomedullin 2 [Source:HGNC Symbol;Acc:28898] |
| MGAT3 | ENSG00000128268 | mannosyl (beta-1,4-)-glycoprotein beta-1,4-N-acetylglucosaminyltransferase [Source:HGNC Symbol;Acc:7046] |
| CDC42EP1 | ENSG00000128283 | CDC42 effector protein (Rho GTPase binding) 1 [Source:HGNC Symbol;Acc:17014] |
| APOL3 | ENSG00000128284 | apolipoprotein L, 3 [Source:HGNC Symbol;Acc:14868] |
| APOL2 | ENSG00000128335 | apolipoprotein L, 2 [Source:HGNC Symbol;Acc:619] |
| LIF | ENSG00000128342 | leukemia inhibitory factor [Source:HGNC Symbol;Acc:6596] |
| APOBEC3A | ENSG00000128383 | apolipoprotein B mRNA editing enzyme, catalytic polypeptide-like 3A [Source:HGNC Symbol;Acc:17343] |
| APOBEC3F | ENSG00000128394 | apolipoprotein B mRNA editing enzyme, catalytic polypeptide-like 3F [Source:HGNC Symbol;Acc:17356] |
| DOCK4 | ENSG00000128512 | dedicator of cytokinesis 4 [Source:HGNC Symbol;Acc:19192] |
| PODXL | ENSG00000128567 | podocalyxin-like [Source:HGNC Symbol;Acc:9171] |
| STRIP2 | ENSG00000128578 | striatin interacting protein 2 [Source:HGNC Symbol;Acc:22209] |
| LRRC4 | ENSG00000128594 | leucine rich repeat containing 4 [Source:HGNC Symbol;Acc:15586] |
| CCDC136 | ENSG00000128596 | coiled-coil domain containing 136 [Source:HGNC Symbol;Acc:22225] |
| CHAC1 | ENSG00000128965 | ChaC, cation transport regulator homolog 1 (E. coli) [Source:HGNC Symbol;Acc:28680] |
| ACKR4 | ENSG00000129048 | atypical chemokine receptor 4 [Source:HGNC Symbol;Acc:1611] |
| MMP28 | ENSG00000129270 | matrix metallopeptidase 28 [Source:HGNC Symbol;Acc:14366] |
| KLK10 | ENSG00000129451 | kallikrein-related peptidase 10 [Source:HGNC Symbol;Acc:6358] |
| RIPK3 | ENSG00000129465 | receptor-interacting serine-threonine kinase 3 [Source:HGNC Symbol;Acc:10021] |
| ADCY4 | ENSG00000129467 | adenylate cyclase 4 [Source:HGNC Symbol;Acc:235] |
| RNASE1 | ENSG00000129538 | ribonuclease, RNase A family, 1 (pancreatic) [Source:HGNC Symbol;Acc:10044] |
| TEP1 | ENSG00000129566 | telomerase-associated protein 1 [Source:HGNC Symbol;Acc:11726] |
| RHBDF2 | ENSG00000129667 | rhomboid 5 homolog 2 (Drosophila) [Source:HGNC Symbol;Acc:20788] |
| FAM155B | ENSG00000130054 | family with sequence similarity 155, member B [Source:HGNC Symbol;Acc:30701] |
| SAT1 | ENSG00000130066 | spermidine/spermine N1-acetyltransferase 1 [Source:HGNC Symbol;Acc:10540] |
| LDLR | ENSG00000130164 | low density lipoprotein receptor [Source:HGNC Symbol;Acc:6547] |
| PVRL2 | ENSG00000130202 | poliovirus receptor-related 2 (herpesvirus entry mediator B) [Source:HGNC Symbol;Acc:9707] |
| ACE2 | ENSG00000130234 | angiotensin I converting enzyme 2 [Source:HGNC Symbol;Acc:13557] |
| BST2 | ENSG00000130303 | bone marrow stromal cell antigen 2 [Source:HGNC Symbol;Acc:1119] |
| KLHDC7B | ENSG00000130487 | kelch domain containing 7B [Source:HGNC Symbol;Acc:25145] |
| SCO2 | ENSG00000130489 | SCO2 cytochrome c oxidase assembly protein [Source:HGNC Symbol;Acc:10604] |
| ZBTB46 | ENSG00000130584 | zinc finger and BTB domain containing 46 [Source:HGNC Symbol;Acc:16094] |
| HELZ2 | ENSG00000130589 | helicase with zinc finger 2, transcriptional coactivator [Source:HGNC Symbol;Acc:30021] |
| SAMD10 | ENSG00000130590 | sterile alpha motif domain containing 10 [Source:HGNC Symbol;Acc:16129] |
| COL5A1 | ENSG00000130635 | collagen, type V, alpha 1 [Source:HGNC Symbol;Acc:2209] |
| THEMIS2 | ENSG00000130775 | thymocyte selection associated family member 2 [Source:HGNC Symbol;Acc:16839] |
| C19orf66 | ENSG00000130813 | chromosome 19 open reading frame 66 [Source:HGNC Symbol;Acc:25649] |
| EPS8L1 | ENSG00000131037 | EPS8-like 1 [Source:HGNC Symbol;Acc:21295] |
| ACSS2 | ENSG00000131069 | acyl-CoA synthetase short-chain family member 2 [Source:HGNC Symbol;Acc:15814] |
| IDO1 | ENSG00000131203 | indoleamine 2,3-dioxygenase 1 [Source:HGNC Symbol;Acc:6059] |
| RFTN1 | ENSG00000131378 | raftlin, lipid raft linker 1 [Source:HGNC Symbol;Acc:30278] |
| ANO1 | ENSG00000131620 | anoctamin 1, calcium activated chloride channel [Source:HGNC Symbol;Acc:21625] |
| NINJ1 | ENSG00000131669 | ninjurin 1 [Source:HGNC Symbol;Acc:7824] |
| CLUHP3 | ENSG00000131797 | clustered mitochondria (cluA/CLU1) homolog pseudogene 3 [Source:HGNC Symbol;Acc:28447] |
| GCH1 | ENSG00000131979 | GTP cyclohydrolase 1 [Source:HGNC Symbol;Acc:4193] |
| TRIM21 | ENSG00000132109 | tripartite motif containing 21 [Source:HGNC Symbol;Acc:11312] |
| TRIM5 | ENSG00000132256 | tripartite motif containing 5 [Source:HGNC Symbol;Acc:16276] |
| TRIM22 | ENSG00000132274 | tripartite motif containing 22 [Source:HGNC Symbol;Acc:16379] |
| EFR3A | ENSG00000132294 | EFR3 homolog A (S. cerevisiae) [Source:HGNC Symbol;Acc:28970] |
| CARD6 | ENSG00000132357 | caspase recruitment domain family, member 6 [Source:HGNC Symbol;Acc:16394] |
| XAF1 | ENSG00000132530 | XIAP associated factor 1 [Source:HGNC Symbol;Acc:30932] |
| MATN2 | ENSG00000132561 | matrilin 2 [Source:HGNC Symbol;Acc:6908] |
| ALDH3B2 | ENSG00000132746 | aldehyde dehydrogenase 3 family, member B2 [Source:HGNC Symbol;Acc:411] |
| CHI3L1 | ENSG00000133048 | chitinase 3-like 1 (cartilage glycoprotein-39) [Source:HGNC Symbol;Acc:1932] |
| PIK3C2B | ENSG00000133056 | phosphatidylinositol-4-phosphate 3-kinase, catalytic subunit type 2 beta [Source:HGNC Symbol;Acc:8972] |
| LGR6 | ENSG00000133067 | leucine-rich repeat containing G protein-coupled receptor 6 [Source:HGNC Symbol;Acc:19719] |
| CCNA1 | ENSG00000133101 | cyclin A1 [Source:HGNC Symbol;Acc:1577] |
| EPSTI1 | ENSG00000133106 | epithelial stromal interaction 1 (breast) [Source:HGNC Symbol;Acc:16465] |
| POSTN | ENSG00000133110 | periostin, osteoblast specific factor [Source:HGNC Symbol;Acc:16953] |
| RARRES3 | ENSG00000133321 | retinoic acid receptor responder (tazarotene induced) 3 [Source:HGNC Symbol;Acc:9869] |
| HRASLS2 | ENSG00000133328 | HRAS-like suppressor 2 [Source:HGNC Symbol;Acc:17824] |
| PDZD2 | ENSG00000133401 | PDZ domain containing 2 [Source:HGNC Symbol;Acc:18486] |
| LARGE | ENSG00000133424 | like-glycosyltransferase [Source:HGNC Symbol;Acc:6511] |
| TMTC1 | ENSG00000133687 | transmembrane and tetratricopeptide repeat containing 1 [Source:HGNC Symbol;Acc:24099] |
| IMPA1 | ENSG00000133731 | inositol(myo)-1(or 4)-monophosphatase 1 [Source:HGNC Symbol;Acc:6050] |
| IRAK2 | ENSG00000134070 | interleukin-1 receptor-associated kinase 2 [Source:HGNC Symbol;Acc:6113] |
| CHL1 | ENSG00000134121 | cell adhesion molecule L1-like [Source:HGNC Symbol;Acc:1939] |
| TSPAN2 | ENSG00000134198 | tetraspanin 2 [Source:HGNC Symbol;Acc:20659] |
| RSAD2 | ENSG00000134321 | radical S-adenosyl methionine domain containing 2 [Source:HGNC Symbol;Acc:30908] |
| CMPK2 | ENSG00000134326 | cytidine monophosphate (UMP-CMP) kinase 2, mitochondrial [Source:HGNC Symbol;Acc:27015] |
| SAA2 | ENSG00000134339 | serum amyloid A2 [Source:HGNC Symbol;Acc:10514] |
| IL15RA | ENSG00000134470 | interleukin 15 receptor, alpha [Source:HGNC Symbol;Acc:5978] |
| SOX5 | ENSG00000134532 | SRY (sex determining region Y)-box 5 [Source:HGNC Symbol;Acc:11201] |
| ACP2 | ENSG00000134575 | acid phosphatase 2, lysosomal [Source:HGNC Symbol;Acc:123] |
| FOLH1B | ENSG00000134612 | folate hydrolase 1B [Source:HGNC Symbol;Acc:13636] |
| SPOCD1 | ENSG00000134668 | SPOC domain containing 1 [Source:HGNC Symbol;Acc:26338] |
| CYP2J2 | ENSG00000134716 | cytochrome P450, family 2, subfamily J, polypeptide 2 [Source:HGNC Symbol;Acc:2634] |
| TCN1 | ENSG00000134827 | transcobalamin I (vitamin B12 binding protein, R binder family) [Source:HGNC Symbol;Acc:11652] |
| PDGFRA | ENSG00000134853 | platelet-derived growth factor receptor, alpha polypeptide [Source:HGNC Symbol;Acc:8803] |
| TBX3 | ENSG00000135111 | T-box 3 [Source:HGNC Symbol;Acc:11602] |
| OASL | ENSG00000135114 | 2'-5'-oligoadenylate synthetase-like [Source:HGNC Symbol;Acc:8090] |
| HRK | ENSG00000135116 | harakiri, BCL2 interacting protein (contains only BH3 domain) [Source:HGNC Symbol;Acc:5185] |
| TRAFD1 | ENSG00000135148 | TRAF-type zinc finger domain containing 1 [Source:HGNC Symbol;Acc:24808] |
| NT5E | ENSG00000135318 | 5'-nucleotidase, ecto (CD73) [Source:HGNC Symbol;Acc:8021] |
| LMO2 | ENSG00000135363 | LIM domain only 2 (rhombotin-like 1) [Source:HGNC Symbol;Acc:6642] |
| ESPL1 | ENSG00000135476 | extra spindle pole bodies homolog 1 (S. cerevisiae) [Source:HGNC Symbol;Acc:16856] |
| MICAL1 | ENSG00000135596 | microtubule associated monooxygenase, calponin and LIM domain containing 1 [Source:HGNC Symbol;Acc:20619] |
| STX11 | ENSG00000135604 | syntaxin 11 [Source:HGNC Symbol;Acc:11429] |
| DYSF | ENSG00000135636 | dysferlin [Source:HGNC Symbol;Acc:3097] |
| USP15 | ENSG00000135655 | ubiquitin specific peptidase 15 [Source:HGNC Symbol;Acc:12613] |
| BCMO1 | ENSG00000135697 | beta-carotene 15,15'-monooxygenase 1 [Source:HGNC Symbol;Acc:13815] |
| FAM129A | ENSG00000135842 | family with sequence similarity 129, member A [Source:HGNC Symbol;Acc:16784] |
| GPR55 | ENSG00000135898 | G protein-coupled receptor 55 [Source:HGNC Symbol;Acc:4511] |
| SP110 | ENSG00000135899 | SP110 nuclear body protein [Source:HGNC Symbol;Acc:5401] |
| HTR2B | ENSG00000135914 | 5-hydroxytryptamine (serotonin) receptor 2B, G protein-coupled [Source:HGNC Symbol;Acc:5294] |
| ANKRD36 | ENSG00000135976 | ankyrin repeat domain 36 [Source:HGNC Symbol;Acc:24079] |
| DRAM1 | ENSG00000136048 | DNA-damage regulated autophagy modulator 1 [Source:HGNC Symbol;Acc:25645] |
| KIAA1033 | ENSG00000136051 | KIAA1033 [Source:HGNC Symbol;Acc:29174] |
| CKAP2 | ENSG00000136108 | cytoskeleton associated protein 2 [Source:HGNC Symbol;Acc:1990] |
| THSD1 | ENSG00000136114 | thrombospondin, type I, domain containing 1 [Source:HGNC Symbol;Acc:17754] |
| PHF11 | ENSG00000136147 | PHD finger protein 11 [Source:HGNC Symbol;Acc:17024] |
| ITM2B | ENSG00000136156 | integral membrane protein 2B [Source:HGNC Symbol;Acc:6174] |
| GPNMB | ENSG00000136235 | glycoprotein (transmembrane) nmb [Source:HGNC Symbol;Acc:4462] |
| RAPGEF5 | ENSG00000136237 | Rap guanine nucleotide exchange factor (GEF) 5 [Source:HGNC Symbol;Acc:16862] |
| IL6 | ENSG00000136244 | interleukin 6 (interferon, beta 2) [Source:HGNC Symbol;Acc:6018] |
| CIB2 | ENSG00000136425 | calcium and integrin binding family member 2 [Source:HGNC Symbol;Acc:24579] |
| BRIP1 | ENSG00000136492 | BRCA1 interacting protein C-terminal helicase 1 [Source:HGNC Symbol;Acc:20473] |
| RTP4 | ENSG00000136514 | receptor (chemosensory) transporter protein 4 [Source:HGNC Symbol;Acc:23992] |
| IL36G | ENSG00000136688 | interleukin 36, gamma [Source:HGNC Symbol;Acc:15741] |
| IL1RN | ENSG00000136689 | interleukin 1 receptor antagonist [Source:HGNC Symbol;Acc:6000] |
| IL36A | ENSG00000136694 | interleukin 36, alpha [Source:HGNC Symbol;Acc:15562] |
| IL36RN | ENSG00000136695 | interleukin 36 receptor antagonist [Source:HGNC Symbol;Acc:15561] |
| TOR1B | ENSG00000136816 | torsin family 1, member B (torsin B) [Source:HGNC Symbol;Acc:11995] |
| DAB2IP | ENSG00000136848 | DAB2 interacting protein [Source:HGNC Symbol;Acc:17294] |
| TLR4 | ENSG00000136869 | toll-like receptor 4 [Source:HGNC Symbol;Acc:11850] |
| ALDOB | ENSG00000136872 | aldolase B, fructose-bisphosphate [Source:HGNC Symbol;Acc:417] |
| STX17 | ENSG00000136874 | syntaxin 17 [Source:HGNC Symbol;Acc:11432] |
| NOV | ENSG00000136999 | nephroblastoma overexpressed [Source:HGNC Symbol;Acc:7885] |
| IL33 | ENSG00000137033 | interleukin 33 [Source:HGNC Symbol;Acc:16028] |
| GMPR | ENSG00000137198 | guanosine monophosphate reductase [Source:HGNC Symbol;Acc:4376] |
| CMTR1 | ENSG00000137200 | cap methyltransferase 1 [Source:HGNC Symbol;Acc:21077] |
| IRF4 | ENSG00000137265 | interferon regulatory factor 4 [Source:HGNC Symbol;Acc:6119] |
| TUBB2A | ENSG00000137267 | tubulin, beta 2A class IIa [Source:HGNC Symbol;Acc:12412] |
| TUBB2B | ENSG00000137285 | tubulin, beta 2B class IIb [Source:HGNC Symbol;Acc:30829] |
| FGFBP1 | ENSG00000137440 | fibroblast growth factor binding protein 1 [Source:HGNC Symbol;Acc:19695] |
| CPEB2 | ENSG00000137449 | cytoplasmic polyadenylation element binding protein 2 [Source:HGNC Symbol;Acc:21745] |
| TLR2 | ENSG00000137462 | toll-like receptor 2 [Source:HGNC Symbol;Acc:11848] |
| FCHSD2 | ENSG00000137478 | FCH and double SH3 domains 2 [Source:HGNC Symbol;Acc:29114] |
| IL18BP | ENSG00000137496 | interleukin 18 binding protein [Source:HGNC Symbol;Acc:5987] |
| SULF1 | ENSG00000137573 | sulfatase 1 [Source:HGNC Symbol;Acc:20391] |
| DDX60 | ENSG00000137628 | DEAD (Asp-Glu-Ala-Asp) box polypeptide 60 [Source:HGNC Symbol;Acc:25942] |
| TMPRSS4 | ENSG00000137648 | transmembrane protease, serine 4 [Source:HGNC Symbol;Acc:11878] |
| MMP7 | ENSG00000137673 | matrix metallopeptidase 7 (matrilysin, uterine) [Source:HGNC Symbol;Acc:7174] |
| POU2F3 | ENSG00000137709 | POU class 2 homeobox 3 [Source:HGNC Symbol;Acc:19864] |
| FXYD6 | ENSG00000137726 | FXYD domain containing ion transport regulator 6 [Source:HGNC Symbol;Acc:4030] |
| ARHGAP20 | ENSG00000137727 | Rho GTPase activating protein 20 [Source:HGNC Symbol;Acc:18357] |
| MMP13 | ENSG00000137745 | matrix metallopeptidase 13 (collagenase 3) [Source:HGNC Symbol;Acc:7159] |
| CASP1 | ENSG00000137752 | caspase 1, apoptosis-related cysteine peptidase [Source:HGNC Symbol;Acc:1499] |
| SQRDL | ENSG00000137767 | sulfide quinone reductase-like (yeast) [Source:HGNC Symbol;Acc:20390] |
| TMEM62 | ENSG00000137842 | transmembrane protein 62 [Source:HGNC Symbol;Acc:26269] |
| IFI44L | ENSG00000137959 | interferon-induced protein 44-like [Source:HGNC Symbol;Acc:17817] |
| IFI44 | ENSG00000137965 | interferon-induced protein 44 [Source:HGNC Symbol;Acc:16938] |
| CLCA2 | ENSG00000137975 | chloride channel accessory 2 [Source:HGNC Symbol;Acc:2016] |
| PNPT1 | ENSG00000138035 | polyribonucleotide nucleotidyltransferase 1 [Source:HGNC Symbol;Acc:23166] |
| CYP2C9 | ENSG00000138109 | cytochrome P450, family 2, subfamily C, polypeptide 9 [Source:HGNC Symbol;Acc:2623] |
| CH25H | ENSG00000138135 | cholesterol 25-hydroxylase [Source:HGNC Symbol;Acc:1907] |
| DUSP5 | ENSG00000138166 | dual specificity phosphatase 5 [Source:HGNC Symbol;Acc:3071] |
| ADAMTS14 | ENSG00000138316 | ADAM metallopeptidase with thrombospondin type 1 motif, 14 [Source:HGNC Symbol;Acc:14899] |
| CHRNA1 | ENSG00000138435 | cholinergic receptor, nicotinic, alpha 1 (muscle) [Source:HGNC Symbol;Acc:1955] |
| PARP9 | ENSG00000138496 | poly (ADP-ribose) polymerase family, member 9 [Source:HGNC Symbol;Acc:24118] |
| HERC6 | ENSG00000138642 | HECT and RLD domain containing E3 ubiquitin protein ligase family member 6 [Source:HGNC Symbol;Acc:26072] |
| HERC5 | ENSG00000138646 | HECT and RLD domain containing E3 ubiquitin protein ligase 5 [Source:HGNC Symbol;Acc:24368] |
| RASGEF1B | ENSG00000138670 | RasGEF domain family, member 1B [Source:HGNC Symbol;Acc:24881] |
| FGF2 | ENSG00000138685 | fibroblast growth factor 2 (basic) [Source:HGNC Symbol;Acc:3676] |
| CXCL9 | ENSG00000138755 | chemokine (C-X-C motif) ligand 9 [Source:HGNC Symbol;Acc:7098] |
| SCARB2 | ENSG00000138760 | scavenger receptor class B, member 2 [Source:HGNC Symbol;Acc:1665] |
| INTS12 | ENSG00000138785 | integrator complex subunit 12 [Source:HGNC Symbol;Acc:25067] |
| EGF | ENSG00000138798 | epidermal growth factor [Source:HGNC Symbol;Acc:3229] |
| CPNE8 | ENSG00000139117 | copine VIII [Source:HGNC Symbol;Acc:23498] |
| NDUFA9 | ENSG00000139180 | NADH dehydrogenase (ubiquinone) 1 alpha subcomplex, 9, 39kDa [Source:HGNC Symbol;Acc:7693] |
| TAPBPL | ENSG00000139192 | TAP binding protein-like [Source:HGNC Symbol;Acc:30683] |
| SLC38A4 | ENSG00000139209 | solute carrier family 38, member 4 [Source:HGNC Symbol;Acc:14679] |
| GLIPR1 | ENSG00000139278 | GLI pathogenesis-related 1 [Source:HGNC Symbol;Acc:17001] |
| SDSL | ENSG00000139410 | serine dehydratase-like [Source:HGNC Symbol;Acc:30404] |
| TCHP | ENSG00000139437 | trichoplein, keratin filament binding [Source:HGNC Symbol;Acc:28135] |
| ACVRL1 | ENSG00000139567 | activin A receptor type II-like 1 [Source:HGNC Symbol;Acc:175] |
| N4BP2L1 | ENSG00000139597 | NEDD4 binding protein 2-like 1 [Source:HGNC Symbol;Acc:25037] |
| BRCA2 | ENSG00000139618 | breast cancer 2, early onset [Source:HGNC Symbol;Acc:1101] |
| LPAR6 | ENSG00000139679 | lysophosphatidic acid receptor 6 [Source:HGNC Symbol;Acc:15520] |
| CBLN3 | ENSG00000139899 | cerebellin 3 precursor [Source:HGNC Symbol;Acc:20146] |
| WARS | ENSG00000140105 | tryptophanyl-tRNA synthetase [Source:HGNC Symbol;Acc:12729] |
| DUOXA2 | ENSG00000140274 | dual oxidase maturation factor 2 [Source:HGNC Symbol;Acc:32698] |
| DUOX2 | ENSG00000140279 | dual oxidase 2 [Source:HGNC Symbol;Acc:13273] |
| LYSMD2 | ENSG00000140280 | LysM, putative peptidoglycan-binding, domain containing 2 [Source:HGNC Symbol;Acc:28571] |
| BCL2A1 | ENSG00000140379 | BCL2-related protein A1 [Source:HGNC Symbol;Acc:991] |
| PML | ENSG00000140464 | promyelocytic leukemia [Source:HGNC Symbol;Acc:9113] |
| HAPLN3 | ENSG00000140511 | hyaluronan and proteoglycan link protein 3 [Source:HGNC Symbol;Acc:21446] |
| CRTC3 | ENSG00000140577 | CREB regulated transcription coactivator 3 [Source:HGNC Symbol;Acc:26148] |
| CHST4 | ENSG00000140835 | carbohydrate (N-acetylglucosamine 6-O) sulfotransferase 4 [Source:HGNC Symbol;Acc:1972] |
| NLRC5 | ENSG00000140853 | NLR family, CARD domain containing 5 [Source:HGNC Symbol;Acc:29933] |
| OSGIN1 | ENSG00000140961 | oxidative stress induced growth inhibitor 1 [Source:HGNC Symbol;Acc:30093] |
| SECTM1 | ENSG00000141574 | secreted and transmembrane 1 [Source:HGNC Symbol;Acc:10707] |
| CBX4 | ENSG00000141582 | chromobox homolog 4 [Source:HGNC Symbol;Acc:1554] |
| TNFRSF11A | ENSG00000141655 | tumor necrosis factor receptor superfamily, member 11a, NFKB activator [Source:HGNC Symbol;Acc:11908] |
| ZCCHC2 | ENSG00000141664 | zinc finger, CCHC domain containing 2 [Source:HGNC Symbol;Acc:22916] |
| PMAIP1 | ENSG00000141682 | phorbol-12-myristate-13-acetate-induced protein 1 [Source:HGNC Symbol;Acc:9108] |
| IGFBP4 | ENSG00000141753 | insulin-like growth factor binding protein 4 [Source:HGNC Symbol;Acc:5473] |
| CACNA1A | ENSG00000141837 | calcium channel, voltage-dependent, P/Q type, alpha 1A subunit [Source:HGNC Symbol;Acc:1388] |
| TMEM91 | ENSG00000142046 | transmembrane protein 91 [Source:HGNC Symbol;Acc:32393] |
| IFITM3 | ENSG00000142089 | interferon induced transmembrane protein 3 [Source:HGNC Symbol;Acc:5414] |
| ATHL1 | ENSG00000142102 | ATH1, acid trehalase-like 1 (yeast) [Source:HGNC Symbol;Acc:26210] |
| COL6A1 | ENSG00000142156 | collagen, type VI, alpha 1 [Source:HGNC Symbol;Acc:2211] |
| IL19 | ENSG00000142224 | interleukin 19 [Source:HGNC Symbol;Acc:5990] |
| ERVK3-1 | ENSG00000142396 | endogenous retrovirus group K3, member 1 [Source:HGNC Symbol;Acc:30466] |
| CACNG8 | ENSG00000142408 | calcium channel, voltage-dependent, gamma subunit 8 [Source:HGNC Symbol;Acc:13628] |
| PRDM16 | ENSG00000142611 | PR domain containing 16 [Source:HGNC Symbol;Acc:14000] |
| EPHA2 | ENSG00000142627 | EPH receptor A2 [Source:HGNC Symbol;Acc:3386] |
| IL22RA1 | ENSG00000142677 | interleukin 22 receptor, alpha 1 [Source:HGNC Symbol;Acc:13700] |
| MAP3K6 | ENSG00000142733 | mitogen-activated protein kinase kinase kinase 6 [Source:HGNC Symbol;Acc:6858] |
| ADC | ENSG00000142920 | arginine decarboxylase [Source:HGNC Symbol;Acc:29957] |
| MOB3C | ENSG00000142961 | MOB kinase activator 3C [Source:HGNC Symbol;Acc:29800] |
| ATP1B1 | ENSG00000143153 | ATPase, Na+/K+ transporting, beta 1 polypeptide [Source:HGNC Symbol;Acc:804] |
| FCGR2A | ENSG00000143226 | Fc fragment of IgG, low affinity IIa, receptor (CD32) [Source:HGNC Symbol;Acc:3616] |
| RGL1 | ENSG00000143344 | ral guanine nucleotide dissociation stimulator-like 1 [Source:HGNC Symbol;Acc:30281] |
| ADAMTSL4 | ENSG00000143382 | ADAMTS-like 4 [Source:HGNC Symbol;Acc:19706] |
| MCL1 | ENSG00000143384 | myeloid cell leukemia sequence 1 (BCL2-related) [Source:HGNC Symbol;Acc:6943] |
| RFX5 | ENSG00000143390 | regulatory factor X, 5 (influences HLA class II expression) [Source:HGNC Symbol;Acc:9986] |
| IKBKE | ENSG00000143466 | inhibitor of kappa light polypeptide gene enhancer in B-cells, kinase epsilon [Source:HGNC Symbol;Acc:14552] |
| KCNN3 | ENSG00000143603 | potassium intermediate/small conductance calcium-activated channel, subfamily N, member 3 [Source:HGNC Symbol;Acc:6292] |
| GALM | ENSG00000143891 | galactose mutarotase (aldose 1-epimerase) [Source:HGNC Symbol;Acc:24063] |
| NAT8 | ENSG00000144035 | N-acetyltransferase 8 (GCN5-related, putative) [Source:HGNC Symbol;Acc:18069] |
| MALL | ENSG00000144063 | mal, T-cell differentiation protein-like [Source:HGNC Symbol;Acc:6818] |
| ABCA12 | ENSG00000144452 | ATP-binding cassette, sub-family A (ABC1), member 12 [Source:HGNC Symbol;Acc:14637] |
| ACKR3 | ENSG00000144476 | atypical chemokine receptor 3 [Source:HGNC Symbol;Acc:23692] |
| GRIP2 | ENSG00000144596 | glutamate receptor interacting protein 2 [Source:HGNC Symbol;Acc:23841] |
| ACKR2 | ENSG00000144648 | atypical chemokine receptor 2 [Source:HGNC Symbol;Acc:1565] |
| CSRNP1 | ENSG00000144655 | cysteine-serine-rich nuclear protein 1 [Source:HGNC Symbol;Acc:14300] |
| STAC | ENSG00000144681 | SH3 and cysteine rich domain [Source:HGNC Symbol;Acc:11353] |
| NFKBIZ | ENSG00000144802 | nuclear factor of kappa light polypeptide gene enhancer in B-cells inhibitor, zeta [Source:HGNC Symbol;Acc:29805] |
| COL8A1 | ENSG00000144810 | collagen, type VIII, alpha 1 [Source:HGNC Symbol;Acc:2215] |
| ADPRH | ENSG00000144843 | ADP-ribosylarginine hydrolase [Source:HGNC Symbol;Acc:269] |
| IGSF11 | ENSG00000144847 | immunoglobulin superfamily, member 11 [Source:HGNC Symbol;Acc:16669] |
| NCEH1 | ENSG00000144959 | neutral cholesterol ester hydrolase 1 [Source:HGNC Symbol;Acc:29260] |
| KIAA0226 | ENSG00000145016 | KIAA0226 [Source:HGNC Symbol;Acc:28991] |
| ILDR1 | ENSG00000145103 | immunoglobulin-like domain containing receptor 1 [Source:HGNC Symbol;Acc:28741] |
| MUC4 | ENSG00000145113 | mucin 4, cell surface associated [Source:HGNC Symbol;Acc:7514] |
| PLAC8 | ENSG00000145287 | placenta-specific 8 [Source:HGNC Symbol;Acc:19254] |
| DDIT4L | ENSG00000145358 | DNA-damage-inducible transcript 4-like [Source:HGNC Symbol;Acc:30555] |
| RNF175 | ENSG00000145428 | ring finger protein 175 [Source:HGNC Symbol;Acc:27735] |
| TNFAIP8 | ENSG00000145779 | tumor necrosis factor, alpha-induced protein 8 [Source:HGNC Symbol;Acc:17260] |
| ARHGAP26 | ENSG00000145819 | Rho GTPase activating protein 26 [Source:HGNC Symbol;Acc:17073] |
| FGD2 | ENSG00000146192 | FYVE, RhoGEF and PH domain containing 2 [Source:HGNC Symbol;Acc:3664] |
| SCUBE3 | ENSG00000146197 | signal peptide, CUB domain, EGF-like 3 [Source:HGNC Symbol;Acc:13655] |
| SLC18B1 | ENSG00000146409 | solute carrier family 18, subfamily B, member 1 [Source:HGNC Symbol;Acc:21573] |
| SLC2A12 | ENSG00000146411 | solute carrier family 2 (facilitated glucose transporter), member 12 [Source:HGNC Symbol;Acc:18067] |
| SDK1 | ENSG00000146555 | sidekick cell adhesion molecule 1 [Source:HGNC Symbol;Acc:19307] |
| ATXN7L1 | ENSG00000146776 | ataxin 7-like 1 [Source:HGNC Symbol;Acc:22210] |
| TMEM140 | ENSG00000146859 | transmembrane protein 140 [Source:HGNC Symbol;Acc:21870] |
| RAB19 | ENSG00000146955 | RAB19, member RAS oncogene family [Source:HGNC Symbol;Acc:19982] |
| TMEM27 | ENSG00000147003 | transmembrane protein 27 [Source:HGNC Symbol;Acc:29437] |
| SH3KBP1 | ENSG00000147010 | SH3-domain kinase binding protein 1 [Source:HGNC Symbol;Acc:13867] |
| SYTL5 | ENSG00000147041 | synaptotagmin-like 5 [Source:HGNC Symbol;Acc:15589] |
| AWAT2 | ENSG00000147160 | acyl-CoA wax alcohol acyltransferase 2 [Source:HGNC Symbol;Acc:23251] |
| IL2RG | ENSG00000147168 | interleukin 2 receptor, gamma [Source:HGNC Symbol;Acc:6010] |
| GSDMC | ENSG00000147697 | gasdermin C [Source:HGNC Symbol;Acc:7151] |
| PTGES | ENSG00000148344 | prostaglandin E synthase [Source:HGNC Symbol;Acc:9599] |
| LCN2 | ENSG00000148346 | lipocalin 2 [Source:HGNC Symbol;Acc:6526] |
| HMCN2 | ENSG00000148357 | hemicentin 2 [Source:HGNC Symbol;Acc:21293] |
| ADD3 | ENSG00000148700 | adducin 3 (gamma) [Source:HGNC Symbol;Acc:245] |
| INA | ENSG00000148798 | internexin neuronal intermediate filament protein, alpha [Source:HGNC Symbol;Acc:6057] |
| PAOX | ENSG00000148832 | polyamine oxidase (exo-N4-amino) [Source:HGNC Symbol;Acc:20837] |
| ADM | ENSG00000148926 | adrenomedullin [Source:HGNC Symbol;Acc:259] |
| LRRC4C | ENSG00000148948 | leucine rich repeat containing 4C [Source:HGNC Symbol;Acc:29317] |
| SAA4 | ENSG00000148965 | serum amyloid A4, constitutive [Source:HGNC Symbol;Acc:10516] |
| SERPING1 | ENSG00000149131 | serpin peptidase inhibitor, clade G (C1 inhibitor), member 1 [Source:HGNC Symbol;Acc:1228] |
| ENDOD1 | ENSG00000149218 | endonuclease domain containing 1 [Source:HGNC Symbol;Acc:29129] |
| TENM4 | ENSG00000149256 | teneurin transmembrane protein 4 [Source:HGNC Symbol;Acc:29945] |
| CAPN5 | ENSG00000149260 | calpain 5 [Source:HGNC Symbol;Acc:1482] |
| ZC3H12C | ENSG00000149289 | zinc finger CCCH-type containing 12C [Source:HGNC Symbol;Acc:29362] |
| HTR3B | ENSG00000149305 | 5-hydroxytryptamine (serotonin) receptor 3B, ionotropic [Source:HGNC Symbol;Acc:5298] |
| ST14 | ENSG00000149418 | suppression of tumorigenicity 14 (colon carcinoma) [Source:HGNC Symbol;Acc:11344] |
| DUSP15 | ENSG00000149599 | dual specificity phosphatase 15 [Source:HGNC Symbol;Acc:16236] |
| CDC42EP2 | ENSG00000149798 | CDC42 effector protein (Rho GTPase binding) 2 [Source:HGNC Symbol;Acc:16263] |
| KLRF1 | ENSG00000150045 | killer cell lectin-like receptor subfamily F, member 1 [Source:HGNC Symbol;Acc:13342] |
| CLEC1A | ENSG00000150048 | C-type lectin domain family 1, member A [Source:HGNC Symbol;Acc:24355] |
| ANXA8L1 | ENSG00000150165 | annexin A8-like 1 [Source:HGNC Symbol;Acc:23334] |
| ARID5B | ENSG00000150347 | AT rich interactive domain 5B (MRF1-like) [Source:HGNC Symbol;Acc:17362] |
| CDH8 | ENSG00000150394 | cadherin 8, type 2 [Source:HGNC Symbol;Acc:1767] |
| PRSS23 | ENSG00000150687 | protease, serine, 23 [Source:HGNC Symbol;Acc:14370] |
| SLC7A11 | ENSG00000151012 | solute carrier family 7 (anionic amino acid transporter light chain, xc- system), member 11 [Source:HGNC Symbol;Acc:11059] |
| CCRN4L | ENSG00000151014 | CCR4 carbon catabolite repression 4-like (S. cerevisiae) [Source:HGNC Symbol;Acc:14254] |
| BTBD11 | ENSG00000151136 | BTB (POZ) domain containing 11 [Source:HGNC Symbol;Acc:23844] |
| DIP2C | ENSG00000151240 | DIP2 disco-interacting protein 2 homolog C (Drosophila) [Source:HGNC Symbol;Acc:29150] |
| SCLT1 | ENSG00000151466 | sodium channel and clathrin linker 1 [Source:HGNC Symbol;Acc:26406] |
| CCDC3 | ENSG00000151468 | coiled-coil domain containing 3 [Source:HGNC Symbol;Acc:23813] |
| C4orf33 | ENSG00000151470 | chromosome 4 open reading frame 33 [Source:HGNC Symbol;Acc:27025] |
| FRMD4A | ENSG00000151474 | FERM domain containing 4A [Source:HGNC Symbol;Acc:25491] |
| EDNRA | ENSG00000151617 | endothelin receptor type A [Source:HGNC Symbol;Acc:3179] |
| ADAM8 | ENSG00000151651 | ADAM metallopeptidase domain 8 [Source:HGNC Symbol;Acc:215] |
| RNF144A | ENSG00000151692 | ring finger protein 144A [Source:HGNC Symbol;Acc:20457] |
| PARP8 | ENSG00000151883 | poly (ADP-ribose) polymerase family, member 8 [Source:HGNC Symbol;Acc:26124] |
| RABGAP1L | ENSG00000152061 | RAB GTPase activating protein 1-like [Source:HGNC Symbol;Acc:24663] |
| CWF19L2 | ENSG00000152404 | CWF19-like 2, cell cycle control (S. pombe) [Source:HGNC Symbol;Acc:26508] |
| TRIM36 | ENSG00000152503 | tripartite motif containing 36 [Source:HGNC Symbol;Acc:16280] |
| ZFP36L2 | ENSG00000152518 | ZFP36 ring finger protein-like 2 [Source:HGNC Symbol;Acc:1108] |
| SPARCL1 | ENSG00000152583 | SPARC-like 1 (hevin) [Source:HGNC Symbol;Acc:11220] |
| RASGRP3 | ENSG00000152689 | RAS guanyl releasing protein 3 (calcium and DAG-regulated) [Source:HGNC Symbol;Acc:14545] |
| GPR180 | ENSG00000152749 | G protein-coupled receptor 180 [Source:HGNC Symbol;Acc:28899] |
| IFIT5 | ENSG00000152778 | interferon-induced protein with tetratricopeptide repeats 5 [Source:HGNC Symbol;Acc:13328] |
| BMP3 | ENSG00000152785 | bone morphogenetic protein 3 [Source:HGNC Symbol;Acc:1070] |
| BANK1 | ENSG00000153064 | B-cell scaffold protein with ankyrin repeats 1 [Source:HGNC Symbol;Acc:18233] |
| PTPRR | ENSG00000153233 | protein tyrosine phosphatase, receptor type, R [Source:HGNC Symbol;Acc:9680] |
| NR4A2 | ENSG00000153234 | nuclear receptor subfamily 4, group A, member 2 [Source:HGNC Symbol;Acc:7981] |
| TMPRSS11D | ENSG00000153802 | transmembrane protease, serine 11D [Source:HGNC Symbol;Acc:24059] |
| CLCA3P | ENSG00000153923 | chloride channel accessory 3, pseudogene [Source:HGNC Symbol;Acc:2017] |
| SEMA3D | ENSG00000153993 | sema domain, immunoglobulin domain (Ig), short basic domain, secreted, (semaphorin) 3D [Source:HGNC Symbol;Acc:10726] |
| ANGPT1 | ENSG00000154188 | angiopoietin 1 [Source:HGNC Symbol;Acc:484] |
| CERS3 | ENSG00000154227 | ceramide synthase 3 [Source:HGNC Symbol;Acc:23752] |
| ABCA6 | ENSG00000154262 | ATP-binding cassette, sub-family A (ABC1), member 6 [Source:HGNC Symbol;Acc:36] |
| C4orf19 | ENSG00000154274 | chromosome 4 open reading frame 19 [Source:HGNC Symbol;Acc:25618] |
| GBP5 | ENSG00000154451 | guanylate binding protein 5 [Source:HGNC Symbol;Acc:19895] |
| C21orf91 | ENSG00000154642 | chromosome 21 open reading frame 91 [Source:HGNC Symbol;Acc:16459] |
| CHODL | ENSG00000154645 | chondrolectin [Source:HGNC Symbol;Acc:17807] |
| NCAM2 | ENSG00000154654 | neural cell adhesion molecule 2 [Source:HGNC Symbol;Acc:7657] |
| L3MBTL4 | ENSG00000154655 | l(3)mbt-like 4 (Drosophila) [Source:HGNC Symbol;Acc:26677] |
| PDE1C | ENSG00000154678 | phosphodiesterase 1C, calmodulin-dependent 70kDa [Source:HGNC Symbol;Acc:8776] |
| APCDD1 | ENSG00000154856 | adenomatosis polyposis coli down-regulated 1 [Source:HGNC Symbol;Acc:15718] |
| UNC93B2 | ENSG00000155070 | unc-93 homolog B2 pseudogene (C. elegans) [Source:HGNC Symbol;Acc:13482] |
| TTC39B | ENSG00000155158 | tetratricopeptide repeat domain 39B [Source:HGNC Symbol;Acc:23704] |
| SLC25A28 | ENSG00000155287 | solute carrier family 25 (mitochondrial iron transporter), member 28 [Source:HGNC Symbol;Acc:23472] |
| MOV10 | ENSG00000155363 | Mov10, Moloney leukemia virus 10, homolog (mouse) [Source:HGNC Symbol;Acc:7200] |
| PPM1J | ENSG00000155367 | protein phosphatase, Mg2+/Mn2+ dependent, 1J [Source:HGNC Symbol;Acc:20785] |
| SLC16A1 | ENSG00000155380 | solute carrier family 16 (monocarboxylate transporter), member 1 [Source:HGNC Symbol;Acc:10922] |
| SLC7A7 | ENSG00000155465 | solute carrier family 7 (amino acid transporter light chain, y+L system), member 7 [Source:HGNC Symbol;Acc:11065] |
| PIK3AP1 | ENSG00000155629 | phosphoinositide-3-kinase adaptor protein 1 [Source:HGNC Symbol;Acc:30034] |
| TTN | ENSG00000155657 | titin [Source:HGNC Symbol;Acc:12403] |
| PPARGC1B | ENSG00000155846 | peroxisome proliferator-activated receptor gamma, coactivator 1 beta [Source:HGNC Symbol;Acc:30022] |
| RAET1L | ENSG00000155918 | retinoic acid early transcript 1L [Source:HGNC Symbol;Acc:16798] |
| GNA14 | ENSG00000156049 | guanine nucleotide binding protein (G protein), alpha 14 [Source:HGNC Symbol;Acc:4382] |
| BATF | ENSG00000156127 | basic leucine zipper transcription factor, ATF-like [Source:HGNC Symbol;Acc:958] |
| ADAMTS3 | ENSG00000156140 | ADAM metallopeptidase with thrombospondin type 1 motif, 3 [Source:HGNC Symbol;Acc:219] |
| ADAMTSL3 | ENSG00000156218 | ADAMTS-like 3 [Source:HGNC Symbol;Acc:14633] |
| ART3 | ENSG00000156219 | ADP-ribosyltransferase 3 [Source:HGNC Symbol;Acc:725] |
| CXCL13 | ENSG00000156234 | chemokine (C-X-C motif) ligand 13 [Source:HGNC Symbol;Acc:10639] |
| FUT6 | ENSG00000156413 | fucosyltransferase 6 (alpha (1,3) fucosyltransferase) [Source:HGNC Symbol;Acc:4017] |
| TDRD9 | ENSG00000156414 | tudor domain containing 9 [Source:HGNC Symbol;Acc:20122] |
| FAM122C | ENSG00000156500 | family with sequence similarity 122C [Source:HGNC Symbol;Acc:25202] |
| UBE2L6 | ENSG00000156587 | ubiquitin-conjugating enzyme E2L 6 [Source:HGNC Symbol;Acc:12490] |
| B3GNT7 | ENSG00000156966 | UDP-GlcNAc:betaGal beta-1,3-N-acetylglucosaminyltransferase 7 [Source:HGNC Symbol;Acc:18811] |
| TMEM171 | ENSG00000157111 | transmembrane protein 171 [Source:HGNC Symbol;Acc:27031] |
| LRP8 | ENSG00000157193 | low density lipoprotein receptor-related protein 8, apolipoprotein e receptor [Source:HGNC Symbol;Acc:6700] |
| ERG | ENSG00000157554 | v-ets avian erythroblastosis virus E26 oncogene homolog [Source:HGNC Symbol;Acc:3446] |
| MX1 | ENSG00000157601 | myxovirus (influenza virus) resistance 1, interferon-inducible protein p78 (mouse) [Source:HGNC Symbol;Acc:7532] |
| CREB3L1 | ENSG00000157613 | cAMP responsive element binding protein 3-like 1 [Source:HGNC Symbol;Acc:18856] |
| ZNF618 | ENSG00000157657 | zinc finger protein 618 [Source:HGNC Symbol;Acc:29416] |
| C9orf91 | ENSG00000157693 | chromosome 9 open reading frame 91 [Source:HGNC Symbol;Acc:24513] |
| SLC34A2 | ENSG00000157765 | solute carrier family 34 (type II sodium/phosphate contransporter), member 2 [Source:HGNC Symbol;Acc:11020] |
| FAM213B | ENSG00000157870 | family with sequence similarity 213, member B [Source:HGNC Symbol;Acc:28390] |
| TNFRSF14 | ENSG00000157873 | tumor necrosis factor receptor superfamily, member 14 [Source:HGNC Symbol;Acc:11912] |
| SLC30A2 | ENSG00000158014 | solute carrier family 30 (zinc transporter), member 2 [Source:HGNC Symbol;Acc:11013] |
| DUSP2 | ENSG00000158050 | dual specificity phosphatase 2 [Source:HGNC Symbol;Acc:3068] |
| XDH | ENSG00000158125 | xanthine dehydrogenase [Source:HGNC Symbol;Acc:12805] |
| HIST1H2BD | ENSG00000158373 | histone cluster 1, H2bd [Source:HGNC Symbol;Acc:4747] |
| TSPAN33 | ENSG00000158457 | tetraspanin 33 [Source:HGNC Symbol;Acc:28743] |
| NRG2 | ENSG00000158458 | neuregulin 2 [Source:HGNC Symbol;Acc:7998] |
| B4GALT5 | ENSG00000158470 | UDP-Gal:betaGlcNAc beta 1,4- galactosyltransferase, polypeptide 5 [Source:HGNC Symbol;Acc:928] |
| NCF1 | ENSG00000158517 | neutrophil cytosolic factor 1 [Source:HGNC Symbol;Acc:7660] |
| PKD1L1 | ENSG00000158683 | polycystic kidney disease 1 like 1 [Source:HGNC Symbol;Acc:18053] |
| SYNJ1 | ENSG00000159082 | synaptojanin 1 [Source:HGNC Symbol;Acc:11503] |
| IFNGR2 | ENSG00000159128 | interferon gamma receptor 2 (interferon gamma transducer 1) [Source:HGNC Symbol;Acc:5440] |
| CBR3 | ENSG00000159231 | carbonyl reductase 3 [Source:HGNC Symbol;Acc:1549] |
| ARHGAP27 | ENSG00000159314 | Rho GTPase activating protein 27 [Source:HGNC Symbol;Acc:31813] |
| C1R | ENSG00000159403 | complement component 1, r subcomponent [Source:HGNC Symbol;Acc:1246] |
| SPRR2G | ENSG00000159516 | small proline-rich protein 2G [Source:HGNC Symbol;Acc:11267] |
| PGLYRP3 | ENSG00000159527 | peptidoglycan recognition protein 3 [Source:HGNC Symbol;Acc:30014] |
| LYPD5 | ENSG00000159871 | LY6/PLAUR domain containing 5 [Source:HGNC Symbol;Acc:26397] |
| GNE | ENSG00000159921 | glucosamine (UDP-N-acetyl)-2-epimerase/N-acetylmannosamine kinase [Source:HGNC Symbol;Acc:23657] |
| CPAMD8 | ENSG00000160111 | C3 and PZP-like, alpha-2-macroglobulin domain containing 8 [Source:HGNC Symbol;Acc:23228] |
| TMPRSS3 | ENSG00000160183 | transmembrane protease, serine 3 [Source:HGNC Symbol;Acc:11877] |
| CBS | ENSG00000160200 | cystathionine-beta-synthase [Source:HGNC Symbol;Acc:1550] |
| ICOSLG | ENSG00000160223 | inducible T-cell co-stimulator ligand [Source:HGNC Symbol;Acc:17087] |
| LRRC3 | ENSG00000160233 | leucine rich repeat containing 3 [Source:HGNC Symbol;Acc:14965] |
| LSS | ENSG00000160285 | lanosterol synthase (2,3-oxidosqualene-lanosterol cyclase) [Source:HGNC Symbol;Acc:6708] |
| SLC2A6 | ENSG00000160326 | solute carrier family 2 (facilitated glucose transporter), member 6 [Source:HGNC Symbol;Acc:11011] |
| RDH13 | ENSG00000160439 | retinol dehydrogenase 13 (all-trans/9-cis) [Source:HGNC Symbol;Acc:19978] |
| ADAR | ENSG00000160710 | adenosine deaminase, RNA-specific [Source:HGNC Symbol;Acc:225] |
| LY6E | ENSG00000160932 | lymphocyte antigen 6 complex, locus E [Source:HGNC Symbol;Acc:6727] |
| SCGB3A1 | ENSG00000161055 | secretoglobin, family 3A, member 1 [Source:HGNC Symbol;Acc:18384] |
| MFSD12 | ENSG00000161091 | major facilitator superfamily domain containing 12 [Source:HGNC Symbol;Acc:28299] |
| USP41 | ENSG00000161133 | ubiquitin specific peptidase 41 [Source:HGNC Symbol;Acc:20070] |
| CCL5 | ENSG00000161570 | chemokine (C-C motif) ligand 5 [Source:HGNC Symbol;Acc:10632] |
| DBF4B | ENSG00000161692 | DBF4 homolog B (S. cerevisiae) [Source:HGNC Symbol;Acc:17883] |
| AQP5 | ENSG00000161798 | aquaporin 5 [Source:HGNC Symbol;Acc:638] |
| CXCL16 | ENSG00000161921 | chemokine (C-X-C motif) ligand 16 [Source:HGNC Symbol;Acc:16642] |
| TNFSF13 | ENSG00000161955 | tumor necrosis factor (ligand) superfamily, member 13 [Source:HGNC Symbol;Acc:11928] |
| ZG16B | ENSG00000162078 | zymogen granule protein 16B [Source:HGNC Symbol;Acc:30456] |
| PDZK1IP1 | ENSG00000162366 | PDZK1 interacting protein 1 [Source:HGNC Symbol;Acc:16887] |
| ACOT11 | ENSG00000162390 | acyl-CoA thioesterase 11 [Source:HGNC Symbol;Acc:18156] |
| PDPN | ENSG00000162493 | podoplanin [Source:HGNC Symbol;Acc:29602] |
| SYNC | ENSG00000162520 | syncoilin, intermediate filament protein [Source:HGNC Symbol;Acc:28897] |
| WNT4 | ENSG00000162552 | wingless-type MMTV integration site family, member 4 [Source:HGNC Symbol;Acc:12783] |
| NEXN | ENSG00000162614 | nexilin (F actin binding protein) [Source:HGNC Symbol;Acc:29557] |
| GBP2 | ENSG00000162645 | guanylate binding protein 2, interferon-inducible [Source:HGNC Symbol;Acc:4183] |
| GBP4 | ENSG00000162654 | guanylate binding protein 4 [Source:HGNC Symbol;Acc:20480] |
| VCAM1 | ENSG00000162692 | vascular cell adhesion molecule 1 [Source:HGNC Symbol;Acc:12663] |
| SLAMF9 | ENSG00000162723 | SLAM family member 9 [Source:HGNC Symbol;Acc:18430] |
| SLC9C2 | ENSG00000162753 | solute carrier family 9, member C2 (putative) [Source:HGNC Symbol;Acc:28664] |
| C1orf74 | ENSG00000162757 | chromosome 1 open reading frame 74 [Source:HGNC Symbol;Acc:26319] |
| MT2P1 | ENSG00000162840 | metallothionein 2 pseudogene 1 [Source:HGNC Symbol;Acc:7407] |
| KIF26B | ENSG00000162849 | kinesin family member 26B [Source:HGNC Symbol;Acc:25484] |
| KLHDC8A | ENSG00000162873 | kelch domain containing 8A [Source:HGNC Symbol;Acc:25573] |
| C1orf147 | ENSG00000162888 | chromosome 1 open reading frame 147 [Source:HGNC Symbol;Acc:32061] |
| PIGR | ENSG00000162896 | polymeric immunoglobulin receptor [Source:HGNC Symbol;Acc:8968] |
| ALMS1P | ENSG00000163016 | Alstrom syndrome 1 pseudogene [Source:HGNC Symbol;Acc:29586] |
| VSNL1 | ENSG00000163032 | visinin-like 1 [Source:HGNC Symbol;Acc:12722] |
| NEURL3 | ENSG00000163121 | neuralized E3 ubiquitin protein ligase 3 [Source:HGNC Symbol;Acc:25162] |
| CTSS | ENSG00000163131 | cathepsin S [Source:HGNC Symbol;Acc:2545] |
| SPRR2D | ENSG00000163216 | small proline-rich protein 2D [Source:HGNC Symbol;Acc:11264] |
| ARHGAP25 | ENSG00000163219 | Rho GTPase activating protein 25 [Source:HGNC Symbol;Acc:28951] |
| S100A9 | ENSG00000163220 | S100 calcium binding protein A9 [Source:HGNC Symbol;Acc:10499] |
| CLDN1 | ENSG00000163347 | claudin 1 [Source:HGNC Symbol;Acc:2032] |
| KBTBD8 | ENSG00000163376 | kelch repeat and BTB (POZ) domain containing 8 [Source:HGNC Symbol;Acc:30691] |
| FBLN2 | ENSG00000163520 | fibulin 2 [Source:HGNC Symbol;Acc:3601] |
| NFASC | ENSG00000163531 | neurofascin [Source:HGNC Symbol;Acc:29866] |
| NUAK2 | ENSG00000163545 | NUAK family, SNF1-like kinase, 2 [Source:HGNC Symbol;Acc:29558] |
| PYHIN1 | ENSG00000163564 | pyrin and HIN domain family, member 1 [Source:HGNC Symbol;Acc:28894] |
| IFI16 | ENSG00000163565 | interferon, gamma-inducible protein 16 [Source:HGNC Symbol;Acc:5395] |
| AIM2 | ENSG00000163568 | absent in melanoma 2 [Source:HGNC Symbol;Acc:357] |
| CD200R1 | ENSG00000163606 | CD200 receptor 1 [Source:HGNC Symbol;Acc:24235] |
| PPM1K | ENSG00000163644 | protein phosphatase, Mg2+/Mn2+ dependent, 1K [Source:HGNC Symbol;Acc:25415] |
| HESX1 | ENSG00000163666 | HESX homeobox 1 [Source:HGNC Symbol;Acc:4877] |
| CXCL3 | ENSG00000163734 | chemokine (C-X-C motif) ligand 3 [Source:HGNC Symbol;Acc:4604] |
| CXCL1 | ENSG00000163739 | chemokine (C-X-C motif) ligand 1 (melanoma growth stimulating activity, alpha) [Source:HGNC Symbol;Acc:4602] |
| PLSCR2 | ENSG00000163746 | phospholipid scramblase 2 [Source:HGNC Symbol;Acc:16494] |
| SLC6A20 | ENSG00000163817 | solute carrier family 6 (proline IMINO transporter), member 20 [Source:HGNC Symbol;Acc:30927] |
| CCR1 | ENSG00000163823 | chemokine (C-C motif) receptor 1 [Source:HGNC Symbol;Acc:1602] |
| DTX3L | ENSG00000163840 | deltex 3-like (Drosophila) [Source:HGNC Symbol;Acc:30323] |
| ZC3H12A | ENSG00000163874 | zinc finger CCCH-type containing 12A [Source:HGNC Symbol;Acc:26259] |
| LIPH | ENSG00000163898 | lipase, member H [Source:HGNC Symbol;Acc:18483] |
| SLC51A | ENSG00000163959 | solute carrier family 51, alpha subunit [Source:HGNC Symbol;Acc:29955] |
| S100P | ENSG00000163993 | S100 calcium binding protein P [Source:HGNC Symbol;Acc:10504] |
| SHISA5 | ENSG00000164054 | shisa family member 5 [Source:HGNC Symbol;Acc:30376] |
| MST1R | ENSG00000164078 | macrophage stimulating 1 receptor (c-met-related tyrosine kinase) [Source:HGNC Symbol;Acc:7381] |
| GUCY1A3 | ENSG00000164116 | guanylate cyclase 1, soluble, alpha 3 [Source:HGNC Symbol;Acc:4685] |
| IL15 | ENSG00000164136 | interleukin 15 [Source:HGNC Symbol;Acc:5977] |
| ELOVL7 | ENSG00000164181 | ELOVL fatty acid elongase 7 [Source:HGNC Symbol;Acc:26292] |
| GPR98 | ENSG00000164199 | G protein-coupled receptor 98 [Source:HGNC Symbol;Acc:17416] |
| STARD4 | ENSG00000164211 | StAR-related lipid transfer (START) domain containing 4 [Source:HGNC Symbol;Acc:18058] |
| ENPP6 | ENSG00000164303 | ectonucleotide pyrophosphatase/phosphodiesterase 6 [Source:HGNC Symbol;Acc:23409] |
| ERAP1 | ENSG00000164307 | endoplasmic reticulum aminopeptidase 1 [Source:HGNC Symbol;Acc:18173] |
| ERAP2 | ENSG00000164308 | endoplasmic reticulum aminopeptidase 2 [Source:HGNC Symbol;Acc:29499] |
| CMYA5 | ENSG00000164309 | cardiomyopathy associated 5 [Source:HGNC Symbol;Acc:14305] |
| EGFLAM | ENSG00000164318 | EGF-like, fibronectin type III and laminin G domains [Source:HGNC Symbol;Acc:26810] |
| RICTOR | ENSG00000164327 | RPTOR independent companion of MTOR, complex 2 [Source:HGNC Symbol;Acc:28611] |
| TLR3 | ENSG00000164342 | toll-like receptor 3 [Source:HGNC Symbol;Acc:11849] |
| GJB7 | ENSG00000164411 | gap junction protein, beta 7, 25kDa [Source:HGNC Symbol;Acc:16690] |
| MB21D1 | ENSG00000164430 | Mab-21 domain containing 1 [Source:HGNC Symbol;Acc:21367] |
| KCNK5 | ENSG00000164626 | potassium channel, subfamily K, member 5 [Source:HGNC Symbol;Acc:6280] |
| TAGAP | ENSG00000164691 | T-cell activation RhoGTPase activating protein [Source:HGNC Symbol;Acc:15669] |
| HNF4G | ENSG00000164749 | hepatocyte nuclear factor 4, gamma [Source:HGNC Symbol;Acc:5026] |
| DEFB1 | ENSG00000164825 | defensin, beta 1 [Source:HGNC Symbol;Acc:2766] |
| CA3 | ENSG00000164879 | carbonic anhydrase III, muscle specific [Source:HGNC Symbol;Acc:1374] |
| OSR2 | ENSG00000164920 | odd-skipped related transciption factor 2 [Source:HGNC Symbol;Acc:15830] |
| CPA6 | ENSG00000165078 | carboxypeptidase A6 [Source:HGNC Symbol;Acc:17245] |
| TMC1 | ENSG00000165091 | transmembrane channel-like 1 [Source:HGNC Symbol;Acc:16513] |
| NCF1C | ENSG00000165178 | neutrophil cytosolic factor 1C pseudogene [Source:HGNC Symbol;Acc:32523] |
| C9orf84 | ENSG00000165181 | chromosome 9 open reading frame 84 [Source:HGNC Symbol;Acc:26535] |
| RNF183 | ENSG00000165188 | ring finger protein 183 [Source:HGNC Symbol;Acc:28721] |
| FIGF | ENSG00000165197 | c-fos induced growth factor (vascular endothelial growth factor D) [Source:HGNC Symbol;Acc:3708] |
| HDX | ENSG00000165259 | highly divergent homeobox [Source:HGNC Symbol;Acc:26411] |
| ANXA8 | ENSG00000165390 | annexin A8 [Source:HGNC Symbol;Acc:546] |
| GJB2 | ENSG00000165474 | gap junction protein, beta 2, 26kDa [Source:HGNC Symbol;Acc:4284] |
| C11orf82 | ENSG00000165490 | chromosome 11 open reading frame 82 [Source:HGNC Symbol;Acc:26351] |
| RET | ENSG00000165731 | ret proto-oncogene [Source:HGNC Symbol;Acc:9967] |
| KIAA1462 | ENSG00000165757 | KIAA1462 [Source:HGNC Symbol;Acc:29283] |
| CASP7 | ENSG00000165806 | caspase 7, apoptosis-related cysteine peptidase [Source:HGNC Symbol;Acc:1508] |
| CYP2C19 | ENSG00000165841 | cytochrome P450, family 2, subfamily C, polypeptide 19 [Source:HGNC Symbol;Acc:2621] |
| SMCO2 | ENSG00000165935 | single-pass membrane protein with coiled-coil domains 2 [Source:HGNC Symbol;Acc:34448] |
| IFI27 | ENSG00000165949 | interferon, alpha-inducible protein 27 [Source:HGNC Symbol;Acc:5397] |
| ABTB2 | ENSG00000166016 | ankyrin repeat and BTB (POZ) domain containing 2 [Source:HGNC Symbol;Acc:23842] |
| LIPC | ENSG00000166035 | lipase, hepatic [Source:HGNC Symbol;Acc:6619] |
| JAM3 | ENSG00000166086 | junctional adhesion molecule 3 [Source:HGNC Symbol;Acc:15532] |
| GPT2 | ENSG00000166123 | glutamic pyruvate transaminase (alanine aminotransferase) 2 [Source:HGNC Symbol;Acc:18062] |
| PLEKHF1 | ENSG00000166289 | pleckstrin homology domain containing, family F (with FYVE domain) member 1 [Source:HGNC Symbol;Acc:20764] |
| MOGAT2 | ENSG00000166391 | monoacylglycerol O-acyltransferase 2 [Source:HGNC Symbol;Acc:23248] |
| SERPINB7 | ENSG00000166396 | serpin peptidase inhibitor, clade B (ovalbumin), member 7 [Source:HGNC Symbol;Acc:13902] |
| SERPINB8 | ENSG00000166401 | serpin peptidase inhibitor, clade B (ovalbumin), member 8 [Source:HGNC Symbol;Acc:8952] |
| RIC3 | ENSG00000166405 | RIC3 acetylcholine receptor chaperone [Source:HGNC Symbol;Acc:30338] |
| BEAN1 | ENSG00000166546 | brain expressed, associated with NEDD4, 1 [Source:HGNC Symbol;Acc:24160] |
| SERPINB12 | ENSG00000166634 | serpin peptidase inhibitor, clade B (ovalbumin), member 12 [Source:HGNC Symbol;Acc:14220] |
| PLEKHA7 | ENSG00000166689 | pleckstrin homology domain containing, family A member 7 [Source:HGNC Symbol;Acc:27049] |
| B2M | ENSG00000166710 | beta-2-microglobulin [Source:HGNC Symbol;Acc:914] |
| SLFN5 | ENSG00000166750 | schlafen family member 5 [Source:HGNC Symbol;Acc:28286] |
| PATL1 | ENSG00000166889 | protein associated with topoisomerase II homolog 1 (yeast) [Source:HGNC Symbol;Acc:26721] |
| C15orf48 | ENSG00000166920 | chromosome 15 open reading frame 48 [Source:HGNC Symbol;Acc:29898] |
| NKX3-1 | ENSG00000167034 | NK3 homeobox 1 [Source:HGNC Symbol;Acc:7838] |
| TMEM92 | ENSG00000167105 | transmembrane protein 92 [Source:HGNC Symbol;Acc:26579] |
| PRRX2 | ENSG00000167157 | paired related homeobox 2 [Source:HGNC Symbol;Acc:21338] |
| NOD2 | ENSG00000167207 | nucleotide-binding oligomerization domain containing 2 [Source:HGNC Symbol;Acc:5331] |
| IGF2 | ENSG00000167244 | insulin-like growth factor 2 (somatomedin A) [Source:HGNC Symbol;Acc:5466] |
| PRRT2 | ENSG00000167371 | proline-rich transmembrane protein 2 [Source:HGNC Symbol;Acc:30500] |
| MVD | ENSG00000167508 | mevalonate (diphospho) decarboxylase [Source:HGNC Symbol;Acc:7529] |
| GPD1 | ENSG00000167588 | glycerol-3-phosphate dehydrogenase 1 (soluble) [Source:HGNC Symbol;Acc:4455] |
| SLC43A2 | ENSG00000167703 | solute carrier family 43 (amino acid system L transporter), member 2 [Source:HGNC Symbol;Acc:23087] |
| KLK1 | ENSG00000167748 | kallikrein 1 [Source:HGNC Symbol;Acc:6357] |
| KLK11 | ENSG00000167757 | kallikrein-related peptidase 11 [Source:HGNC Symbol;Acc:6359] |
| KLK13 | ENSG00000167759 | kallikrein-related peptidase 13 [Source:HGNC Symbol;Acc:6361] |
| ANGPTL4 | ENSG00000167772 | angiopoietin-like 4 [Source:HGNC Symbol;Acc:16039] |
| IGFBP6 | ENSG00000167779 | insulin-like growth factor binding protein 6 [Source:HGNC Symbol;Acc:5475] |
| CD300A | ENSG00000167851 | CD300a molecule [Source:HGNC Symbol;Acc:19319] |
| KRT24 | ENSG00000167916 | keratin 24 [Source:HGNC Symbol;Acc:18527] |
| TRANK1 | ENSG00000168016 | tetratricopeptide repeat and ankyrin repeat containing 1 [Source:HGNC Symbol;Acc:29011] |
| BATF2 | ENSG00000168062 | basic leucine zipper transcription factor, ATF-like 2 [Source:HGNC Symbol;Acc:25163] |
| SCARA3 | ENSG00000168077 | scavenger receptor class A, member 3 [Source:HGNC Symbol;Acc:19000] |
| FOXI1 | ENSG00000168269 | forkhead box I1 [Source:HGNC Symbol;Acc:3815] |
| PXK | ENSG00000168297 | PX domain containing serine/threonine kinase [Source:HGNC Symbol;Acc:23326] |
| IRF2 | ENSG00000168310 | interferon regulatory factor 2 [Source:HGNC Symbol;Acc:6117] |
| TAP1 | ENSG00000168394 | transporter 1, ATP-binding cassette, sub-family B (MDR/TAP) [Source:HGNC Symbol;Acc:43] |
| MLKL | ENSG00000168404 | mixed lineage kinase domain-like [Source:HGNC Symbol;Acc:26617] |
| CMAHP | ENSG00000168405 | cytidine monophospho-N-acetylneuraminic acid hydroxylase, pseudogene [Source:HGNC Symbol;Acc:2098] |
| AXIN2 | ENSG00000168646 | axin 2 [Source:HGNC Symbol;Acc:904] |
| SLC16A4 | ENSG00000168679 | solute carrier family 16, member 4 [Source:HGNC Symbol;Acc:10925] |
| IL7R | ENSG00000168685 | interleukin 7 receptor [Source:HGNC Symbol;Acc:6024] |
| TET2 | ENSG00000168769 | tet methylcytosine dioxygenase 2 [Source:HGNC Symbol;Acc:25941] |
| IL12A | ENSG00000168811 | interleukin 12A (natural killer cell stimulatory factor 1, cytotoxic lymphocyte maturation factor 1, p35) [Source:HGNC Symbol;Acc:5969] |
| PLA2G4F | ENSG00000168907 | phospholipase A2, group IVF [Source:HGNC Symbol;Acc:27396] |
| STXBP6 | ENSG00000168952 | syntaxin binding protein 6 (amisyn) [Source:HGNC Symbol;Acc:19666] |
| LGALS9 | ENSG00000168961 | lectin, galactoside-binding, soluble, 9 [Source:HGNC Symbol;Acc:6570] |
| ACTBL2 | ENSG00000169067 | actin, beta-like 2 [Source:HGNC Symbol;Acc:17780] |
| ATF5 | ENSG00000169136 | activating transcription factor 5 [Source:HGNC Symbol;Acc:790] |
| RAB24 | ENSG00000169228 | RAB24, member RAS oncogene family [Source:HGNC Symbol;Acc:9765] |
| CXCL10 | ENSG00000169245 | chemokine (C-X-C motif) ligand 10 [Source:HGNC Symbol;Acc:10637] |
| SH3TC2 | ENSG00000169247 | SH3 domain and tetratricopeptide repeats 2 [Source:HGNC Symbol;Acc:29427] |
| CXCL11 | ENSG00000169248 | chemokine (C-X-C motif) ligand 11 [Source:HGNC Symbol;Acc:10638] |
| ADRB2 | ENSG00000169252 | adrenoceptor beta 2, surface [Source:HGNC Symbol;Acc:286] |
| B3GALNT1 | ENSG00000169255 | beta-1,3-N-acetylgalactosaminyltransferase 1 (globoside blood group) [Source:HGNC Symbol;Acc:918] |
| GPRIN1 | ENSG00000169258 | G protein regulated inducer of neurite outgrowth 1 [Source:HGNC Symbol;Acc:24835] |
| PTAFR | ENSG00000169403 | platelet-activating factor receptor [Source:HGNC Symbol;Acc:9582] |
| IL8 | ENSG00000169429 | interleukin 8 [Source:HGNC Symbol;Acc:6025] |
| SPRR1A | ENSG00000169474 | small proline-rich protein 1A [Source:HGNC Symbol;Acc:11259] |
| CLIC4 | ENSG00000169504 | chloride intracellular channel 4 [Source:HGNC Symbol;Acc:13518] |
| MUC15 | ENSG00000169550 | mucin 15, cell surface associated [Source:HGNC Symbol;Acc:14956] |
| CLIC3 | ENSG00000169583 | chloride intracellular channel 3 [Source:HGNC Symbol;Acc:2064] |
| HEXDC | ENSG00000169660 | hexosaminidase (glycosyl hydrolase family 20, catalytic domain) containing [Source:HGNC Symbol;Acc:26307] |
| FASN | ENSG00000169710 | fatty acid synthase [Source:HGNC Symbol;Acc:3594] |
| MT1E | ENSG00000169715 | metallothionein 1E [Source:HGNC Symbol;Acc:7397] |
| TRIM56 | ENSG00000169871 | tripartite motif containing 56 [Source:HGNC Symbol;Acc:19028] |
| ZFPM2 | ENSG00000169946 | zinc finger protein, FOG family member 2 [Source:HGNC Symbol;Acc:16700] |
| MYRIP | ENSG00000170011 | myosin VIIA and Rab interacting protein [Source:HGNC Symbol;Acc:19156] |
| ZPLD1 | ENSG00000170044 | zona pellucida-like domain containing 1 [Source:HGNC Symbol;Acc:27022] |
| LGALS9B | ENSG00000170298 | lectin, galactoside-binding, soluble, 9B [Source:HGNC Symbol;Acc:24842] |
| B3GNT2 | ENSG00000170340 | UDP-GlcNAc:betaGal beta-1,3-N-acetylglucosaminyltransferase 2 [Source:HGNC Symbol;Acc:15629] |
| OR2A20P | ENSG00000170356 | olfactory receptor, family 2, subfamily A, member 20 pseudogene [Source:HGNC Symbol;Acc:15413] |
| LRRN2 | ENSG00000170382 | leucine rich repeat neuronal 2 [Source:HGNC Symbol;Acc:16914] |
| ZNF804A | ENSG00000170396 | zinc finger protein 804A [Source:HGNC Symbol;Acc:21711] |
| GPRC5C | ENSG00000170412 | G protein-coupled receptor, family C, group 5, member C [Source:HGNC Symbol;Acc:13309] |
| KRT6C | ENSG00000170465 | keratin 6C [Source:HGNC Symbol;Acc:20406] |
| SERPINB9 | ENSG00000170542 | serpin peptidase inhibitor, clade B (ovalbumin), member 9 [Source:HGNC Symbol;Acc:8955] |
| STAT2 | ENSG00000170581 | signal transducer and activator of transcription 2, 113kDa [Source:HGNC Symbol;Acc:11363] |
| KCNS3 | ENSG00000170745 | potassium voltage-gated channel, delayed-rectifier, subfamily S, member 3 [Source:HGNC Symbol;Acc:6302] |
| GPR37 | ENSG00000170775 | G protein-coupled receptor 37 (endothelin receptor type B-like) [Source:HGNC Symbol;Acc:4494] |
| SDR16C5 | ENSG00000170786 | short chain dehydrogenase/reductase family 16C, member 5 [Source:HGNC Symbol;Acc:30311] |
| FOXN2 | ENSG00000170802 | forkhead box N2 [Source:HGNC Symbol;Acc:5281] |
| CEACAM3 | ENSG00000170956 | carcinoembryonic antigen-related cell adhesion molecule 3 [Source:HGNC Symbol;Acc:1815] |
| PRKCE | ENSG00000171132 | protein kinase C, epsilon [Source:HGNC Symbol;Acc:9401] |
| JUNB | ENSG00000171223 | jun B proto-oncogene [Source:HGNC Symbol;Acc:6205] |
| LRG1 | ENSG00000171236 | leucine-rich alpha-2-glycoprotein 1 [Source:HGNC Symbol;Acc:29480] |
| SOSTDC1 | ENSG00000171243 | sclerostin domain containing 1 [Source:HGNC Symbol;Acc:21748] |
| KCND3 | ENSG00000171385 | potassium voltage-gated channel, Shal-related subfamily, member 3 [Source:HGNC Symbol;Acc:6239] |
| COL24A1 | ENSG00000171502 | collagen, type XXIV, alpha 1 [Source:HGNC Symbol;Acc:20821] |
| DEFB4A | ENSG00000171711 | defensin, beta 4A [Source:HGNC Symbol;Acc:2767] |
| BCL2 | ENSG00000171791 | B-cell CLL/lymphoma 2 [Source:HGNC Symbol;Acc:990] |
| IFNB1 | ENSG00000171855 | interferon, beta 1, fibroblast [Source:HGNC Symbol;Acc:5434] |
| C3AR1 | ENSG00000171860 | complement component 3a receptor 1 [Source:HGNC Symbol;Acc:1319] |
| CYP4F11 | ENSG00000171903 | cytochrome P450, family 4, subfamily F, polypeptide 11 [Source:HGNC Symbol;Acc:13265] |
| LGALS9C | ENSG00000171916 | lectin, galactoside-binding, soluble, 9C [Source:HGNC Symbol;Acc:33874] |
| EPHX4 | ENSG00000172031 | epoxide hydrolase 4 [Source:HGNC Symbol;Acc:23758] |
| SLFN12 | ENSG00000172123 | schlafen family member 12 [Source:HGNC Symbol;Acc:25500] |
| FRMD3 | ENSG00000172159 | FERM domain containing 3 [Source:HGNC Symbol;Acc:24125] |
| ISG20 | ENSG00000172183 | interferon stimulated exonuclease gene 20kDa [Source:HGNC Symbol;Acc:6130] |
| ID4 | ENSG00000172201 | inhibitor of DNA binding 4, dominant negative helix-loop-helix protein [Source:HGNC Symbol;Acc:5363] |
| CLEC7A | ENSG00000172243 | C-type lectin domain family 7, member A [Source:HGNC Symbol;Acc:14558] |
| STARD5 | ENSG00000172345 | StAR-related lipid transfer (START) domain containing 5 [Source:HGNC Symbol;Acc:18065] |
| SYNPO2 | ENSG00000172403 | synaptopodin 2 [Source:HGNC Symbol;Acc:17732] |
| GTPBP2 | ENSG00000172432 | GTP binding protein 2 [Source:HGNC Symbol;Acc:4670] |
| RASGRP1 | ENSG00000172575 | RAS guanyl releasing protein 1 (calcium and DAG-regulated) [Source:HGNC Symbol;Acc:9878] |
| C17orf66 | ENSG00000172653 | chromosome 17 open reading frame 66 [Source:HGNC Symbol;Acc:26548] |
| TMEM217 | ENSG00000172738 | transmembrane protein 217 [Source:HGNC Symbol;Acc:21238] |
| OVOL1 | ENSG00000172818 | ovo-like zinc finger 1 [Source:HGNC Symbol;Acc:8525] |
| MYD88 | ENSG00000172936 | myeloid differentiation primary response 88 [Source:HGNC Symbol;Acc:7562] |
| UNC93B3 | ENSG00000172971 | unc-93 homolog B3 pseudogene (C. elegans) [Source:HGNC Symbol;Acc:13483] |
| HPSE | ENSG00000173083 | heparanase [Source:HGNC Symbol;Acc:5164] |
| HSPA6 | ENSG00000173110 | heat shock 70kDa protein 6 (HSP70B') [Source:HGNC Symbol;Acc:5239] |
| PARP14 | ENSG00000173193 | poly (ADP-ribose) polymerase family, member 14 [Source:HGNC Symbol;Acc:29232] |
| CYSLTR1 | ENSG00000173198 | cysteinyl leukotriene receptor 1 [Source:HGNC Symbol;Acc:17451] |
| PARP15 | ENSG00000173200 | poly (ADP-ribose) polymerase family, member 15 [Source:HGNC Symbol;Acc:26876] |
| ABCD2 | ENSG00000173208 | ATP-binding cassette, sub-family D (ALD), member 2 [Source:HGNC Symbol;Acc:66] |
| MAB21L3 | ENSG00000173212 | mab-21-like 3 (C. elegans) [Source:HGNC Symbol;Acc:26787] |
| GLRX | ENSG00000173221 | glutaredoxin (thioltransferase) [Source:HGNC Symbol;Acc:4330] |
| DMRT2 | ENSG00000173253 | doublesex and mab-3 related transcription factor 2 [Source:HGNC Symbol;Acc:2935] |
| MMRN2 | ENSG00000173269 | multimerin 2 [Source:HGNC Symbol;Acc:19888] |
| SAA1 | ENSG00000173432 | serum amyloid A1 [Source:HGNC Symbol;Acc:10513] |
| MUC13 | ENSG00000173702 | mucin 13, cell surface associated [Source:HGNC Symbol;Acc:7511] |
| CNP | ENSG00000173786 | 2',3'-cyclic nucleotide 3' phosphodiesterase [Source:HGNC Symbol;Acc:2158] |
| HAP1 | ENSG00000173805 | huntingtin-associated protein 1 [Source:HGNC Symbol;Acc:4812] |
| RNF213 | ENSG00000173821 | ring finger protein 213 [Source:HGNC Symbol;Acc:14539] |
| C1QTNF1 | ENSG00000173918 | C1q and tumor necrosis factor related protein 1 [Source:HGNC Symbol;Acc:14324] |
| SLCO4C1 | ENSG00000173930 | solute carrier organic anion transporter family, member 4C1 [Source:HGNC Symbol;Acc:23612] |
| CD34 | ENSG00000174059 | CD34 molecule [Source:HGNC Symbol;Acc:1662] |
| TLR1 | ENSG00000174125 | toll-like receptor 1 [Source:HGNC Symbol;Acc:11847] |
| KIAA1239 | ENSG00000174145 | KIAA1239 [Source:HGNC Symbol;Acc:29229] |
| ANKRD36C | ENSG00000174501 | ankyrin repeat domain 36C [Source:HGNC Symbol;Acc:32946] |
| UGT8 | ENSG00000174607 | UDP glycosyltransferase 8 [Source:HGNC Symbol;Acc:12555] |
| KIAA1551 | ENSG00000174718 | KIAA1551 [Source:HGNC Symbol;Acc:25559] |
| BTC | ENSG00000174808 | betacellulin [Source:HGNC Symbol;Acc:1121] |
| ZDHHC14 | ENSG00000175048 | zinc finger, DHHC-type containing 14 [Source:HGNC Symbol;Acc:20341] |
| MARCKSL1 | ENSG00000175130 | MARCKS-like 1 [Source:HGNC Symbol;Acc:7142] |
| CADM2 | ENSG00000175161 | cell adhesion molecule 2 [Source:HGNC Symbol;Acc:29849] |
| ABO | ENSG00000175164 | ABO blood group (transferase A, alpha 1-3-N-acetylgalactosaminyltransferase; transferase B, alpha 1-3-galactosyltransferase) [Source:HGNC Symbol;Acc:79] |
| DDIT3 | ENSG00000175197 | DNA-damage-inducible transcript 3 [Source:HGNC Symbol;Acc:2726] |
| CHRNA7 | ENSG00000175344 | cholinergic receptor, nicotinic, alpha 7 (neuronal) [Source:HGNC Symbol;Acc:1960] |
| UBQLNL | ENSG00000175518 | ubiquilin-like [Source:HGNC Symbol;Acc:28294] |
| LONRF3 | ENSG00000175556 | LON peptidase N-terminal domain and ring finger 3 [Source:HGNC Symbol;Acc:21152] |
| RMI2 | ENSG00000175643 | RecQ mediated genome instability 2 [Source:HGNC Symbol;Acc:28349] |
| BAK1P1 | ENSG00000175730 | BCL2-antagonist/killer 1 pseudogene 1 [Source:HGNC Symbol;Acc:996] |
| SFN | ENSG00000175793 | stratifin [Source:HGNC Symbol;Acc:10773] |
| ASCL3 | ENSG00000176009 | achaete-scute family bHLH transcription factor 3 [Source:HGNC Symbol;Acc:740] |
| NUPR1 | ENSG00000176046 | nuclear protein, transcriptional regulator, 1 [Source:HGNC Symbol;Acc:29990] |
| RIMS2 | ENSG00000176406 | regulating synaptic membrane exocytosis 2 [Source:HGNC Symbol;Acc:17283] |
| CCDC101 | ENSG00000176476 | coiled-coil domain containing 101 [Source:HGNC Symbol;Acc:25156] |
| PRR15 | ENSG00000176532 | proline rich 15 [Source:HGNC Symbol;Acc:22310] |
| TRIL | ENSG00000176734 | TLR4 interactor with leucine-rich repeats [Source:HGNC Symbol;Acc:22200] |
| C8orf4 | ENSG00000176907 | chromosome 8 open reading frame 4 [Source:HGNC Symbol;Acc:1357] |
| GCNT4 | ENSG00000176928 | glucosaminyl (N-acetyl) transferase 4, core 2 [Source:HGNC Symbol;Acc:17973] |
| MUC20 | ENSG00000176945 | mucin 20, cell surface associated [Source:HGNC Symbol;Acc:23282] |
| B3GNT8 | ENSG00000177191 | UDP-GlcNAc:betaGal beta-1,3-N-acetylglucosaminyltransferase 8 [Source:HGNC Symbol;Acc:24139] |
| DEFB4B | ENSG00000177257 | defensin, beta 4B [Source:HGNC Symbol;Acc:30193] |
| FBXO39 | ENSG00000177294 | F-box protein 39 [Source:HGNC Symbol;Acc:28565] |
| C8orf31 | ENSG00000177335 | chromosome 8 open reading frame 31 [Source:HGNC Symbol;Acc:26731] |
| PPFIA3 | ENSG00000177380 | protein tyrosine phosphatase, receptor type, f polypeptide (PTPRF), interacting protein (liprin), alpha 3 [Source:HGNC Symbol;Acc:9247] |
| SAMD9L | ENSG00000177409 | sterile alpha motif domain containing 9-like [Source:HGNC Symbol;Acc:1349] |
| ODF3B | ENSG00000177989 | outer dense fiber of sperm tails 3B [Source:HGNC Symbol;Acc:34388] |
| GPR150 | ENSG00000178015 | G protein-coupled receptor 150 [Source:HGNC Symbol;Acc:23628] |
| ADAMTSL1 | ENSG00000178031 | ADAMTS-like 1 [Source:HGNC Symbol;Acc:14632] |
| FAM26E | ENSG00000178033 | family with sequence similarity 26, member E [Source:HGNC Symbol;Acc:21568] |
| GRAMD1C | ENSG00000178075 | GRAM domain containing 1C [Source:HGNC Symbol;Acc:25252] |
| SH2D4B | ENSG00000178217 | SH2 domain containing 4B [Source:HGNC Symbol;Acc:31440] |
| PARP10 | ENSG00000178685 | poly (ADP-ribose) polymerase family, member 10 [Source:HGNC Symbol;Acc:25895] |
| STX19 | ENSG00000178750 | syntaxin 19 [Source:HGNC Symbol;Acc:19300] |
| TMEM139 | ENSG00000178826 | transmembrane protein 139 [Source:HGNC Symbol;Acc:22058] |
| EFCAB13 | ENSG00000178852 | EF-hand calcium binding domain 13 [Source:HGNC Symbol;Acc:26864] |
| FOXE1 | ENSG00000178919 | forkhead box E1 (thyroid transcription factor 2) [Source:HGNC Symbol;Acc:3806] |
| C17orf62 | ENSG00000178927 | chromosome 17 open reading frame 62 [Source:HGNC Symbol;Acc:28672] |
| C14orf39 | ENSG00000179008 | chromosome 14 open reading frame 39 [Source:HGNC Symbol;Acc:19849] |
| C3orf38 | ENSG00000179021 | chromosome 3 open reading frame 38 [Source:HGNC Symbol;Acc:28384] |
| KLHDC7A | ENSG00000179023 | kelch domain containing 7A [Source:HGNC Symbol;Acc:26791] |
| EXOC3L1 | ENSG00000179044 | exocyst complex component 3-like 1 [Source:HGNC Symbol;Acc:27540] |
| CIITA | ENSG00000179583 | class II, major histocompatibility complex, transactivator [Source:HGNC Symbol;Acc:7067] |
| ALOX15B | ENSG00000179593 | arachidonate 15-lipoxygenase, type B [Source:HGNC Symbol;Acc:434] |
| ZBTB42 | ENSG00000179627 | zinc finger and BTB domain containing 42 [Source:HGNC Symbol;Acc:32550] |
| ARL14 | ENSG00000179674 | ADP-ribosylation factor-like 14 [Source:HGNC Symbol;Acc:22974] |
| APOBEC3B | ENSG00000179750 | apolipoprotein B mRNA editing enzyme, catalytic polypeptide-like 3B [Source:HGNC Symbol;Acc:17352] |
| MRGPRX3 | ENSG00000179826 | MAS-related GPR, member X3 [Source:HGNC Symbol;Acc:17980] |
| AKAP5 | ENSG00000179841 | A kinase (PRKA) anchor protein 5 [Source:HGNC Symbol;Acc:375] |
| ABCA13 | ENSG00000179869 | ATP-binding cassette, sub-family A (ABC1), member 13 [Source:HGNC Symbol;Acc:14638] |
| SSC5D | ENSG00000179954 | scavenger receptor cysteine rich domain containing (5 domains) [Source:HGNC Symbol;Acc:26641] |
| LYNX1 | ENSG00000180155 | Ly6/neurotoxin 1 [Source:HGNC Symbol;Acc:29604] |
| HIST1H2AC | ENSG00000180573 | histone cluster 1, H2ac [Source:HGNC Symbol;Acc:4733] |
| SKIDA1 | ENSG00000180592 | SKI/DACH domain containing 1 [Source:HGNC Symbol;Acc:32697] |
| HIST1H2BC | ENSG00000180596 | histone cluster 1, H2bc [Source:HGNC Symbol;Acc:4757] |
| PCGF5 | ENSG00000180628 | polycomb group ring finger 5 [Source:HGNC Symbol;Acc:28264] |
| C12orf36 | ENSG00000180861 | chromosome 12 open reading frame 36 [Source:HGNC Symbol;Acc:26598] |
| OR52N4 | ENSG00000181074 | olfactory receptor, family 52, subfamily N, member 4 (gene/pseudogene) [Source:HGNC Symbol;Acc:15230] |
| MUC16 | ENSG00000181143 | mucin 16, cell surface associated [Source:HGNC Symbol;Acc:15582] |
| HEPHL1 | ENSG00000181333 | hephaestin-like 1 [Source:HGNC Symbol;Acc:30477] |
| DDX60L | ENSG00000181381 | DEAD (Asp-Glu-Ala-Asp) box polypeptide 60-like [Source:HGNC Symbol;Acc:26429] |
| ZNF467 | ENSG00000181444 | zinc finger protein 467 [Source:HGNC Symbol;Acc:23154] |
| SOX2 | ENSG00000181449 | SRY (sex determining region Y)-box 2 [Source:HGNC Symbol;Acc:11195] |
| C6orf223 | ENSG00000181577 | chromosome 6 open reading frame 223 [Source:HGNC Symbol;Acc:28692] |
| TNFSF15 | ENSG00000181634 | tumor necrosis factor (ligand) superfamily, member 15 [Source:HGNC Symbol;Acc:11931] |
| PHLDA2 | ENSG00000181649 | pleckstrin homology-like domain, family A, member 2 [Source:HGNC Symbol;Acc:12385] |
| SLC9A9 | ENSG00000181804 | solute carrier family 9, subfamily A (NHE9, cation proton antiporter 9), member 9 [Source:HGNC Symbol;Acc:20653] |
| RTKN2 | ENSG00000182010 | rhotekin 2 [Source:HGNC Symbol;Acc:19364] |
| USH1G | ENSG00000182040 | Usher syndrome 1G (autosomal recessive) [Source:HGNC Symbol;Acc:16356] |
| UBA7 | ENSG00000182179 | ubiquitin-like modifier activating enzyme 7 [Source:HGNC Symbol;Acc:12471] |
| EXT1 | ENSG00000182197 | exostosin glycosyltransferase 1 [Source:HGNC Symbol;Acc:3512] |
| HIST2H4B | ENSG00000182217 | histone cluster 2, H4b [Source:HGNC Symbol;Acc:29607] |
| C1S | ENSG00000182326 | complement component 1, s subcomponent [Source:HGNC Symbol;Acc:1247] |
| IFNL1 | ENSG00000182393 | interferon, lambda 1 [Source:HGNC Symbol;Acc:18363] |
| NCF1B | ENSG00000182487 | neutrophil cytosolic factor 1B pseudogene [Source:HGNC Symbol;Acc:32522] |
| LHFPL1 | ENSG00000182508 | lipoma HMGIC fusion partner-like 1 [Source:HGNC Symbol;Acc:6587] |
| EPGN | ENSG00000182585 | epithelial mitogen [Source:HGNC Symbol;Acc:17470] |
| HCAR2 | ENSG00000182782 | hydroxycarboxylic acid receptor 2 [Source:HGNC Symbol;Acc:24827] |
| C1orf116 | ENSG00000182795 | chromosome 1 open reading frame 116 [Source:HGNC Symbol;Acc:28667] |
| VMO1 | ENSG00000182853 | vitelline membrane outer layer 1 homolog (chicken) [Source:HGNC Symbol;Acc:30387] |
| COL18A1 | ENSG00000182871 | collagen, type XVIII, alpha 1 [Source:HGNC Symbol;Acc:2195] |
| LENG9 | ENSG00000182909 | leukocyte receptor cluster (LRC) member 9 [Source:HGNC Symbol;Acc:16306] |
| SLC8A1 | ENSG00000183023 | solute carrier family 8 (sodium/calcium exchanger), member 1 [Source:HGNC Symbol;Acc:11068] |
| ABAT | ENSG00000183044 | 4-aminobutyrate aminotransferase [Source:HGNC Symbol;Acc:23] |
| ABHD16B | ENSG00000183260 | abhydrolase domain containing 16B [Source:HGNC Symbol;Acc:16128] |
| GBP6 | ENSG00000183347 | guanylate binding protein family, member 6 [Source:HGNC Symbol;Acc:25395] |
| FHL3 | ENSG00000183386 | four and a half LIM domains 3 [Source:HGNC Symbol;Acc:3704] |
| MX2 | ENSG00000183486 | myxovirus (influenza virus) resistance 2 (mouse) [Source:HGNC Symbol;Acc:7533] |
| FAM46C | ENSG00000183508 | family with sequence similarity 46, member C [Source:HGNC Symbol;Acc:24712] |
| HIST2H2AA3 | ENSG00000183558 | histone cluster 2, H2aa3 [Source:HGNC Symbol;Acc:4736] |
| CCR3 | ENSG00000183625 | chemokine (C-C motif) receptor 3 [Source:HGNC Symbol;Acc:1604] |
| CXorf64 | ENSG00000183631 | chromosome X open reading frame 64 [Source:HGNC Symbol;Acc:34498] |
| IFNL2 | ENSG00000183709 | interferon, lambda 2 [Source:HGNC Symbol;Acc:18364] |
| LHFP | ENSG00000183722 | lipoma HMGIC fusion partner [Source:HGNC Symbol;Acc:6586] |
| TMEM50A | ENSG00000183726 | transmembrane protein 50A [Source:HGNC Symbol;Acc:30590] |
| ASCL2 | ENSG00000183734 | achaete-scute family bHLH transcription factor 2 [Source:HGNC Symbol;Acc:739] |
| ARSI | ENSG00000183876 | arylsulfatase family, member I [Source:HGNC Symbol;Acc:32521] |
| HIST2H4A | ENSG00000183941 | histone cluster 2, H4a [Source:HGNC Symbol;Acc:4794] |
| TBX1 | ENSG00000184058 | T-box 1 [Source:HGNC Symbol;Acc:11592] |
| PCDH9 | ENSG00000184226 | protocadherin 9 [Source:HGNC Symbol;Acc:8661] |
| ACOT1 | ENSG00000184227 | acyl-CoA thioesterase 1 [Source:HGNC Symbol;Acc:33128] |
| OAF | ENSG00000184232 | OAF homolog (Drosophila) [Source:HGNC Symbol;Acc:28752] |
| HIST2H2AC | ENSG00000184260 | histone cluster 2, H2ac [Source:HGNC Symbol;Acc:4738] |
| PKP3 | ENSG00000184363 | plakophilin 3 [Source:HGNC Symbol;Acc:9025] |
| CSF1 | ENSG00000184371 | colony stimulating factor 1 (macrophage) [Source:HGNC Symbol;Acc:2432] |
| COLEC10 | ENSG00000184374 | collectin sub-family member 10 (C-type lectin) [Source:HGNC Symbol;Acc:2220] |
| PTP4A3 | ENSG00000184489 | protein tyrosine phosphatase type IVA, member 3 [Source:HGNC Symbol;Acc:9636] |
| DUSP8 | ENSG00000184545 | dual specificity phosphatase 8 [Source:HGNC Symbol;Acc:3074] |
| SOCS3 | ENSG00000184557 | suppressor of cytokine signaling 3 [Source:HGNC Symbol;Acc:19391] |
| SLITRK6 | ENSG00000184564 | SLIT and NTRK-like family, member 6 [Source:HGNC Symbol;Acc:23503] |
| LPAR5 | ENSG00000184574 | lysophosphatidic acid receptor 5 [Source:HGNC Symbol;Acc:13307] |
| TMEM173 | ENSG00000184584 | transmembrane protein 173 [Source:HGNC Symbol;Acc:27962] |
| HIST2H2BE | ENSG00000184678 | histone cluster 2, H2be [Source:HGNC Symbol;Acc:4760] |
| APOBR | ENSG00000184730 | apolipoprotein B receptor [Source:HGNC Symbol;Acc:24087] |
| UNC93B5 | ENSG00000184795 | unc-93 homolog B5 pseudogene (C. elegans) [Source:HGNC Symbol;Acc:34051] |
| ZBTB7C | ENSG00000184828 | zinc finger and BTB domain containing 7C [Source:HGNC Symbol;Acc:31700] |
| RBM43 | ENSG00000184898 | RNA binding motif protein 43 [Source:HGNC Symbol;Acc:24790] |
| USP18 | ENSG00000184979 | ubiquitin specific peptidase 18 [Source:HGNC Symbol;Acc:12616] |
| TMEM106A | ENSG00000184988 | transmembrane protein 106A [Source:HGNC Symbol;Acc:28288] |
| IFNE | ENSG00000184995 | interferon, epsilon [Source:HGNC Symbol;Acc:18163] |
| MAFF | ENSG00000185022 | v-maf avian musculoaponeurotic fibrosarcoma oncogene homolog F [Source:HGNC Symbol;Acc:6780] |
| KRT76 | ENSG00000185069 | keratin 76 [Source:HGNC Symbol;Acc:24430] |
| FAM169B | ENSG00000185087 | family with sequence similarity 169, member B [Source:HGNC Symbol;Acc:26835] |
| FAM43A | ENSG00000185112 | family with sequence similarity 43, member A [Source:HGNC Symbol;Acc:26888] |
| NRBP2 | ENSG00000185189 | nuclear receptor binding protein 2 [Source:HGNC Symbol;Acc:19339] |
| IFITM2 | ENSG00000185201 | interferon induced transmembrane protein 2 [Source:HGNC Symbol;Acc:5413] |
| TNFAIP2 | ENSG00000185215 | tumor necrosis factor, alpha-induced protein 2 [Source:HGNC Symbol;Acc:11895] |
| UBALD2 | ENSG00000185262 | UBA-like domain containing 2 [Source:HGNC Symbol;Acc:28438] |
| RBM11 | ENSG00000185272 | RNA binding motif protein 11 [Source:HGNC Symbol;Acc:9897] |
| SOCS1 | ENSG00000185338 | suppressor of cytokine signaling 1 [Source:HGNC Symbol;Acc:19383] |
| TCN2 | ENSG00000185339 | transcobalamin II [Source:HGNC Symbol;Acc:11653] |
| MAPK11 | ENSG00000185386 | mitogen-activated protein kinase 11 [Source:HGNC Symbol;Acc:6873] |
| SP140L | ENSG00000185404 | SP140 nuclear body protein-like [Source:HGNC Symbol;Acc:25105] |
| KRT6B | ENSG00000185479 | keratin 6B [Source:HGNC Symbol;Acc:6444] |
| MUC1 | ENSG00000185499 | mucin 1, cell surface associated [Source:HGNC Symbol;Acc:7508] |
| IRF7 | ENSG00000185507 | interferon regulatory factor 7 [Source:HGNC Symbol;Acc:6122] |
| POU3F1 | ENSG00000185668 | POU class 3 homeobox 1 [Source:HGNC Symbol;Acc:9214] |
| ANKFY1 | ENSG00000185722 | ankyrin repeat and FYVE domain containing 1 [Source:HGNC Symbol;Acc:20763] |
| IFIT1 | ENSG00000185745 | interferon-induced protein with tetratricopeptide repeats 1 [Source:HGNC Symbol;Acc:5407] |
| IKZF1 | ENSG00000185811 | IKAROS family zinc finger 1 (Ikaros) [Source:HGNC Symbol;Acc:13176] |
| TRIM69 | ENSG00000185880 | tripartite motif containing 69 [Source:HGNC Symbol;Acc:17857] |
| IFITM1 | ENSG00000185885 | interferon induced transmembrane protein 1 [Source:HGNC Symbol;Acc:5412] |
| BPIFB4 | ENSG00000186191 | BPI fold containing family B, member 4 [Source:HGNC Symbol;Acc:16179] |
| EDARADD | ENSG00000186197 | EDAR-associated death domain [Source:HGNC Symbol;Acc:14341] |
| CYP4F12 | ENSG00000186204 | cytochrome P450, family 4, subfamily F, polypeptide 12 [Source:HGNC Symbol;Acc:18857] |
| GLDN | ENSG00000186417 | gliomedin [Source:HGNC Symbol;Acc:29514] |
| BTN3A2 | ENSG00000186470 | butyrophilin, subfamily 3, member A2 [Source:HGNC Symbol;Acc:1139] |
| PCLO | ENSG00000186472 | piccolo presynaptic cytomatrix protein [Source:HGNC Symbol;Acc:13406] |
| INSIG1 | ENSG00000186480 | insulin induced gene 1 [Source:HGNC Symbol;Acc:6083] |
| ANKRD20A5P | ENSG00000186481 | ankyrin repeat domain 20 family, member A5, pseudogene [Source:HGNC Symbol;Acc:33833] |
| LRRC16B | ENSG00000186648 | leucine rich repeat containing 16B [Source:HGNC Symbol;Acc:20272] |
| VSIG10L | ENSG00000186806 | V-set and immunoglobulin domain containing 10 like [Source:HGNC Symbol;Acc:27111] |
| ANXA8L2 | ENSG00000186807 | annexin A8-like 2 [Source:HGNC Symbol;Acc:23335] |
| KRT14 | ENSG00000186847 | keratin 14 [Source:HGNC Symbol;Acc:6416] |
| TRABD2A | ENSG00000186854 | TraB domain containing 2A [Source:HGNC Symbol;Acc:27013] |
| TMPRSS11A | ENSG00000187054 | transmembrane protease, serine 11A [Source:HGNC Symbol;Acc:27954] |
| KRT12 | ENSG00000187242 | keratin 12 [Source:HGNC Symbol;Acc:6414] |
| BCAM | ENSG00000187244 | basal cell adhesion molecule (Lutheran blood group) [Source:HGNC Symbol;Acc:6722] |
| COL4A1 | ENSG00000187498 | collagen, type IV, alpha 1 [Source:HGNC Symbol;Acc:2202] |
| PLEKHG7 | ENSG00000187510 | pleckstrin homology domain containing, family G (with RhoGef domain) member 7 [Source:HGNC Symbol;Acc:33829] |
| PLEKHN1 | ENSG00000187583 | pleckstrin homology domain containing, family N member 1 [Source:HGNC Symbol;Acc:25284] |
| ISG15 | ENSG00000187608 | ISG15 ubiquitin-like modifier [Source:HGNC Symbol;Acc:4053] |
| C1orf170 | ENSG00000187642 | chromosome 1 open reading frame 170 [Source:HGNC Symbol;Acc:28208] |
| SPRY4 | ENSG00000187678 | sprouty homolog 4 (Drosophila) [Source:HGNC Symbol;Acc:15533] |
| FANCA | ENSG00000187741 | Fanconi anemia, complementation group A [Source:HGNC Symbol;Acc:3582] |
| SEMA4D | ENSG00000187764 | sema domain, immunoglobulin domain (Ig), transmembrane domain (TM) and short cytoplasmic domain, (semaphorin) 4D [Source:HGNC Symbol;Acc:10732] |
| TMEM220 | ENSG00000187824 | transmembrane protein 220 [Source:HGNC Symbol;Acc:33757] |
| DMBT1 | ENSG00000187908 | deleted in malignant brain tumors 1 [Source:HGNC Symbol;Acc:2926] |
| C2orf66 | ENSG00000187944 | chromosome 2 open reading frame 66 [Source:HGNC Symbol;Acc:33809] |
| ARHGAP11B | ENSG00000187951 | Rho GTPase activating protein 11B [Source:HGNC Symbol;Acc:15782] |
| PLA2G4E | ENSG00000188089 | phospholipase A2, group IVE [Source:HGNC Symbol;Acc:24791] |
| AGRN | ENSG00000188157 | agrin [Source:HGNC Symbol;Acc:329] |
| IL17REL | ENSG00000188263 | interleukin 17 receptor E-like [Source:HGNC Symbol;Acc:33808] |
| RUFY4 | ENSG00000188282 | RUN and FYVE domain containing 4 [Source:HGNC Symbol;Acc:24804] |
| HES4 | ENSG00000188290 | hes family bHLH transcription factor 4 [Source:HGNC Symbol;Acc:24149] |
| PLSCR1 | ENSG00000188313 | phospholipid scramblase 1 [Source:HGNC Symbol;Acc:9092] |
| SBK1 | ENSG00000188322 | SH3 domain binding kinase 1 [Source:HGNC Symbol;Acc:17699] |
| SELL | ENSG00000188404 | selectin L [Source:HGNC Symbol;Acc:10720] |
| NDOR1 | ENSG00000188566 | NADPH dependent diflavin oxidoreductase 1 [Source:HGNC Symbol;Acc:29838] |
| ASAH2 | ENSG00000188611 | N-acylsphingosine amidohydrolase (non-lysosomal ceramidase) 2 [Source:HGNC Symbol;Acc:18860] |
| BCL2L15 | ENSG00000188761 | BCL2-like 15 [Source:HGNC Symbol;Acc:33624] |
| APOD | ENSG00000189058 | apolipoprotein D [Source:HGNC Symbol;Acc:612] |
| H1F0 | ENSG00000189060 | H1 histone family, member 0 [Source:HGNC Symbol;Acc:4714] |
| SP6 | ENSG00000189120 | Sp6 transcription factor [Source:HGNC Symbol;Acc:14530] |
| CXCL17 | ENSG00000189377 | chemokine (C-X-C motif) ligand 17 [Source:HGNC Symbol;Acc:19232] |
| GJB4 | ENSG00000189433 | gap junction protein, beta 4, 30.3kDa [Source:HGNC Symbol;Acc:4286] |
| SYCP2 | ENSG00000196074 | synaptonemal complex protein 2 [Source:HGNC Symbol;Acc:11490] |
| SPOCK3 | ENSG00000196104 | sparc/osteonectin, cwcv and kazal-like domains proteoglycan (testican) 3 [Source:HGNC Symbol;Acc:13565] |
| TDRD7 | ENSG00000196116 | tudor domain containing 7 [Source:HGNC Symbol;Acc:30831] |
| SPATS2L | ENSG00000196141 | spermatogenesis associated, serine-rich 2-like [Source:HGNC Symbol;Acc:24574] |
| SIRPB2 | ENSG00000196209 | signal-regulatory protein beta 2 [Source:HGNC Symbol;Acc:16247] |
| ZNF107 | ENSG00000196247 | zinc finger protein 107 [Source:HGNC Symbol;Acc:12887] |
| HIST1H2BO | ENSG00000196331 | histone cluster 1, H2bo [Source:HGNC Symbol;Acc:4758] |
| CD55 | ENSG00000196352 | CD55 molecule, decay accelerating factor for complement (Cromer blood group) [Source:HGNC Symbol;Acc:2665] |
| CPNE4 | ENSG00000196353 | copine IV [Source:HGNC Symbol;Acc:2317] |
| ARL9 | ENSG00000196503 | ADP-ribosylation factor-like 9 [Source:HGNC Symbol;Acc:23592] |
| SLC6A9 | ENSG00000196517 | solute carrier family 6 (neurotransmitter transporter, glycine), member 9 [Source:HGNC Symbol;Acc:11056] |
| SPTSSB | ENSG00000196542 | serine palmitoyltransferase, small subunit B [Source:HGNC Symbol;Acc:24045] |
| TCF4 | ENSG00000196628 | transcription factor 4 [Source:HGNC Symbol;Acc:11634] |
| HSH2D | ENSG00000196684 | hematopoietic SH2 domain containing [Source:HGNC Symbol;Acc:24920] |
| CD47 | ENSG00000196776 | CD47 molecule [Source:HGNC Symbol;Acc:1682] |
| HIST1H2AG | ENSG00000196787 | histone cluster 1, H2ag [Source:HGNC Symbol;Acc:4737] |
| MVB12B | ENSG00000196814 | multivesicular body subunit 12B [Source:HGNC Symbol;Acc:23368] |
| ARID5A | ENSG00000196843 | AT rich interactive domain 5A (MRF1-like) [Source:HGNC Symbol;Acc:17361] |
| KRT39 | ENSG00000196859 | keratin 39 [Source:HGNC Symbol;Acc:32971] |
| ANKRD36B | ENSG00000196912 | ankyrin repeat domain 36B [Source:HGNC Symbol;Acc:29333] |
| CASP4 | ENSG00000196954 | caspase 4, apoptosis-related cysteine peptidase [Source:HGNC Symbol;Acc:1505] |
| PCDHB16 | ENSG00000196963 | protocadherin beta 16 [Source:HGNC Symbol;Acc:14546] |
| GAL3ST4 | ENSG00000197093 | galactose-3-O-sulfotransferase 4 [Source:HGNC Symbol;Acc:24145] |
| IFNL3 | ENSG00000197110 | interferon, lambda 3 [Source:HGNC Symbol;Acc:18365] |
| PGAP1 | ENSG00000197121 | post-GPI attachment to proteins 1 [Source:HGNC Symbol;Acc:25712] |
| ACSL5 | ENSG00000197142 | acyl-CoA synthetase long-chain family member 5 [Source:HGNC Symbol;Acc:16526] |
| RAD54B | ENSG00000197275 | RAD54 homolog B (S. cerevisiae) [Source:HGNC Symbol;Acc:17228] |
| LYPD2 | ENSG00000197353 | LY6/PLAUR domain containing 2 [Source:HGNC Symbol;Acc:25215] |
| HIST1H3D | ENSG00000197409 | histone cluster 1, H3d [Source:HGNC Symbol;Acc:4767] |
| DCHS2 | ENSG00000197410 | dachsous cadherin-related 2 [Source:HGNC Symbol;Acc:23111] |
| STMN3 | ENSG00000197457 | stathmin-like 3 [Source:HGNC Symbol;Acc:15926] |
| PCDHB11 | ENSG00000197479 | protocadherin beta 11 [Source:HGNC Symbol;Acc:8682] |
| SLC28A3 | ENSG00000197506 | solute carrier family 28 (concentrative nucleoside transporter), member 3 [Source:HGNC Symbol;Acc:16484] |
| C5orf56 | ENSG00000197536 | chromosome 5 open reading frame 56 [Source:HGNC Symbol;Acc:33838] |
| ZNF841 | ENSG00000197608 | zinc finger protein 841 [Source:HGNC Symbol;Acc:27611] |
| DPP4 | ENSG00000197635 | dipeptidyl-peptidase 4 [Source:HGNC Symbol;Acc:3009] |
| SERPINB13 | ENSG00000197641 | serpin peptidase inhibitor, clade B (ovalbumin), member 13 [Source:HGNC Symbol;Acc:8944] |
| PDCD1LG2 | ENSG00000197646 | programmed cell death 1 ligand 2 [Source:HGNC Symbol;Acc:18731] |
| CR1L | ENSG00000197721 | complement component (3b/4b) receptor 1-like [Source:HGNC Symbol;Acc:2335] |
| CFD | ENSG00000197766 | complement factor D (adipsin) [Source:HGNC Symbol;Acc:2771] |
| HIST1H2BK | ENSG00000197903 | histone cluster 1, H2bk [Source:HGNC Symbol;Acc:13954] |
| C2orf27A | ENSG00000197927 | chromosome 2 open reading frame 27A [Source:HGNC Symbol;Acc:25077] |
| ZNF311 | ENSG00000197935 | zinc finger protein 311 [Source:HGNC Symbol;Acc:13847] |
| AKR1B10 | ENSG00000198074 | aldo-keto reductase family 1, member B10 (aldose reductase) [Source:HGNC Symbol;Acc:382] |
| TMEM229B | ENSG00000198133 | transmembrane protein 229B [Source:HGNC Symbol;Acc:20130] |
| ZNF334 | ENSG00000198185 | zinc finger protein 334 [Source:HGNC Symbol;Acc:15806] |
| CARD11 | ENSG00000198286 | caspase recruitment domain family, member 11 [Source:HGNC Symbol;Acc:16393] |
| PIM3 | ENSG00000198355 | pim-3 oncogene [Source:HGNC Symbol;Acc:19310] |
| B3GNT6 | ENSG00000198488 | UDP-GlcNAc:betaGal beta-1,3-N-acetylglucosaminyltransferase 6 (core 3 synthase) [Source:HGNC Symbol;Acc:24141] |
| C2CD4A | ENSG00000198535 | C2 calcium-dependent domain containing 4A [Source:HGNC Symbol;Acc:33627] |
| ITGBL1 | ENSG00000198542 | integrin, beta-like 1 (with EGF-like repeat domains) [Source:HGNC Symbol;Acc:6164] |
| BAZ1A | ENSG00000198604 | bromodomain adjacent to zinc finger domain, 1A [Source:HGNC Symbol;Acc:960] |
| TAT | ENSG00000198650 | tyrosine aminotransferase [Source:HGNC Symbol;Acc:11573] |
| EPS8L3 | ENSG00000198758 | EPS8-like 3 [Source:HGNC Symbol;Acc:21297] |
| GRIN3A | ENSG00000198785 | glutamate receptor, ionotropic, N-methyl-D-aspartate 3A [Source:HGNC Symbol;Acc:16767] |
| GRK5 | ENSG00000198873 | G protein-coupled receptor kinase 5 [Source:HGNC Symbol;Acc:4544] |
| SFMBT2 | ENSG00000198879 | Scm-like with four mbt domains 2 [Source:HGNC Symbol;Acc:20256] |
| TGM2 | ENSG00000198959 | transglutaminase 2 [Source:HGNC Symbol;Acc:11778] |
| RORB | ENSG00000198963 | RAR-related orphan receptor B [Source:HGNC Symbol;Acc:10259] |
| C1orf132 | ENSG00000203709 | chromosome 1 open reading frame 132 [Source:HGNC Symbol;Acc:32018] |
| CR1 | ENSG00000203710 | complement component (3b/4b) receptor 1 (Knops blood group) [Source:HGNC Symbol;Acc:2334] |
| SPRR2E | ENSG00000203785 | small proline-rich protein 2E [Source:HGNC Symbol;Acc:11265] |
| HIST2H3C | ENSG00000203811 | histone cluster 2, H3c [Source:HGNC Symbol;Acc:20503] |
| HIST2H2AA4 | ENSG00000203812 | histone cluster 2, H2aa4 [Source:HGNC Symbol;Acc:29668] |
| HIST2H2BF | ENSG00000203814 | histone cluster 2, H2bf [Source:HGNC Symbol;Acc:24700] |
| HIST2H2BC | ENSG00000203819 | histone cluster 2, H2bc (pseudogene) [Source:HGNC Symbol;Acc:20516] |
| HIST2H3A | ENSG00000203852 | histone cluster 2, H3a [Source:HGNC Symbol;Acc:20505] |
| IFIT1B | ENSG00000204010 | interferon-induced protein with tetratricopeptide repeats 1B [Source:HGNC Symbol;Acc:23442] |
| MAFB | ENSG00000204103 | v-maf avian musculoaponeurotic fibrosarcoma oncogene homolog B [Source:HGNC Symbol;Acc:6408] |
| PHACTR4 | ENSG00000204138 | phosphatase and actin regulator 4 [Source:HGNC Symbol;Acc:25793] |
| ASAH2B | ENSG00000204147 | N-acylsphingosine amidohydrolase (non-lysosomal ceramidase) 2B [Source:HGNC Symbol;Acc:23456] |
| GPRIN2 | ENSG00000204175 | G protein regulated inducer of neurite outgrowth 2 [Source:HGNC Symbol;Acc:23730] |
| SYT15 | ENSG00000204176 | synaptotagmin XV [Source:HGNC Symbol;Acc:17167] |
| BMPR2 | ENSG00000204217 | bone morphogenetic protein receptor, type II (serine/threonine kinase) [Source:HGNC Symbol;Acc:1078] |
| TAPSAR1 | ENSG00000204261 | TAP1 and PSMB8 antisense RNA 1 [Source:HGNC Symbol;Acc:39758] |
| PSMB8 | ENSG00000204264 | proteasome (prosome, macropain) subunit, beta type, 8 [Source:HGNC Symbol;Acc:9545] |
| TAP2 | ENSG00000204267 | transporter 2, ATP-binding cassette, sub-family B (MDR/TAP) [Source:HGNC Symbol;Acc:44] |
| HLA-DRA | ENSG00000204287 | major histocompatibility complex, class II, DR alpha [Source:HGNC Symbol;Acc:4947] |
| SMIM5 | ENSG00000204323 | small integral membrane protein 5 [Source:HGNC Symbol;Acc:40030] |
| CARD16 | ENSG00000204397 | caspase recruitment domain family, member 16 [Source:HGNC Symbol;Acc:33701] |
| MICB | ENSG00000204516 | MHC class I polypeptide-related sequence B [Source:HGNC Symbol;Acc:7091] |
| HLA-C | ENSG00000204525 | major histocompatibility complex, class I, C [Source:HGNC Symbol;Acc:4933] |
| PSORS1C3 | ENSG00000204528 | psoriasis susceptibility 1 candidate 3 (non-protein coding) [Source:HGNC Symbol;Acc:17203] |
| PSORS1C1 | ENSG00000204540 | psoriasis susceptibility 1 candidate 1 [Source:HGNC Symbol;Acc:17202] |
| MUC21 | ENSG00000204544 | mucin 21, cell surface associated [Source:HGNC Symbol;Acc:21661] |
| HLA-E | ENSG00000204592 | major histocompatibility complex, class I, E [Source:HGNC Symbol;Acc:4962] |
| TRIM31 | ENSG00000204616 | tripartite motif containing 31 [Source:HGNC Symbol;Acc:16289] |
| HLA-J | ENSG00000204622 | major histocompatibility complex, class I, J (pseudogene) [Source:HGNC Symbol;Acc:4967] |
| HLA-G | ENSG00000204632 | major histocompatibility complex, class I, G [Source:HGNC Symbol;Acc:4964] |
| HLA-F | ENSG00000204642 | major histocompatibility complex, class I, F [Source:HGNC Symbol;Acc:4963] |
| GABBR1 | ENSG00000204681 | gamma-aminobutyric acid (GABA) B receptor, 1 [Source:HGNC Symbol;Acc:4070] |
| CD177P1 | ENSG00000204933 | CD177 molecule pseudogene 1 [Source:HGNC Symbol;Acc:34508] |
| CD177 | ENSG00000204936 | CD177 molecule [Source:HGNC Symbol;Acc:30072] |
| PCDHA4 | ENSG00000204967 | protocadherin alpha 4 [Source:HGNC Symbol;Acc:8670] |
| CLLU1OS | ENSG00000205057 | chronic lymphocytic leukemia up-regulated 1 opposite strand [Source:HGNC Symbol;Acc:24070] |
| CCNI2 | ENSG00000205089 | cyclin I family, member 2 [Source:HGNC Symbol;Acc:33869] |
| PSMB10 | ENSG00000205220 | proteasome (prosome, macropain) subunit, beta type, 10 [Source:HGNC Symbol;Acc:9538] |
| MT1M | ENSG00000205364 | metallothionein 1M [Source:HGNC Symbol;Acc:14296] |
| CFI | ENSG00000205403 | complement factor I [Source:HGNC Symbol;Acc:5394] |
| SAMD9 | ENSG00000205413 | sterile alpha motif domain containing 9 [Source:HGNC Symbol;Acc:1348] |
| KRT6A | ENSG00000205420 | keratin 6A [Source:HGNC Symbol;Acc:6443] |
| GMNC | ENSG00000205835 | geminin coiled-coil domain containing [Source:HGNC Symbol;Acc:40049] |
| ONECUT3 | ENSG00000205922 | one cut homeobox 3 [Source:HGNC Symbol;Acc:13399] |
| SERPINB11 | ENSG00000206072 | serpin peptidase inhibitor, clade B (ovalbumin), member 11 (gene/pseudogene) [Source:HGNC Symbol;Acc:14221] |
| SERPINB4 | ENSG00000206073 | serpin peptidase inhibitor, clade B (ovalbumin), member 4 [Source:HGNC Symbol;Acc:10570] |
| ATP10A | ENSG00000206190 | ATPase, class V, type 10A [Source:HGNC Symbol;Acc:13542] |
| HCP5 | ENSG00000206337 | HLA complex P5 (non-protein coding) [Source:HGNC Symbol;Acc:21659] |
| HLA-H | ENSG00000206341 | major histocompatibility complex, class I, H (pseudogene) [Source:HGNC Symbol;Acc:4965] |
| COL6A6 | ENSG00000206384 | collagen, type VI, alpha 6 [Source:HGNC Symbol;Acc:27023] |
| HLA-A | ENSG00000206503 | major histocompatibility complex, class I, A [Source:HGNC Symbol;Acc:4931] |
| CD200R1L | ENSG00000206531 | CD200 receptor 1-like [Source:HGNC Symbol;Acc:24665] |
| GIMAP1 | ENSG00000213203 | GTPase, IMAP family member 1 [Source:HGNC Symbol;Acc:23237] |
| ARHGEF35 | ENSG00000213214 | Rho guanine nucleotide exchange factor (GEF) 35 [Source:HGNC Symbol;Acc:33846] |
| RBMS2P1 | ENSG00000213250 | RNA binding motif, single stranded interacting protein 2 pseudogene 1 [Source:HGNC Symbol;Acc:30994] |
| IFITM9P | ENSG00000213275 | interferon induced transmembrane protein 9 pseudogene [Source:HGNC Symbol;Acc:33972] |
| PTPRCAP | ENSG00000213402 | protein tyrosine phosphatase, receptor type, C-associated protein [Source:HGNC Symbol;Acc:9667] |
| NT5C3AP1 | ENSG00000213492 | 5'-nucleotidase, cytosolic IIIA pseudogene 1 [Source:HGNC Symbol;Acc:18530] |
| LAP3P2 | ENSG00000213500 | leucine aminopeptidase 3 pseudogene 2 [Source:HGNC Symbol;Acc:42365] |
| GBP7 | ENSG00000213512 | guanylate binding protein 7 [Source:HGNC Symbol;Acc:29606] |
| TMEM110 | ENSG00000213533 | transmembrane protein 110 [Source:HGNC Symbol;Acc:30526] |
| TREX1 | ENSG00000213689 | three prime repair exonuclease 1 [Source:HGNC Symbol;Acc:12269] |
| S1PR3 | ENSG00000213694 | sphingosine-1-phosphate receptor 3 [Source:HGNC Symbol;Acc:3167] |
| ACTBP2 | ENSG00000213763 | actin, beta pseudogene 2 [Source:HGNC Symbol;Acc:135] |
| IRF9 | ENSG00000213928 | interferon regulatory factor 9 [Source:HGNC Symbol;Acc:6131] |
| PAXIP1-AS2 | ENSG00000214106 | PAXIP1 antisense RNA 2 [Source:HGNC Symbol;Acc:48958] |
| CTAGE14P | ENSG00000214211 | CTAGE family, member 14, pseudogene [Source:HGNC Symbol;Acc:37299] |
| C19orf38 | ENSG00000214212 | chromosome 19 open reading frame 38 [Source:HGNC Symbol;Acc:34073] |
| C12orf74 | ENSG00000214215 | chromosome 12 open reading frame 74 [Source:HGNC Symbol;Acc:27887] |
| C17orf67 | ENSG00000214226 | chromosome 17 open reading frame 67 [Source:HGNC Symbol;Acc:27900] |
| LGMNP1 | ENSG00000214269 | legumain pseudogene 1 [Source:HGNC Symbol;Acc:23079] |
| ALDOAP2 | ENSG00000214297 | aldolase A, fructose-bisphosphate pseudogene 2 [Source:HGNC Symbol;Acc:416] |
| PLIN5 | ENSG00000214456 | perilipin 5 [Source:HGNC Symbol;Acc:33196] |
| ZNF705E | ENSG00000214534 | zinc finger protein 705E [Source:HGNC Symbol;Acc:33203] |
| MEG3 | ENSG00000214548 | maternally expressed 3 (non-protein coding) [Source:HGNC Symbol;Acc:14575] |
| SMTNL1 | ENSG00000214872 | smoothelin-like 1 [Source:HGNC Symbol;Acc:32394] |
| FAM177A1P1 | ENSG00000214886 | family with sequence similarity 177, member A1 pseudogene 1 [Source:HGNC Symbol;Acc:34441] |
| DNAJA1P3 | ENSG00000215007 | DnaJ (Hsp40) homolog, subfamily A, member 1 pseudogene 3 [Source:HGNC Symbol;Acc:39339] |
| CXorf49B | ENSG00000215113 | chromosome X open reading frame 49B [Source:HGNC Symbol;Acc:34229] |
| CXorf49 | ENSG00000215115 | chromosome X open reading frame 49 [Source:HGNC Symbol;Acc:30891] |
| LINC00680 | ENSG00000215190 | long intergenic non-protein coding RNA 680 [Source:HGNC Symbol;Acc:44417] |
| PSMA6P1 | ENSG00000215414 | proteasome (prosome, macropain) subunit, alpha type, 6 pseudogene 1 [Source:HGNC Symbol;Acc:17460] |
| TUBB2BP1 | ENSG00000216819 | tubulin, beta 2B class IIb pseudogene 1 [Source:HGNC Symbol;Acc:42332] |
| TENM3 | ENSG00000218336 | teneurin transmembrane protein 3 [Source:HGNC Symbol;Acc:29944] |
| HIST2H2BD | ENSG00000220323 | histone cluster 2, H2bd (pseudogene) [Source:HGNC Symbol;Acc:20517] |
| CEBPD | ENSG00000221869 | CCAAT/enhancer binding protein (C/EBP), delta [Source:HGNC Symbol;Acc:1835] |
| HMSD | ENSG00000221887 | histocompatibility (minor) serpin domain containing [Source:HGNC Symbol;Acc:23037] |
| APOL6 | ENSG00000221963 | apolipoprotein L, 6 [Source:HGNC Symbol;Acc:14870] |
| HIST2H2BA | ENSG00000223345 | histone cluster 2, H2ba (pseudogene) [Source:HGNC Symbol;Acc:20560] |
| HLA-DPB1 | ENSG00000223865 | major histocompatibility complex, class II, DP beta 1 [Source:HGNC Symbol;Acc:4940] |
| NBEAP3 | ENSG00000223875 | neurobeachin pseudogene 3 [Source:HGNC Symbol;Acc:40004] |
| LINC01133 | ENSG00000224259 | long intergenic non-protein coding RNA 1133 [Source:HGNC Symbol;Acc:49447] |
| IFIT6P | ENSG00000224289 | interferon-induced protein with tetratricopeptide repeats 6, pseudogene [Source:HGNC Symbol;Acc:38020] |
| C4B | ENSG00000224389 | complement component 4B (Chido blood group) [Source:HGNC Symbol;Acc:1324] |
| ZNF812 | ENSG00000224689 | zinc finger protein 812 [Source:HGNC Symbol;Acc:33242] |
| OR52K3P | ENSG00000225101 | olfactory receptor, family 52, subfamily K, member 3 pseudogene [Source:HGNC Symbol;Acc:15224] |
| PSME2P2 | ENSG00000225131 | proteasome activator subunit 2 pseudogene 2 [Source:HGNC Symbol;Acc:30160] |
| HSPA7 | ENSG00000225217 | heat shock 70kDa protein 7 (HSP70B) [Source:HGNC Symbol;Acc:5240] |
| ASS1P11 | ENSG00000225308 | argininosuccinate synthetase 1 pseudogene 11 [Source:HGNC Symbol;Acc:761] |
| GBP1P1 | ENSG00000225492 | guanylate binding protein 1, interferon-inducible pseudogene 1 [Source:HGNC Symbol;Acc:39561] |
| SP3P | ENSG00000225530 | Sp3 transcription factor pseudogene [Source:HGNC Symbol;Acc:35430] |
| MIAT | ENSG00000225783 | myocardial infarction associated transcript (non-protein coding) [Source:HGNC Symbol;Acc:33425] |
| CTAGE4 | ENSG00000225932 | CTAGE family, member 4 [Source:HGNC Symbol;Acc:24772] |
| LINC00869 | ENSG00000226067 | long intergenic non-protein coding RNA 869 [Source:HGNC Symbol;Acc:29050] |
| CFLAR-AS1 | ENSG00000226312 | CFLAR antisense RNA 1 [Source:HGNC Symbol;Acc:14437] |
| ITGB2-AS1 | ENSG00000227039 | ITGB2 antisense RNA 1 [Source:HGNC Symbol;Acc:44304] |
| OR2A9P | ENSG00000228960 | olfactory receptor, family 2, subfamily A, member 9 pseudogene [Source:HGNC Symbol;Acc:8236] |
| NBPF18P | ENSG00000229021 | neuroblastoma breakpoint family, member 18, pseudogene [Source:HGNC Symbol;Acc:31998] |
| SPRR2C | ENSG00000229035 | small proline-rich protein 2C (pseudogene) [Source:HGNC Symbol;Acc:11263] |
| PSMA6P2 | ENSG00000229083 | proteasome (prosome, macropain) subunit, alpha type, 6 pseudogene 2 [Source:HGNC Symbol;Acc:39607] |
| ANKRD10-IT1 | ENSG00000229152 | ANKRD10 intronic transcript 1 (non-protein coding) [Source:HGNC Symbol;Acc:39891] |
| PNPT1P1 | ENSG00000229241 | polyribonucleotide nucleotidyltransferase 1 pseudogene 1 [Source:HGNC Symbol;Acc:44468] |
| HLA-DRB6 | ENSG00000229391 | major histocompatibility complex, class II, DR beta 6 (pseudogene) [Source:HGNC Symbol;Acc:4954] |
| PATL2 | ENSG00000229474 | protein associated with topoisomerase II homolog 2 (yeast) [Source:HGNC Symbol;Acc:33630] |
| NAMPTL | ENSG00000229644 | nicotinamide phosphoribosyltransferase-like [Source:HGNC Symbol;Acc:17633] |
| XIST | ENSG00000229807 | X inactive specific transcript (non-protein coding) [Source:HGNC Symbol;Acc:12810] |
| HLA-DPA1 | ENSG00000231389 | major histocompatibility complex, class II, DP alpha 1 [Source:HGNC Symbol;Acc:4938] |
| CYP21A2 | ENSG00000231852 | cytochrome P450, family 21, subfamily A, polypeptide 2 [Source:HGNC Symbol;Acc:2600] |
| TAPBP | ENSG00000231925 | TAP binding protein (tapasin) [Source:HGNC Symbol;Acc:11566] |
| BDH2P1 | ENSG00000232699 | 3-hydroxybutyrate dehydrogenase, type 2 pseudogene 1 [Source:HGNC Symbol;Acc:21476] |
| TNF | ENSG00000232810 | tumor necrosis factor [Source:HGNC Symbol;Acc:11892] |
| LINC00472 | ENSG00000233237 | long intergenic non-protein coding RNA 472 [Source:HGNC Symbol;Acc:21380] |
| MTND4P20 | ENSG00000233377 | MT-ND4 pseudogene 20 [Source:HGNC Symbol;Acc:42207] |
| RAD1P2 | ENSG00000233473 | RAD1 homolog pseudogene 2 [Source:HGNC Symbol;Acc:49480] |
| SLC26A4-AS1 | ENSG00000233705 | SLC26A4 antisense RNA 1 [Source:HGNC Symbol;Acc:22385] |
| LINC01122 | ENSG00000233723 | long intergenic non-protein coding RNA 1122 [Source:HGNC Symbol;Acc:49267] |
| KRTAP5-AS1 | ENSG00000233930 | KRTAP5-1/KRTAP5-2 antisense RNA 1 [Source:HGNC Symbol;Acc:27877] |
| TRIM26 | ENSG00000234127 | tripartite motif containing 26 [Source:HGNC Symbol;Acc:12962] |
| H2BFS | ENSG00000234289 | H2B histone family, member S (pseudogene) [Source:HGNC Symbol;Acc:4762] |
| HLA-B | ENSG00000234745 | major histocompatibility complex, class I, B [Source:HGNC Symbol;Acc:4932] |
| MIR155HG | ENSG00000234883 | MIR155 host gene (non-protein coding) [Source:HGNC Symbol;Acc:35460] |
| ZSCAN31 | ENSG00000235109 | zinc finger and SCAN domain containing 31 [Source:HGNC Symbol;Acc:14097] |
| LINC00623 | ENSG00000235398 | long intergenic non-protein coding RNA 623 [Source:HGNC Symbol;Acc:44252] |
| KIAA0040 | ENSG00000235750 | KIAA0040 [Source:HGNC Symbol;Acc:28950] |
| DIAPH2-AS1 | ENSG00000236256 | DIAPH2 antisense RNA 1 [Source:HGNC Symbol;Acc:16972] |
| LINC00854 | ENSG00000236383 | long intergenic non-protein coding RNA 854 [Source:HGNC Symbol;Acc:43658] |
| ARHGEF38 | ENSG00000236699 | Rho guanine nucleotide exchange factor (GEF) 38 [Source:HGNC Symbol;Acc:25968] |
| CTAGE9 | ENSG00000236761 | CTAGE family, member 9 [Source:HGNC Symbol;Acc:37275] |
| C5orf27 | ENSG00000236882 | chromosome 5 open reading frame 27 [Source:HGNC Symbol;Acc:24687] |
| ANO7P1 | ENSG00000237276 | anoctamin 7 pseudogene 1 [Source:HGNC Symbol;Acc:32248] |
| NDUFA9P1 | ENSG00000237406 | NADH dehydrogenase (ubiquinone) 1 alpha subcomplex, 9, pseudogene 1 [Source:HGNC Symbol;Acc:19252] |
| TTLL11-IT1 | ENSG00000237548 | TTLL11 intronic transcript 1 (non-protein coding) [Source:HGNC Symbol;Acc:24214] |
| RAC1P4 | ENSG00000237584 | ras-related C3 botulinum toxin substrate 1 pseudogene 4 [Source:HGNC Symbol;Acc:31113] |
| S100A11P1 | ENSG00000237632 | S100 calcium binding protein A11 pseudogene 1 [Source:HGNC Symbol;Acc:10491] |
| PARP4P1 | ENSG00000237917 | poly (ADP-ribose) polymerase family, member 4 pseudogene 1 [Source:HGNC Symbol;Acc:18500] |
| GSTTP2 | ENSG00000239334 | glutathione S-transferase theta pseudogene 2 [Source:HGNC Symbol;Acc:33606] |
| APOBEC3G | ENSG00000239713 | apolipoprotein B mRNA editing enzyme, catalytic polypeptide-like 3G [Source:HGNC Symbol;Acc:17357] |
| PSMB9 | ENSG00000240065 | proteasome (prosome, macropain) subunit, beta type, 9 [Source:HGNC Symbol;Acc:9546] |
| CPHL1P | ENSG00000240216 | ceruloplasmin and hephaestin-like 1 pseudogene [Source:HGNC Symbol;Acc:31714] |
| KRBOX1 | ENSG00000240747 | KRAB box domain containing 1 [Source:HGNC Symbol;Acc:38708] |
| HLA-DOB | ENSG00000241106 | major histocompatibility complex, class II, DO beta [Source:HGNC Symbol;Acc:4937] |
| RPSAP52 | ENSG00000241749 | ribosomal protein SA pseudogene 52 [Source:HGNC Symbol;Acc:35752] |
| SPRR2A | ENSG00000241794 | small proline-rich protein 2A [Source:HGNC Symbol;Acc:11261] |
| PLEKHO2 | ENSG00000241839 | pleckstrin homology domain containing, family O member 2 [Source:HGNC Symbol;Acc:30026] |
| RPS26P35 | ENSG00000242206 | ribosomal protein S26 pseudogene 35 [Source:HGNC Symbol;Acc:36229] |
| UGT1A10 | ENSG00000242515 | UDP glucuronosyltransferase 1 family, polypeptide A10 [Source:HGNC Symbol;Acc:12531] |
| SERPINB10 | ENSG00000242550 | serpin peptidase inhibitor, clade B (ovalbumin), member 10 [Source:HGNC Symbol;Acc:8942] |
| HLA-DMB | ENSG00000242574 | major histocompatibility complex, class II, DM beta [Source:HGNC Symbol;Acc:4935] |
| MBL1P | ENSG00000242600 | mannose-binding lectin (protein A) 1, pseudogene [Source:HGNC Symbol;Acc:6921] |
| CFB | ENSG00000243649 | complement factor B [Source:HGNC Symbol;Acc:1037] |
| HLA-L | ENSG00000243753 | major histocompatibility complex, class I, L (pseudogene) [Source:HGNC Symbol;Acc:4970] |
| APOBEC3D | ENSG00000243811 | apolipoprotein B mRNA editing enzyme, catalytic polypeptide-like 3D [Source:HGNC Symbol;Acc:17354] |
| SPRR2F | ENSG00000244094 | small proline-rich protein 2F [Source:HGNC Symbol;Acc:11266] |
| PBX2P1 | ENSG00000244171 | pre-B-cell leukemia homeobox 2 pseudogene 1 [Source:HGNC Symbol;Acc:8635] |
| CFHR1 | ENSG00000244414 | complement factor H-related 1 [Source:HGNC Symbol;Acc:4888] |
| OR2A1-AS1 | ENSG00000244479 | OR2A1 antisense RNA 1 [Source:HGNC Symbol;Acc:49168] |
| CTAGE8 | ENSG00000244693 | CTAGE family, member 8 [Source:HGNC Symbol;Acc:37294] |
| C4A | ENSG00000244731 | complement component 4A (Rodgers blood group) [Source:HGNC Symbol;Acc:1323] |
| LIFR-AS1 | ENSG00000244968 | LIFR antisense RNA 1 [Source:HGNC Symbol;Acc:43600] |
| MIR3150A | ENSG00000245080 | microRNA 3150a [Source:HGNC Symbol;Acc:38362] |
| NEAT1 | ENSG00000245532 | nuclear paraspeckle assembly transcript 1 (non-protein coding) [Source:HGNC Symbol;Acc:30815] |
| C14orf64 | ENSG00000246223 | chromosome 14 open reading frame 64 [Source:HGNC Symbol;Acc:20111] |
| SOCS2-AS1 | ENSG00000246985 | SOCS2 antisense RNA 1 [Source:HGNC Symbol;Acc:27054] |
| NR2F2-AS1 | ENSG00000247809 | NR2F2 antisense RNA 1 [Source:HGNC Symbol;Acc:44222] |
| LUCAT1 | ENSG00000248323 | lung cancer associated transcript 1 (non-protein coding) [Source:HGNC Symbol;Acc:48498] |
| PCP4L1 | ENSG00000248485 | Purkinje cell protein 4 like 1 [Source:HGNC Symbol;Acc:20448] |
| KRT8P46 | ENSG00000248971 | keratin 8 pseudogene 46 [Source:HGNC Symbol;Acc:39880] |
| ATP1B1P1 | ENSG00000249212 | ATPase, Na+/K+ transporting, beta 1 polypeptide pseudogene 1 [Source:HGNC Symbol;Acc:809] |
| UNC93B8 | ENSG00000249272 | unc-93 homolog B8 pseudogene (C. elegans) [Source:HGNC Symbol;Acc:44037] |
| IL20RB-AS1 | ENSG00000249407 | IL20RB antisense RNA 1 [Source:HGNC Symbol;Acc:40298] |
| LINC00536 | ENSG00000249917 | long intergenic non-protein coding RNA 536 [Source:HGNC Symbol;Acc:43645] |
| TRIM60P14 | ENSG00000250227 | tripartite motif containing 60 pseudogene 14 [Source:HGNC Symbol;Acc:38486] |
| ZNF718 | ENSG00000250312 | zinc finger protein 718 [Source:HGNC Symbol;Acc:26889] |
| PSMA2P2 | ENSG00000251234 | proteasome (prosome, macropain) subunit, alpha type, 2 pseudogene 2 [Source:HGNC Symbol;Acc:43833] |
| MALAT1 | ENSG00000251562 | metastasis associated lung adenocarcinoma transcript 1 (non-protein coding) [Source:HGNC Symbol;Acc:29665] |
| UNC93B7 | ENSG00000251624 | unc-93 homolog B7 pseudogene (C. elegans) [Source:HGNC Symbol;Acc:44036] |
| CCDC71L | ENSG00000253276 | coiled-coil domain containing 71-like [Source:HGNC Symbol;Acc:26685] |
| MIR146A | ENSG00000253522 | microRNA 146a [Source:HGNC Symbol;Acc:31533] |
| PCDHGA7 | ENSG00000253537 | protocadherin gamma subfamily A, 7 [Source:HGNC Symbol;Acc:8705] |
| CLDN23 | ENSG00000253958 | claudin 23 [Source:HGNC Symbol;Acc:17591] |
| LYN | ENSG00000254087 | v-yes-1 Yamaguchi sarcoma viral related oncogene homolog [Source:HGNC Symbol;Acc:6735] |
| PCDHGA3 | ENSG00000254245 | protocadherin gamma subfamily A, 3 [Source:HGNC Symbol;Acc:8701] |
| CASP1P2 | ENSG00000254750 | caspase 1, apoptosis-related cysteine peptidase pseudogene 2 [Source:HGNC Symbol;Acc:43776] |
| GVINP1 | ENSG00000254838 | GTPase, very large interferon inducible pseudogene 1 [Source:HGNC Symbol;Acc:25813] |
| CARD17 | ENSG00000255221 | caspase recruitment domain family, member 17 [Source:HGNC Symbol;Acc:33827] |
| HCAR3 | ENSG00000255398 | hydroxycarboxylic acid receptor 3 [Source:HGNC Symbol;Acc:16824] |
| UNC93B6 | ENSG00000255562 | unc-93 homolog B6 (C. elegans) [Source:HGNC Symbol;Acc:34053] |
| KRT8P11 | ENSG00000255815 | keratin 8 pseudogene 11 [Source:HGNC Symbol;Acc:31058] |
| MT1JP | ENSG00000255986 | metallothionein 1J, pseudogene [Source:HGNC Symbol;Acc:7402] |
| CTSO | ENSG00000256043 | cathepsin O [Source:HGNC Symbol;Acc:2542] |
| USP30-AS1 | ENSG00000256262 | USP30 antisense RNA 1 [Source:HGNC Symbol;Acc:40909] |
| ZNF350 | ENSG00000256683 | zinc finger protein 350 [Source:HGNC Symbol;Acc:16656] |
| CAPNS2 | ENSG00000256812 | calpain, small subunit 2 [Source:HGNC Symbol;Acc:16371] |
| C12orf79 | ENSG00000257242 | chromosome 12 open reading frame 79 [Source:HGNC Symbol;Acc:27409] |
| FAHD2P1 | ENSG00000257302 | fumarylacetoacetate hydrolase domain containing 2 pseudogene 1 [Source:HGNC Symbol;Acc:32441] |
| KRT8P19 | ENSG00000257528 | keratin 8 pseudogene 19 [Source:HGNC Symbol;Acc:33371] |
| LINC00519 | ENSG00000258955 | long intergenic non-protein coding RNA 519 [Source:HGNC Symbol;Acc:19838] |
| THTPA | ENSG00000259431 | thiamine triphosphatase [Source:HGNC Symbol;Acc:18987] |
| RAB43P1 | ENSG00000259856 | RAB43 pseudogene 1 [Source:HGNC Symbol;Acc:33153] |
| LINC00890 | ENSG00000260802 | long intergenic non-protein coding RNA 890 [Source:HGNC Symbol;Acc:48576] |
| FEM1AP2 | ENSG00000265296 | fem-1 homolog a (C. elegans) pseudogene 2 [Source:HGNC Symbol;Acc:39830] |
| FSBP | ENSG00000265817 | fibrinogen silencer binding protein [Source:HGNC Symbol;Acc:43653] |
| LINC00669 | ENSG00000267374 | long intergenic non-protein coding RNA 669 [Source:HGNC Symbol;Acc:44332] |
| NFE2L3P1 | ENSG00000267497 | nuclear factor, erythroid 2-like 3 pseudogene 1 [Source:HGNC Symbol;Acc:22278] |
| S1PR2 | ENSG00000267534 | sphingosine-1-phosphate receptor 2 [Source:HGNC Symbol;Acc:3169] |
| SNX6P1 | ENSG00000267920 | sorting nexin 6 pseudogene 1 [Source:HGNC Symbol;Acc:41511] |
| IFNL3P1 | ENSG00000268510 | interferon, lambda 3 pseudogene 1 [Source:HGNC Symbol;Acc:44483] |
| CTAGE6 | ENSG00000271321 | CTAGE family, member 6 [Source:HGNC Symbol;Acc:28644] |
| MILR1 | ENSG00000271605 | mast cell immunoglobulin-like receptor 1 [Source:HGNC Symbol;Acc:27570] |
| DOC2B | ENSG00000272636 | double C2-like domains, beta [Source:HGNC Symbol;Acc:2986] |
